# Estimating the Range and Duration of Post-influenza Symptoms, Conditions, and Diseases

**DOI:** 10.1093/ofid/ofag629

**Published:** 2026-10-06

**Authors:** Aaron C Miller, D Erik Boonstra, Joseph E Cavanaugh, Roger D Struble, Constantina Boikos, Tianyan Hu, Timothy L Wiemken, Verna L Welch, Philip M Polgreen

**Affiliations:** Department of Internal Medicine, University of Iowa, Iowa City, Iowa, USA; Department of Biostatistics, University of Iowa, Iowa City, Iowa, USA; Department of Biostatistics, University of Iowa, Iowa City, Iowa, USA; Department of Internal Medicine, University of Iowa, Iowa City, Iowa, USA; Department of Medical Affairs, Pfizer Inc., Kirkland, QC, Canada; Pfizer Inc., New York, New York, USA; Department of Medical Affairs, Pfizer Inc., New York, New York, USA; Department of Medical Affairs, Pfizer Inc., New York, New York, USA; Department of Internal Medicine, University of Iowa, Iowa City, Iowa, USA

**Keywords:** influenza, sequelae, symptoms

## Abstract

**Background:**

Acute symptoms of influenza are understood; however, less is known about potential postacute sequelae. We conducted a retrospective matched cohort study to identify conditions with elevated risk following influenza.

**Methods:**

We analyzed data from the Merative MarketScan Research Databases (2001–2019), including 3.3 million patients with a diagnosis of influenza. Cases were matched 1:1 to patients based on similar age, sex, care setting, and enrollment period with no influenza diagnosis. We examined the frequency of diagnosis codes in the year before and after the index influenza event, applying K-means clustering to identify diagnoses with post-influenza visit patterns similar to cough, a known sequela of influenza. Conditions were reviewed and aggregated into clinical categories. We compared trends in diagnoses between influenza cases and controls using a difference-in-differences approach.

**Results:**

We identified 67 diagnostic categories with significantly increased incidence post-influenza infection. Both commonly known sequelae, such as pneumonia and otitis media as well as less expected findings, such as acute kidney failure and oral candidiasis were identified. The duration of elevated risk varied by condition, ranging from a week (eg, rhabdomyolysis) to 5 weeks (eg, hemoptysis) or 8 weeks (eg, chronic bronchitis).

**Conclusions:**

We found that influenza is associated with conditions not typically considered. Also, some symptoms last longer than commonly appreciated, suggesting that some patients may suffer from a prolonged illness due to the influenza infection. Our findings have important implications for not only demonstrating the full burden of influenza but also informing future studies to prevent or treat influenza.

## Teaser

Without prespecifying specific conditions, symptoms or their durations, we found that influenza is associated with a greater number of symptoms and acute sequelae than commonly appreciated, and some symptoms and sequelae last for substantial periods of time (ie, 5–8 weeks).

The clinical manifestations of influenza vary widely from asymptomatic infections to severe cases resulting in respiratory failure and death [1, 2]. For symptomatic cases, classic symptoms include fevers, chills, fatigue, myalgias, coughing, sneezing, nasal congestion, and sinus discomfort [3]. Typically symptoms are described as lasting from 3 to 8 days [4], but some symptoms (eg, coughing) may last longer (eg, 2 weeks) [4]. While some reports indicate that symptoms may last longer than 2 weeks, especially in complicated cases of influenza [3, 5], most reports do not quantify how long different symptoms associated with influenza generally last.

In addition to the symptoms classically associated with influenza, a subset of people develops a secondary bacterial infection (eg, pneumonia) [2, 6–13]. These secondary infections may prolong the overall duration of the illness and increase healthcare utilization and costs. For example, biphasic fevers can be associated with influenza [14]; the first fever is caused by influenza and the second is caused by the secondary bacterial infection. Beyond secondary bacterial infections, noninfectious complications (eg, acute myocardial infarctions and acute kidney injury) or exacerbation of pre-existing diseases (eg, COPD and asthma) are also associated with influenza [15–20]. Accordingly, the signs, symptoms and conditions attributable to an influenza infection may not only last longer than commonly considered, but also could include a more expansive list of signs and symptoms than typically considered for cases of uncomplicated influenza.

Given that the signs, symptoms, and conditions attributable to influenza may be both more expansive and last longer than typically considered [4], the burden of illness attributable to influenza may be much greater than prior estimates. Accordingly, the goal of this analysis was 2-fold. First, we conduct an exhaustive search of potential candidate signs, symptoms and conditions associated with cases of influenza. Second, we estimate the length of time for which these signs, symptoms and conditions remained elevated following an influenza infection. We focus on both the signs and symptoms associated with the initial infection that may continue as well as new signs or symptoms that follow the initial infection (ie, acute post-influenza sequelae).

## METHODS

### Data Source

We used data from the Merative MarketScan Commercial Claims and Encounters and Medicare Supplemental Databases from 2001 to 2019. Note, we did not include the years after 2019 to avoid inclusion of the COVID-19 pandemic. These databases contain longitudinal insurance claims data from over 200 million enrollees across the United States with employer-sponsored commercial health insurance, supplemental Medicare plans, or state Medicaid coverage. Claims are provided for inpatient, outpatient, and emergency department encounters along with outpatient-prescription medications.

### Study Design and Population

We conducted a retrospective study that utilized a difference-in-difference (DiD) approach to compare healthcare utilization patterns between a cohort of patients diagnosed with influenza to a matched cohort of patients without influenza. Healthcare utilization patterns among both cohorts were analyzed for 1 year before and after the influenza event (or respective event date for controls).

To construct roughly comparable analytic cohorts from the full MarketScan population, we used matching primarily as a data-reduction step. Specifically, influenza cases were matched 1:1 to individuals without influenza on key enrollment factors including age, sex, influenza event timing (month/year), and care setting, while requiring continuous enrollment before and after the index event. This approach restricted the analysis to a subset of patients with similar enrollment characteristics and follow-up structure, improving comparability and computational feasibility while preserving the temporal structure needed for pre–post analyses.

To address residual confounding, particularly differences in baseline healthcare utilization, we applied a DiD design. In this framework, each group (influenza cases and matched controls) contributes to both a preindex (baseline) and postindex period. The DiD estimator quantifies the change in outcome (eg, visit frequency for a given condition) from preindex to postindex among cases, and then subtracts the corresponding change observed in controls. By focusing on within-group changes over time and comparing those changes across groups, the DiD approach removes bias from stable, baseline differences between cases and controls (eg, higher underlying healthcare use among cases) and from shared temporal trends affecting both groups (eg, seasonality or healthcare-seeking behavior). The resulting estimate isolates the excess change attributable to influenza beyond what would be expected based on baseline differences and background trends.

#### Influenza (case) Cohort

All inpatient or outpatient influenza visits that occurred between January 1, 2002 and December 31, 2018 were identified using the diagnosis codes in Supplementary Appendix Table 1. Second, we identified the initial influenza visit for each patient and restricted the selection to those patients who were continuously enrolled 1 year before and after their initial influenza diagnosis. Next, we roughly classified whether the influenza event constituted an inpatient or outpatient event. Inpatient influenza cases were defined by either (1) an index influenza diagnosis that occurred in an inpatient setting or (2) an inpatient admission involving an influenza diagnosis within 14 days after the initial outpatient influenza diagnosis. Outpatient influenza cases were defined as influenza cases diagnosed in an outpatient setting that did not involve an inpatient influenza hospitalization within the year following the index influenza diagnosis. To exclude influenza-related hospitalizations that did not contain an influenza-specific diagnosis code, we excluded outpatient cases if there was an inpatient visit for any reason within 7 days of the outpatient influenza diagnosis. With the identification of the initial inpatient and outpatient influenza events for each patient, we excluded any subsequent influenza event that occurred during the study period.

#### Non-influenza (control) Cohort

The potential control cohort was identified using all inpatient and outpatient visits from patients not included among influenza cases who could be followed for 1 year before and after the given visit. For each patient in the case cohort, we selected a patient from the potential control cohort using the following criteria: (1) had a healthcare visit of a similar type (inpatient or outpatient) in the same year and month as the corresponding index influenza visit; this was labeled the *matched-index event*; (2) was associated with the same sex and age group (ie, within 5 years) as the corresponding case patient. Note, matching was only used to identify a temporally comparable control population, while the DiD design was used to account for underlying differences in healthcare utilization.

After selecting both the case and matched-control cohorts, we identified all visits that occurred within 1 year before and after the index influenza event for the case cohort, or corresponding matched-index event for the control cohort. The frequency of all diagnoses that occurred during these healthcare visits were recorded.

### Identification of Relevant Conditions and Estimation of Postacute Period

Conditions that may represent postacute sequelae of influenza should exhibit a pattern of greater frequency following the index infection date relative to both (1) the preinfection period and (2) matched controls. We used a 3-stage process to systematically identify relevant conditions representing postacute sequelae and estimate the period in which the condition was elevated following influenza. Below is a brief overview of this 3-stage selection and estimation process. See Supplementary Appendix Methods 1–3 for additional details.

#### Stage 1: Clustering diagnosis codes

The first stage involved clustering diagnosis codes based on healthcare utilization patterns surrounding the influenza diagnosis. We analyzed the 2000 most frequently occurring ICD-9 and ICD-10 codes by calculating daily visit frequencies before and after influenza onset. Using K-means clustering, we grouped diagnosis codes with similar temporal patterns. Conditions were flagged as potential sequelae if they either (1) clustered with “cough” (a known post-influenza symptom) or (2) the diagnoses in the cluster exhibited a distinct post-influenza visit pattern—specifically, a rise in visit frequency following the influenza diagnosis followed by a return to baseline comparable to that observed over the year prior to diagnosis. Because this was an exploratory process, we evaluated clusters using values of *k* from 3 to 30. The frequency with which a code clustered with another was evaluated by averaging over results from *k* = 3–30.

#### Stage 2: Estimation of Increased Frequency Following Influenza

In the second stage, we applied a DiD approach to evaluate the extent to which visit frequencies for the conditions identified in Stage 1 were elevated after influenza relative to matched controls. This analysis identified diagnosis codes with patterns consistent with post-influenza elevation. The DiD design minimizes bias from underlying cohort differences, general follow-up, or surveillance effects (eg, healthcare-seeking behavior) among the influenza cohort by removing baseline differences in healthcare utilization between influenza cases and controls. The primary outcome was the number of patients in each cohort with a visit for the condition during a given week following influenza. The model controlled for confounding factors such as case status, week number relative to index, age group, sex, index month, and year. The weekly visit counts were aggregated into these corresponding strata. The primary effect of interest was captured by an interaction term between case status (vs control) and the week number following influenza. An offset term was used to account for enrollment in each stratum. A quasi-Poisson regression model was used to evaluate whether each condition showed a statistically significant increase in visits during the post-influenza period.

#### Stage 3: Selection of Clinically Relevant Codes, Aggregation Into Categories, and Estimating Timing

The final stage focused on clinical validation and temporal characterization of postacute sequelae. Two reviewers (an infectious disease physician and a pulmonary critical care physician) independently evaluated each condition identified in Stage 2 for clinical plausibility and resolved discrepancies via consensus. Both reviewers had to agree on the inclusion of a symptom or condition. Validated ICD codes were then aggregated into clinically meaningful categories, across both ICD-9 and ICD-10 formats. We reran the DiD model for each aggregated group and used the resulting model estimates to quantify the duration of post-influenza elevation. We first determined if visits for the condition eventually returned to either (1) a pre-influenza baseline with no statistically significant difference between cases and controls or (2) a new baseline where the condition remained elevated among cases relative to controls. Finally, we calculated the number of consecutive weeks where the condition remained elevated relative to this corresponding baseline.

## RESULTS

We identified a total of 3 345 768 patients with influenza from 2002 to 2018 who could be followed for at least 1 year before and after the index influenza event. These cases were categorized into 53 750 inpatient influenza cases and 3 274 105 outpatient cases; 17 913 did not meet our outpatient or inpatient case definition and were excluded. Each of these included cases were then matched 1:1 to a patient who had a non-influenza related visit in the same setting (inpatient or outpatient) and in the same month/year as the matched influenza index date. Figure 1 provides a flow diagram of patients included in our analysis.

**Figure 1.**
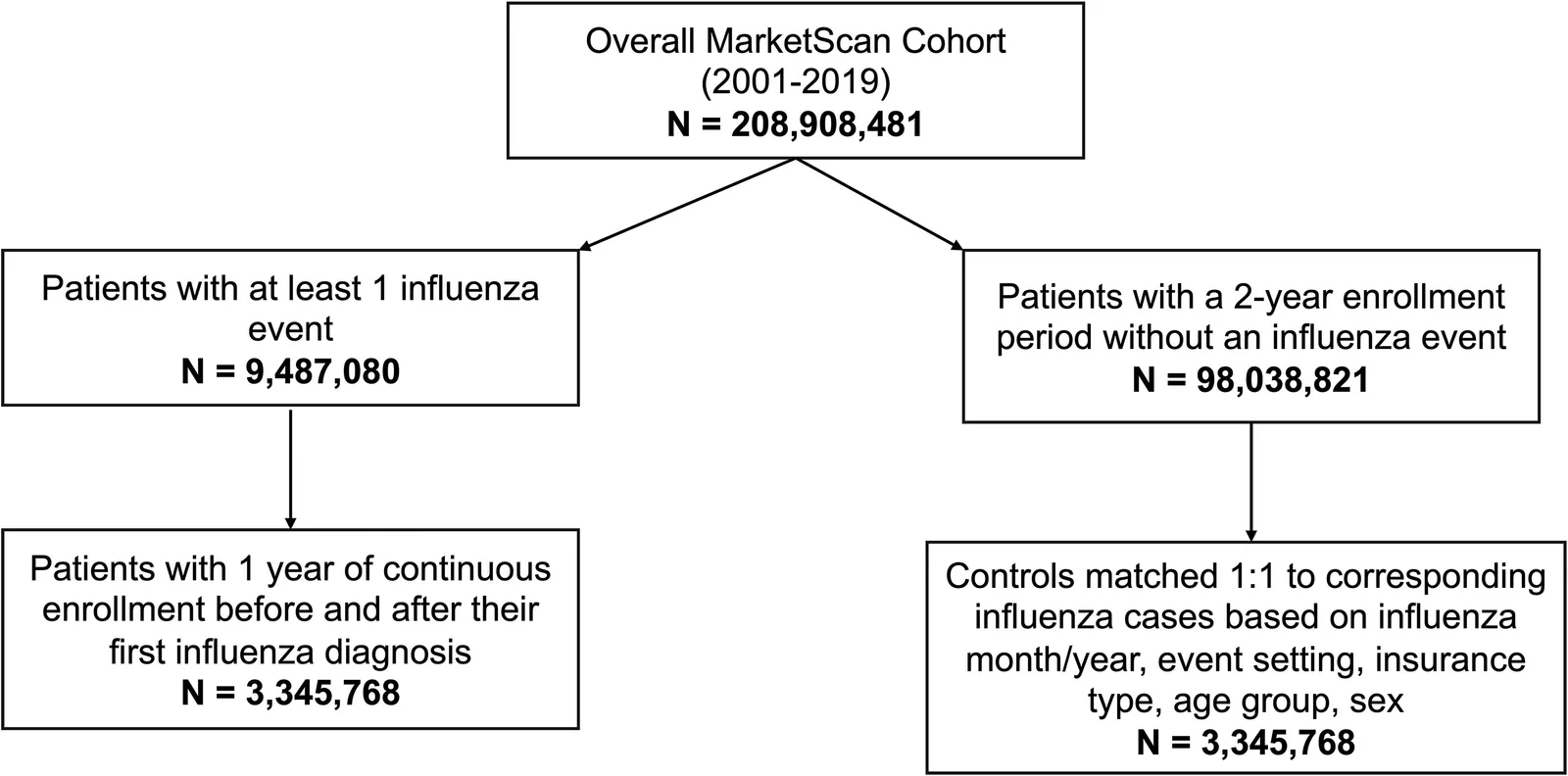
Flow chart summarizing the number of cases included based on applying the study inclusion criteria and matching process.

Figure 2 depicts the trend in the total number of healthcare visits each week before and after the index event date for both cases and controls. There are 2 notable features in this plot. First, compared to control patients, case patients had a greater average healthcare utilization each week before and after the index diagnosis. Second, compared with influenza patients, control patients exhibited a more seasonal cyclical pattern in healthcare utilization. This trend peaked near the index event (ie, typically during the peak of influenza season), decreased for about 6 months before or after the index event, and then increased up to the year before or after the index event (ie, the following or next respiratory-infection period).

**Figure 2.**
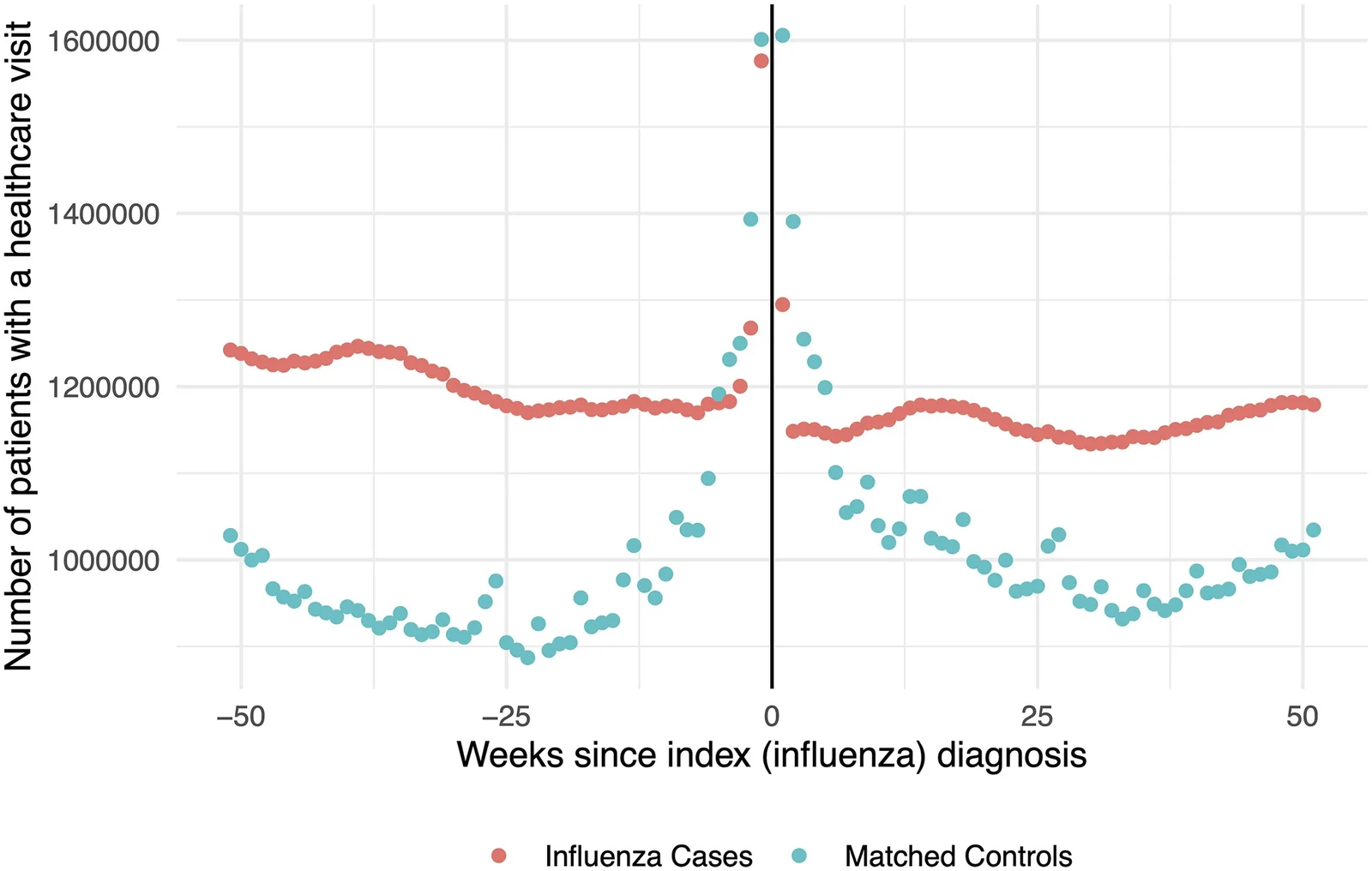
Trends in healthcare visits prior to and after the index influenza diagnosis (cases) or corresponding visit event (controls). The total number of patients with a healthcare visit in a given week (relative to the index influenza event) is plotted for influenza cases and controls. Cases are depicted in red and controls are depicted in blue.

### Identification of Relevant Conditions

We initially identified 251 individual ICD-9 codes, and 264 individual ICD-10 codes that may represent potential postacute sequelae following influenza (see Supplementary Appendix Tables 2 and 3). Supplementary Appendix Figures A1-A28 depict the trends of the clusters that were identified for ICD-9 codes and Supplementary Appendix Figures B1-B28 depict the trends of the clusters identified for ICD-10 codes. Supplementary Appendix Tables 2 and 3 present the codes that cluster with cough, using ICD-9 and ICD-10 codes, respectively. This table also provides the fraction of the time that a given code appeared in the focal cluster when various values of *k* from 3 to 30 were considered; the codes identified were ordered by this value.

Supplementary Appendix Figures C and D depict the cluster shapes that were identified for ICD-9 and ICD-10 codes, respectively. Each of these figures highlight the plausible conditions that appeared to exhibit an increased trend for a period following influenza activity compared with the period prior to influenza.

### Estimation of Increased Frequency and Timing for Potential Postacute Sequelae

Supplementary Appendix Figure E depicts results of parameter estimates for the weekly interaction term for cases versus controls from the DiD analysis for a handful of selected ICD-9 conditions, where there was a persistent increased trend for the weeks following the index influenza diagnosis. Specifically, Supplementary Appendix Figure E demonstrates that acute bronchitis, pneumonia unspecified, fever, and cough were each elevated for around 4, 10, 4, and 7 weeks, respectively. Supplementary Appendix Figures F and G depict results of the DiD analysis for all the identified ICD-9 and ICD-10 conditions, respectively. We identified 145 ICD-9 conditions and 111 ICD-10 conditions that were significantly elevated following influenza. In addition to commonly recognized sequelae such as pneumonia, and COPD exacerbations, we also uncovered other conditions such as candidiasis of mouth and acute kidney failure.

### Aggregating Clinically Relevant Conditions and Identifying Timing for Postacute Sequelae

Review of the 145 ICD-9 conditions and 111 ICD-10 conditions led to the exclusion of 8 ICD-9 codes and 9 ICD-10 codes. (See Supplementary Appendix Table 4 and 5) The remaining codes were then aggregated into 80 different diagnostic categories (see Supplementary Appendix Table 6). We then repeated the DiD analysis using these aggregated condition codes. These results were rereviewed, and thirteen of the 80 conditions were removed due to patterns identified in the DiD analysis that were inconsistent with postacute sequelae (see Supplementary Appendix Table 6), leaving 67 diagnostic conditions that were consistently elevated after influenza infection.

Figure 3 depicts the results of the DiD analysis in terms of the parameter estimates for the weekly interaction term between cases and controls for the conditions of acute bronchitis, cough, fever and pneumonia. The duration of elevated risk varied by condition, ranging from a week (eg, throat pain, rhabdomyolysis, and otitis media) to 5 weeks (eg, hemoptysis and painful respiration/pleurisy) or 8 weeks (eg, chronic bronchitis and emphysema). Supplementary Appendix Figures H depict the DiD results for the remaining aggregated conditions.

**Figure 3.**
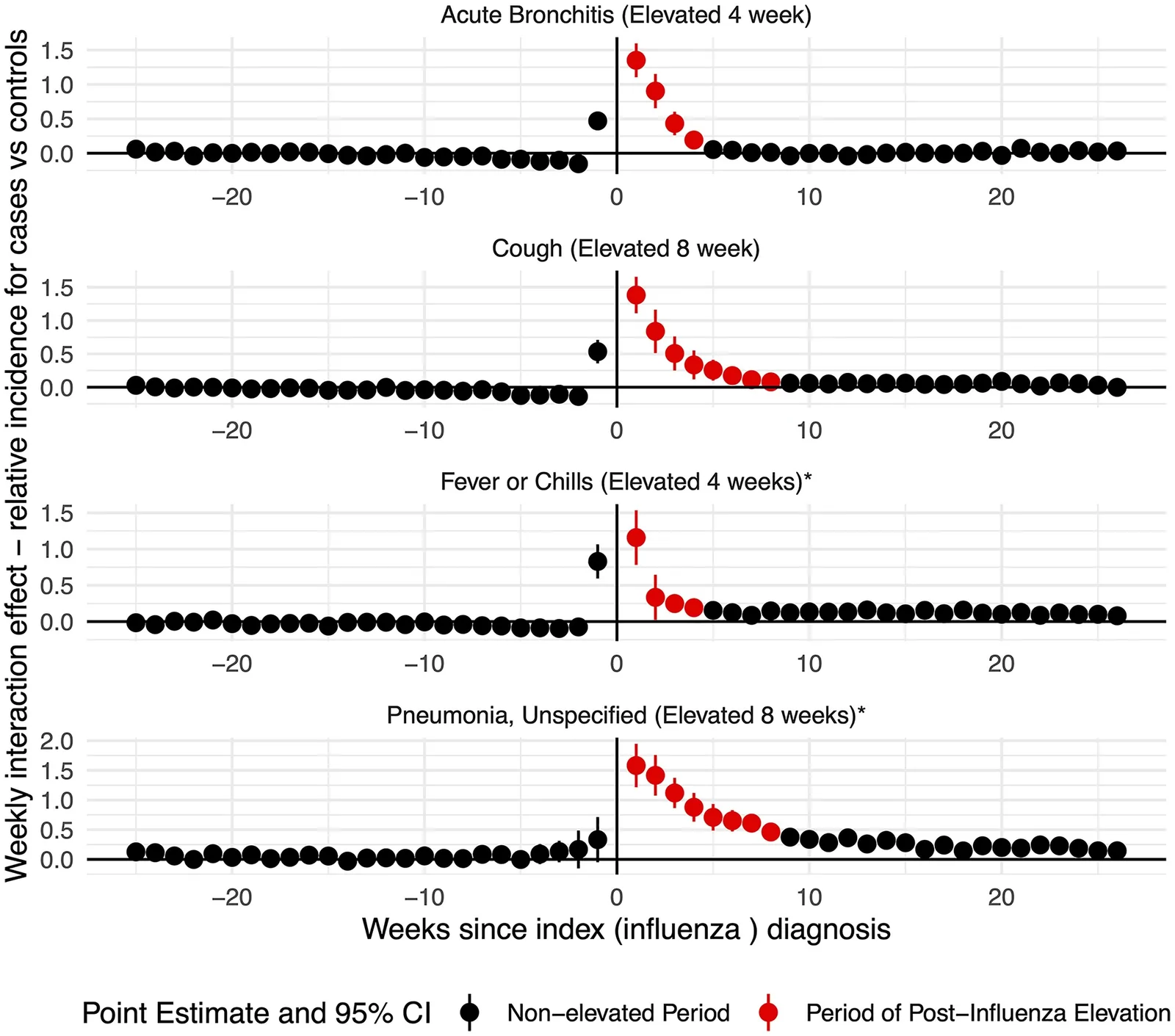
DiD results for the weekly interaction term between cases and controls following influenza for the selected conditions of acute bronchitis, cough, fever or chills, and pneumonia. See Supplementary Appendix Figures H for all 80 condition groups. The parameter estimate for each weekly interaction term is plotted by the dot, with 95% confidence intervals depicted by the line segment. The effect estimate for the relative difference in weekly incidence is plotted on the y-axis. The number of weeks for which the condition was elevated following influenza is highlighted in red. The weeks of elevation were determined by the number of consecutive weeks post-influenza where the effect estimate was significantly greater than zero (conditions noted with “*” were deemed to return to a higher baseline following influenza and the CUSUM approach was used to identify this point). See Supplementary Appendix Methods 3 for additional details regarding the approach used to identify weeks of elevation.

Table 1 summarizes the findings from our final DiD analysis for the final 67 conditions that were identified. Table 1 reports the duration of time the condition was elevated and if it returned to a higher baseline following infection (the approach used to identify this time is also noted). Table 1 also reports the corresponding case-and-control incidence during the elevated post-influenza period as well as the incidence per 100 000 people. The conditions with the highest incidence were respiratory including acute bronchitis, acute sinusitis, pneumonia, and asthma. Twenty-five of the 67 conditions were nonrespiratory. Some of these conditions were symptoms typically associated with influenza (eg, fever, malaise and fatigue). In addition, many of the other nonrespiratory conditions are commonly associated with severe illness such as thrombocytopenia, acute kidney failure, electrolyte and fluid disorders, hypotension, leukocytosis, leukopenia, neutropenia, pancytopenia, and altered mental status. These nonrespiratory conditions were less common than the respiratory-related conditions. Some of the nonrespiratory conditions may be associated with influenza treatment or related conditions (eg, oral candidiasis, adverse drug events).

**Table 1.** Summary of Findings From the DiD Models: Conditions/Syndromes That Represent Potential Postacute Sequelae Following Influenza

Condition/Syndrome	Weeks Elevated*	Visit Counts/Incidence During Corresponding Period of Elevated Frequency Following Influenza			
		Case Count	Case Incidence (per 100 K cases)^a^	Control Count	Control Incidence (per 100 K cases)^a^
Symptoms/clinical signs					
Abnormal chest sounds	3	1909	58.31	610	18.63
Abnormal sputum	1	90	2.75	12	0.37
Altered mental status	3	1086	33.17	604	18.45
Cough	8	156 534	4780.97	53 086	1621.39
Fever or chills	4*	62 739	1916.22	19 377	591.83
Hemoptysis	5	1422	43.43	316	9.65
Malaise and fatigue	6*	34 950	1067.47	20 029	611.74
Nausea or vomiting	1	12 077	368.86	4624	141.23
Pain, unspecified	1	760	23.21	367	11.21
Painful respiration or pleurisy	5	4705	143.7	1883	57.51
Shortness of breath	3*	26 358	805.04	11 722	358.02
Tachycardia	1	1454	44.41	733	22.39
Tachypnea	1	109	3.33	23	0.7
Throat pain	1	1565	47.8	550	16.8
Wheezing	3	9877	301.67	3185	97.28
Infectious diseases					
Acute bronchiolitis, other specified organisms or unspecified	1	1696	51.8	763	23.3
Acute laryngitis, laryngopharyngitis, or tracheitis	1	594	18.14	174	5.31
Acute nasopharyngitis or pharyngitis	1	31 274	955.19	11 067	338.02
Acute suppurative or nonsuppurative or unspecified otitis media	1	12 760	389.72	4985	152.26
Acute sinusitis	2	50 952	1556.21	15 978	488.01
Acute or unspecified bronchitis	4	96 706	2953.66	23 275	710.88
Acute upper respiratory infections of multiple or unspecified sites	1	32 379	988.94	11 357	346.87
Bacteremia	1	270	8.25	114	3.48
Bacterial pneumonia	7	10 868	331.94	2484	75.87
Chronic bronchitis	8	7353	224.58	2798	85.46
Chronic sinusitis	2	14 268	435.78	7050	215.33
Herpes viral infection, unspecified	1	349	10.66	180	5.5
Oral Candidiasis	5	1765	53.91	670	20.46
Other viral infections	2	25 168	768.7	8405	256.71
Pneumonia, unspecified	8*	50 469	1541.46	12 215	373.08
Streptococcal sore throat	1	2922	89.25	1472	44.96
Streptococcus infections, unspecified	2	96	2.93	52	1.59
Respiratory conditions (noninfectious)					
Acute bronchospasm	5*	6101	186.34	1839	56.17
Acute or acute and chronic respiratory failure	6*	1765	53.91	902	27.55
Asthma	3*	46 865	1431.38	22 094	674.81
Atelectasis or pulmonary collapse	5	3489	106.56	1531	46.76
Chronic airway obstruction, unspecified	8*	13 356	407.93	8389	256.22
Chronic obstructive pulmonary disease with (acute) exacerbation	5	1464	44.71	455	13.9
Emphysema	7*	1909	58.31	1118	34.15
Hypoxemia	3	2364	72.2	1067	32.59
Other disease of nasal cavity and sinuses	2	5425	165.69	2601	79.44
Other diseases of trachea and bronchus	2	295	9.01	109	3.33
Other disorders of lung	8*	7555	230.75	3818	116.61
Other nonspecific abnormal finding of lung field	11*	14 800	452.03	6412	195.84
Other pulmonary insufficiency, not elsewhere classified	1	216	6.6	70	2.14
Other respiratory abnormalities, disorders, or diseases	3	12 605	384.99	5825	177.91
Pneumonitis due to inhalation of food or vomitus	3	365	11.15	123	3.76
Pulmonary congestion	2	846	25.84	181	5.53
Pulmonary eosinophilia	6	1353	41.32	348	10.63
Pulmonary fibrosis	12*	2237	68.32	1183	36.13
Unspecified pleural effusion	3	1978	60.41	875	26.72
Cardiovascular conditions					
Cardiomegaly	2	1524	46.55	889	27.15
Heart failure	4*	4923	150.36	3438	105.01
Hypotension, unspecified	2	815	24.89	300	9.16
Hematologic/immune disorders					
Leukocytopenia, unspecified	2	898	27.43	441	13.47
Leukocytosis, unspecified	5	3171	96.85	1700	51.92
Neutropenia, unspecified	1	97	2.96	46	1.4
Other pancytopenia	1	35	1.07	14	0.43
Other specified disease of white blood cells	3	616	18.81	241	7.36
Thrombocytopenia, unspecified	2	1046	31.95	666	20.34
Metabolic/systemic disorders					
Acute kidney failure, or renal failure, unspecified	6*	2120	64.75	1397	42.67
Electrolyte and fluid disorders	5	17 216	525.82	6847	209.13
Febrile convulsions	4	893	27.27	310	9.47
Rhabdomyolysis	1	105	3.21	43	1.31
Medication-related					
Adverse drug event	1	1403	42.85	657	20.07
Other					
Oral aphthae	1	422	12.89	129	3.94
Sialadenitis	2	562	17.16	312	9.53

See Supplementary Appendix Figures G and H for a summary of the DiD results for influenza case versus control weekly interaction effects. Conditions where the number of weeks elevated as noted by a “*” were deemed to return to a new elevated baseline following influenza. In such cases, the CUSUM change-point approach was used to identify the number of weeks for which the condition was elevated.

^a^Incidence among cases and controls was computed as the number of cases with the event during the period of elevated activity (i.e., number of weeks elevated, noted in column 1) per 100 K enrollees.

## DISCUSSION

Analyzing a study cohort of over 3 million influenza cases and matched controls, we found that the frequency of 67 diagnostic categories was elevated for at least 1 week after an influenza diagnosis. While many of these diagnostic categories are classically associated with influenza (eg, fevers and cough), we also found an increased incidence of visits associated with diagnostic categories not commonly considered or prioritized (eg, fluid and electrolyte disorders, and malaise and fatigue). In addition, many conditions were elevated for long periods: 21 were elevated for at least 5 weeks. Our findings have important implications for estimating not only the burden of illness attributable to influenza but also informing future studies (eg, length of follow-up, healthcare utilization and costs associated with postacute sequelae of influenza) designed to prevent or treat influenza.

Influenza symptoms [21–23] and secondary conditions associated with influenza have been reviewed elsewhere [24, 25]. However, prior studies have primarily focused on: improving the diagnosis of influenza [3, 21, 26–28], measuring influenza-related outcomes [29–31], or influenza-related disease burden [24, 25]. Also, while diagnostic-related studies focus on symptoms at the time of diagnosis [3, 26, 28], the manifestations of influenza may change throughout the course of illness as secondary bacterial infections and other postacute sequel of influenza develop. Thus, studies exclusively focusing on early symptoms may not capture the full range of symptoms and conditions attributable to influenza. In addition, studies of postacute sequelae of influenza may depend upon the age of the participants under study.

Beyond improving the diagnosis, other investigations of influenza symptoms involve identifying potential treatment outcomes. For example, patient-reported-outcome measures [29] have been developed and validated [29, 31]. Unfortunately, most of this work has focused on short time periods. While some studies report that symptoms might last longer than 2 weeks [3, 5], longer durations have not been extensively explored beyond focused studies of narrow outcomes (eg, cough [32]). Indeed, prior to COVID-19, respiratory-infection studies did not focus on long-term outcomes [33]. In addition, prior studies typically focus on a limited number of signs, symptoms or conditions. Clinical trials investigating influenza-related outcomes commonly focus on very a limited set of symptoms (eg, fever muscle/joint pain, fatigue, chills/sweating, nasal symptoms, core throat, and cough) [34, 35].

Our exploratory approach systematically evaluated patterns of healthcare utilization for the 2000 most commonly occurring diagnosis codes over a 1-year period following an influenza event. As a result, we identified diagnostic conditions that may not have been previously considered while also exploring longer time periods. Although many of the diagnostic conditions we identified were respiratory-infection related, our list is more expansive than outcomes typically used for influenza treatment trials [34, 35]. An additional advantage to our approach is that we control for health-seeking behavior through our DiD analysis. (eg, the control population had lower baseline healthcare utilization than our influenza-case population). Our DiD approach allows us to not only identify which conditions were elevated relative to a control population but also compared to the baseline level of care that was observed prior to the influenza diagnosis. Thus, the effects we detect should be robust to factors such as healthcare-seeking behavior that may differ between influenza cases and controls.

There are several limitations associated with our work. First, we used administrative data and did not have specific laboratory results, imaging or notes to validate the diagnostic codes we considered, and some cases of influenza might have been misclassified. Individuals included in the analyses had to have continuous healthcare coverage in the year before and after the influenza event. Thus, some individuals may be excluded by construction (eg, sicker patients who died because of influenza or individuals whose health plans changed due to life events such as childbearing). Second, our data set only included people with private insurance or supplemental Medicare coverage. Our results may not be generalizable to people without private insurance, who may have different health-seeking behaviors, comorbidities or health literacy. Our results may also not be generalizable to people in countries outside the United States. Third, we did not directly evaluate the effects of patient comorbidities, influenza-vaccination status, or antiviral prescriptions. Fourth, many people do not seek care for influenza [36] and many cases presenting to healthcare visits are not diagnosed [37, 38]. In addition, many cases of influenza are asymptomatic and some of these cases may be associated with secondary diseases and conditions [1]. Our approach does not consider such cases. Finally, some of our control patients may have had undiagnosed influenza or other respiratory viruses. This could dilute the observed effect, potentially underestimating the true impact of influenza on postinfection outcomes.

Many of the limitations could be addressed by replicating our analysis in other settings, populations, and using other data sources, such as those that include laboratory results as well as vaccination status. In addition, future work could also help extend our findings to questions not directly addressed. Specifically, future work could track the incidence of different symptoms and conditions according to different types of presentations (eg, complicated or uncomplicated influenza), compare cases presenting with influenza and pneumonia to just those presenting with influenza, and consider baseline comorbidities. Indeed, the number and duration of different conditions associated with influenza may differ based on the severity of disease at presentation. Interestingly, in the case of long COVID, prolonged symptoms occurred in both severe and mild cases [39, 40]. In addition, future work could differentiate between different stages of symptoms or attempt to categorize different stages of symptoms.

Despite the limitations, we provide additional evidence for the existence of acute post-influenza sequelae in a subset of patients suffering from influenza. Our results provide a compelling rationale for not only considering a broader range of signs, symptoms and diseases associated with influenza, but also measuring outcomes over a longer follow-up period. Failure to account for the duration of a broader set of symptoms will lead to underestimates of the burden of influenza. Finally, our results have important implications for designing studies to prevent or treat influenza regarding the duration of follow-up and what outcomes to measure after interventions.

## Supplementary Material

ofag629_Supplementary_Data

## Supplementary Data

Supplementary materials are available at *Open Forum Infectious Diseases* online. Consisting of data provided by the authors to benefit the reader, the posted materials are not copyedited and are the sole responsibility of the authors, so questions or comments should be addressed to the corresponding author.

## References

[1] Leung NH, Xu C, Ip DK, Cowling BJ. Review article: the fraction of influenza virus infections that are asymptomatic: a systematic review and meta-analysis. Epidemiology 2015; 26:862–72. 10.1097/EDE.0000000000000340 PMC4586318 26133025

[2] Kalil AC, Thomas PG. Influenza virus-related critical illness: pathophysiology and epidemiology. Crit Care 2019; 23:258. 10.1186/s13054-019-2539-x PMC6642581 31324202

[3] Eccles R . Understanding the symptoms of the common cold and influenza. Lancet Infect Dis 2005; 5:718–25. 10.1016/S1473-3099(05)70270-X PMC7185637 16253889

[4] Paules C, Subbarao K. Influenza. Lancet 2017; 390:697–708. 10.1016/S0140-6736(17)30129-0 28302313

[5] Lee CS, Lee JH. Dynamics of clinical symptoms in patients with pandemic influenza A (H1N1). Clin Microbiol Infect 2010; 16:389–90. 10.1111/j.1469-0691.2010.03117.x 20222893

[6] Okahashi N, Sumitomo T, Nakata M, Kawabata S. Secondary streptococcal infection following influenza. Microbiol Immunol 2022; 66:253–63. 10.1111/1348-0421.12965 35088451

[7] Jia L, Xie J, Zhao J, et al Mechanisms of severe mortality-associated bacterial co-infections following influenza virus infection. Front Cell Infect Microbiol 2017; 7:338. 10.3389/fcimb.2017.00338 PMC5540941 28824877

[8] Short KR, Diavatopoulos DA, Thornton R, et al Influenza virus induces bacterial and nonbacterial otitis media. J Infect Dis 2011; 204:1857–65. 10.1093/infdis/jir618 PMC3209809 21930608

[9] Short KR, Reading PC, Brown LE, et al Influenza-induced inflammation drives pneumococcal otitis media. Infect Immun 2013; 81:645–52. 10.1128/IAI.01278-12 PMC3584865 23319557

[10] Chonmaitree T, Revai K, Grady JJ, et al Viral upper respiratory tract infection and otitis media complication in young children. Clin Infect Dis 2008; 46:815–23. 10.1086/528685 PMC2744371 18279042

[11] Henderson FW, Collier AM, Sanyal MA, et al A longitudinal study of respiratory viruses and bacteria in the etiology of acute otitis media with effusion. N Engl J Med 1982; 306:1377–83. 10.1056/NEJM198206103062301 6281639

[12] Meiring S, Tempia S, Dominic EM, et al Excess invasive meningococcal disease associated with seasonal influenza, South Africa, 2003–2018. Clin Infect Dis 2022; 74:1729–35. 10.1093/cid/ciab702 PMC9155629 34389845

[13] Salomon A, Berry I, Tuite AR, et al Influenza increases invasive meningococcal disease risk in temperate countries. Clin Microbiol Infect 2020; 26:1257.e1–.e7. 10.1016/j.cmi.2020.01.004 31935565

[14] Miller AC, Singh I, Koehler E, Polgreen PM. A smartphone-driven thermometer application for real-time population- and individual-level influenza surveillance. Clin Infect Dis 2018; 67:388–97. 10.1093/cid/ciy073 29432526

[15] Behrouzi B, Udell JA. Moving the needle on atherosclerotic cardiovascular disease and heart failure with influenza vaccination. Curr Atheroscler Rep 2021; 23:78. 10.1007/s11883-021-00973-w PMC8528654 34671861

[16] Bao W, Li Y, Wang T, et al Effects of influenza vaccination on clinical outcomes of chronic obstructive pulmonary disease: a systematic review and meta-analysis. Ageing Res Rev 2021; 68:101337. 10.1016/j.arr.2021.101337 33813014

[17] Bekkat-Berkani R, Wilkinson T, Buchy P, et al Seasonal influenza vaccination in patients with COPD: a systematic literature review. BMC Pulm Med 2017; 17:79. 10.1186/s12890-017-0420-8 PMC5415833 28468650

[18] Gerke AK, Yang M, Tang F, Foster ED, Cavanaugh JE, Polgreen PM. Association of hospitalizations for asthma with seasonal and pandemic influenza. Respirology (Carlton, VIC) 2014; 19:116–21. 10.1111/resp.12165 PMC3877191 23931674

[19] Foster ED, Cavanaugh JE, Haynes WG, et al Acute myocardial infarctions, strokes and influenza: seasonal and pandemic effects. Epidemiol Infect 2013; 141:735–44. 10.1017/S0950268812002890 PMC3733340 23286343

[20] Chow EJ, Rolfes MA, O’Halloran A, et al Respiratory and nonrespiratory diagnoses associated with influenza in hospitalized adults. JAMA Netw Open 2020; 3:e201323–201323. 10.1001/jamanetworkopen.2020.1323 PMC7084169 32196103

[21] Monto AS, Gravenstein S, Elliott M, Colopy M, Schweinle J. Clinical signs and symptoms predicting influenza infection. Arch Intern Med 2000; 160:3243–7. 10.1001/archinte.160.21.3243 11088084

[22] Call SA, Vollenweider MA, Hornung CA, Simel DL, McKinney WP. Does this patient have influenza? JAMA 2005; 293:987–97. 10.1001/jama.293.8.987 15728170

[23] Ebell MH, White LL, Casault T. A systematic review of the history and physical examination to diagnose influenza. J Am Board Fam Pract 2004; 17:1–5. 10.3122/jabfm.17.1.1 15014046

[24] Sellers SA, Hagan RS, Hayden FG, Fischer WA 2nd. The hidden burden of influenza: a review of the extra-pulmonary complications of influenza infection. Influenza Other Respir Viruses 2017; 11:372–93. 10.1111/irv.12470 PMC5596521 28745014

[25] Macias AE, McElhaney JE, Chaves SS, et al The disease burden of influenza beyond respiratory illness. Vaccine 2021; 39 Suppl 1:A6–A14. 10.1016/j.vaccine.2020.09.048 PMC7545338 33041103

[26] Van Elden LJ, van Essen GA, Boucher CA, et al Clinical diagnosis of influenza virus infection: evaluation of diagnostic tools in general practice. Br J Gen Pract 2001; 51:630–4. PMC1314072 11510391

[27] Michiels B, Thomas I, Van Royen P, Coenen S. Clinical prediction rules combining signs, symptoms and epidemiological context to distinguish influenza from influenza-like illnesses in primary care: a cross sectional study. BMC Fam Pract 2011; 12:4. 10.1186/1471-2296-12-4 PMC3045895 21306610

[28] Shah SC, Rumoro DP, Hallock MM, et al Clinical predictors for laboratory-confirmed influenza infections: exploring case definitions for influenza-like illness. Infect Control Hosp Epidemiol 2015; 36:241–8. 10.1017/ice.2014.64 25695163

[29] Powers JH, Guerrero ML, Leidy NK, et al Development of the flu-PRO: a patient-reported outcome (PRO) instrument to evaluate symptoms of influenza. BMC Infect Dis 2015; 16:1–11. 10.1186/s12879-015-1330-0 PMC4700740 26729246

[30] Han A, Poon J-L, Powers JH, Leidy NK, Yu R, Memoli MJ. Using the influenza patient-reported outcome (FLU-PRO) diary to evaluate symptoms of influenza viral infection in a healthy human challenge model. BMC Infect Dis 2018; 18:353. 10.1186/s12879-018-3220-8 PMC6064178 30055573

[31] Powers JH III, Bacci ED, Leidy NK, et al Performance of the inFLUenza patient-reported outcome (FLU-PRO) diary in patients with influenza-like illness (ILI). PLoS One 2018; 13:e0194180. 10.1371/journal.pone.0194180 PMC5863969 29566007

[32] Lin L, Yang ZF, Zhan YQ, et al The duration of cough in patients with H1N1 influenza. Clin Respir J 2017; 11:733–8. 10.1111/crj.12409 PMC7162306 26519198

[33] Chong EH, Wee IJY, Low JGH, Tambyah PA, Ng DHL. Post-acute sequelae of non-COVID-19 respiratory viral infections: a systematic review. Respirology (Carlton, VIC) 2026; doi:10.1002/resp.70302 42616003

[34] Ikematsu H, Kawai N, Iwaki N, et al Duration of fever and other symptoms after the inhalation of laninamivir octanoate hydrate in the 2016/17 Japanese influenza season; comparison with the 2011/12 to 2015/16 seasons. J Infect Chemother 2018; 24:718–24. 10.1016/j.jiac.2018.04.013 29861186

[35] Ison MG, Portsmouth S, Yoshida Y, et al Early treatment with baloxavir marboxil in high-risk adolescent and adult outpatients with uncomplicated influenza (CAPSTONE-2): a randomised, placebo-controlled, phase 3 trial. Lancet Infect Dis 2020; 20:1204–14. 10.1016/S1473-3099(20)30004-9 32526195

[36] Carrat F, Sahler C, Rogez S, et al Influenza burden of illness: estimates from a national prospective survey of household contacts in France. Arch Intern Med 2002; 162:1842–8. 10.1001/archinte.162.16.1842 12196082

[37] Hartman L, Zhu Y, Edwards KM, Griffin MR, Talbot HK. Underdiagnosis of influenza virus infection in hospitalized older adults. J Am Geriatr Soc 2018; 66:467–72. 10.1111/jgs.15298 PMC5863754 29341100

[38] McCarthy Z, Athar S, Alavinejad M, et al Quantifying the annual incidence and underestimation of seasonal influenza: a modelling approach. Theor Biol Med Model 2020; 17:11. 10.1186/s12976-020-00129-4 PMC7347407 32646444

[39] O'Mahoney LL, Routen A, Gillies C, et al The prevalence and long-term health effects of long COVID among hospitalised and non-hospitalised populations: a systematic review and meta-analysis. EClinicalMedicine 2023; 55:101762. 10.1016/j.eclinm.2022.101762 PMC9714474 36474804

[40] O'Mahoney LL, Routen A, Gillies C, et al Corrigendum to “the prevalence and long-term health effects of long COVID among hospitalised and non-hospitalised populations: a systematic review and meta-analysis”. EClinicalMedicine 2023; 59:101959. 10.1016/j.eclinm.2023.101959 PMC10115131 37096187

