# Supplementary material for: Estimating the Range and Duration of Post-influenza Symptoms, Conditions, and Diseases
Source: Open Forum Infect Dis. 2026 Oct 6;13(10):ofag629. doi: 10.1093/ofid/ofag629 (PMC13660689; doi:10.1093/ofid/ofag629)
Supplement: Supplementary file 1 [file ofag629_supplementary_data.zip › Appendix_Figure_H.pdf]

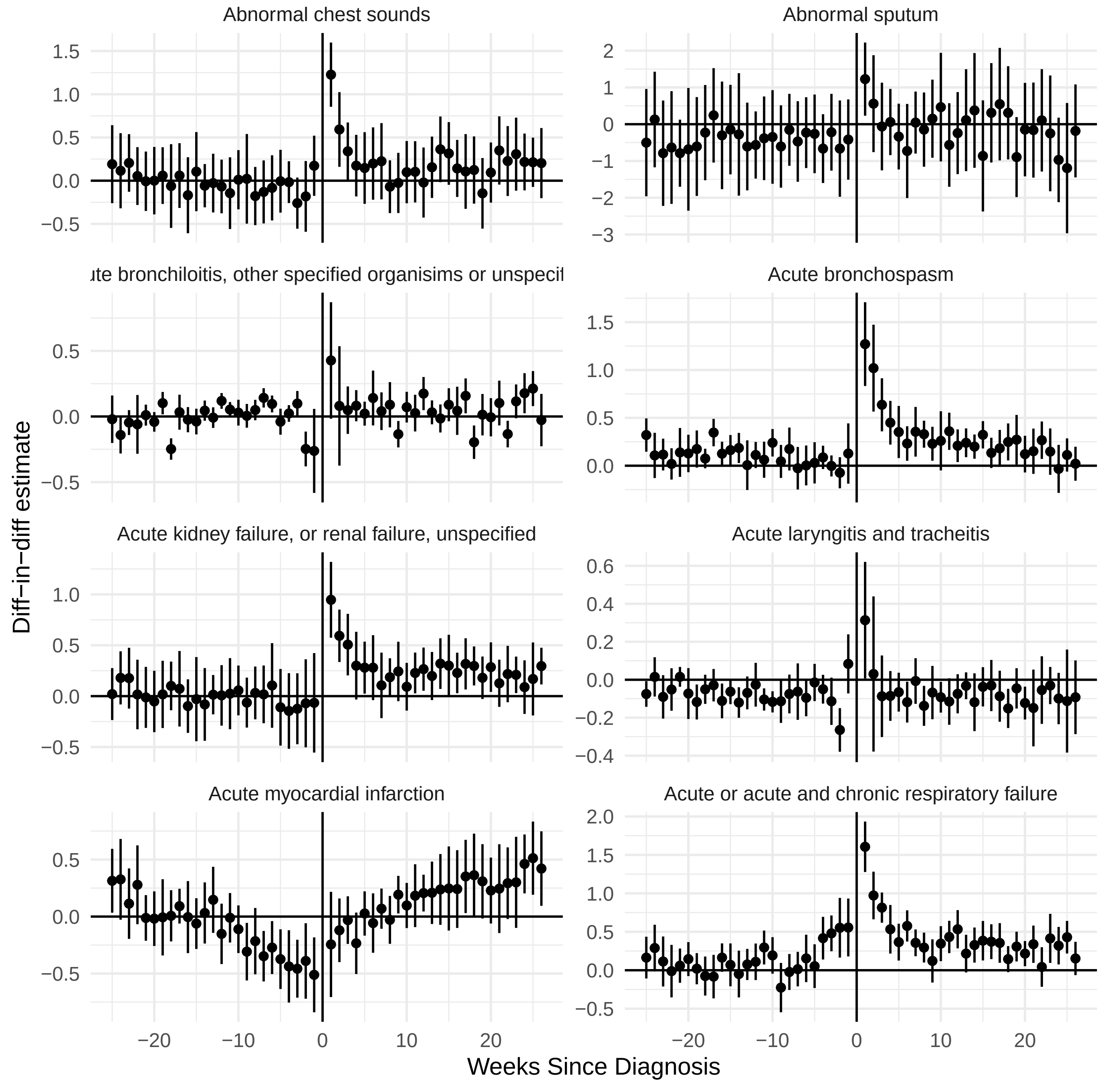

Abnormal chest sounds
Abnormal sputum
ute bronchiloitis, other specified organisims or unspecif
Acute bronchospasm
Acute kidney failure, or renal failure, unspecified
Acute laryngitis and tracheitis
Acute myocardial infarction
Acute or acute and chronic respiratory failure
Diff−in−diff estimate
Weeks Since Diagnosis

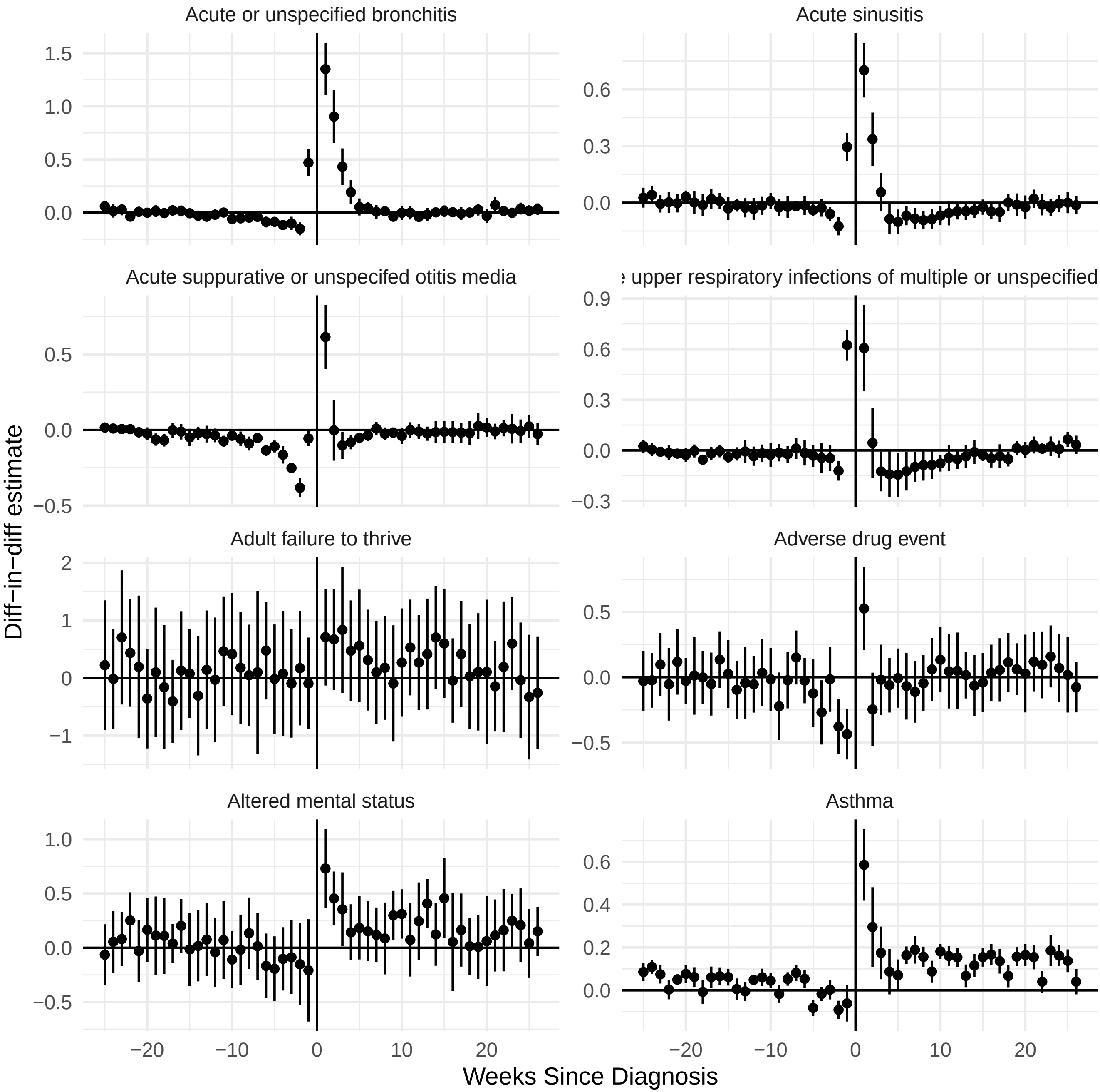

Acute or unspecified bronchitis
Acute sinusitis
Acute suppurative or unspecifed otitis media
e upper respiratory infections of multiple or unspecified
Adult failure to thrive
Adverse drug event
Altered mental status
Asthma
Diff−in−diff estimate
Weeks Since Diagnosis

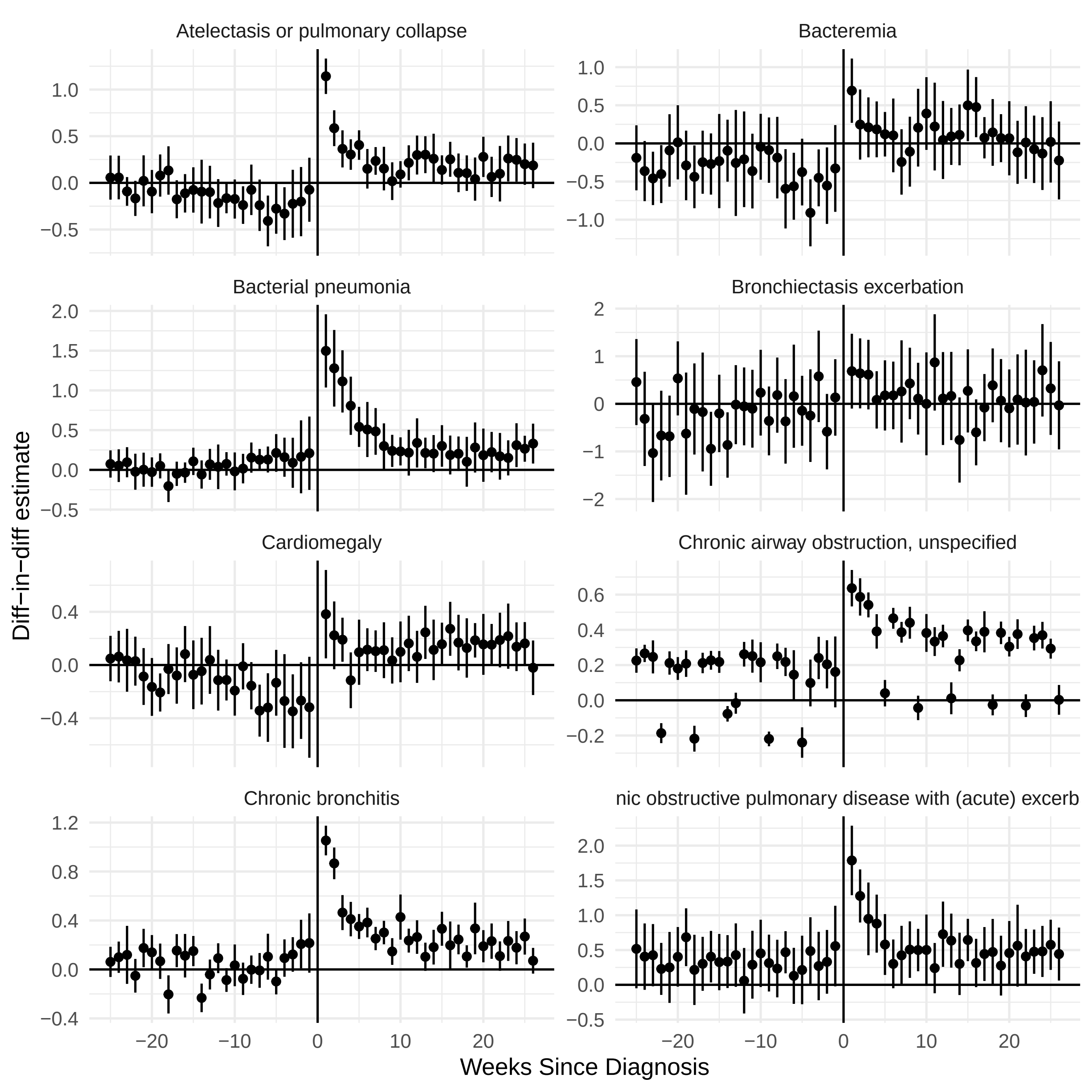
Atelectasis or pulmonary collapse
Bacteremia
Bacterial pneumonia
Bronchiectasis excerbation
Cardiomegaly
Chronic airway obstruction, unspecified
Chronic bronchitis
nic obstructive pulmonary disease with (acute) excerb
Diff−in−diff estimate
Weeks Since Diagnosis

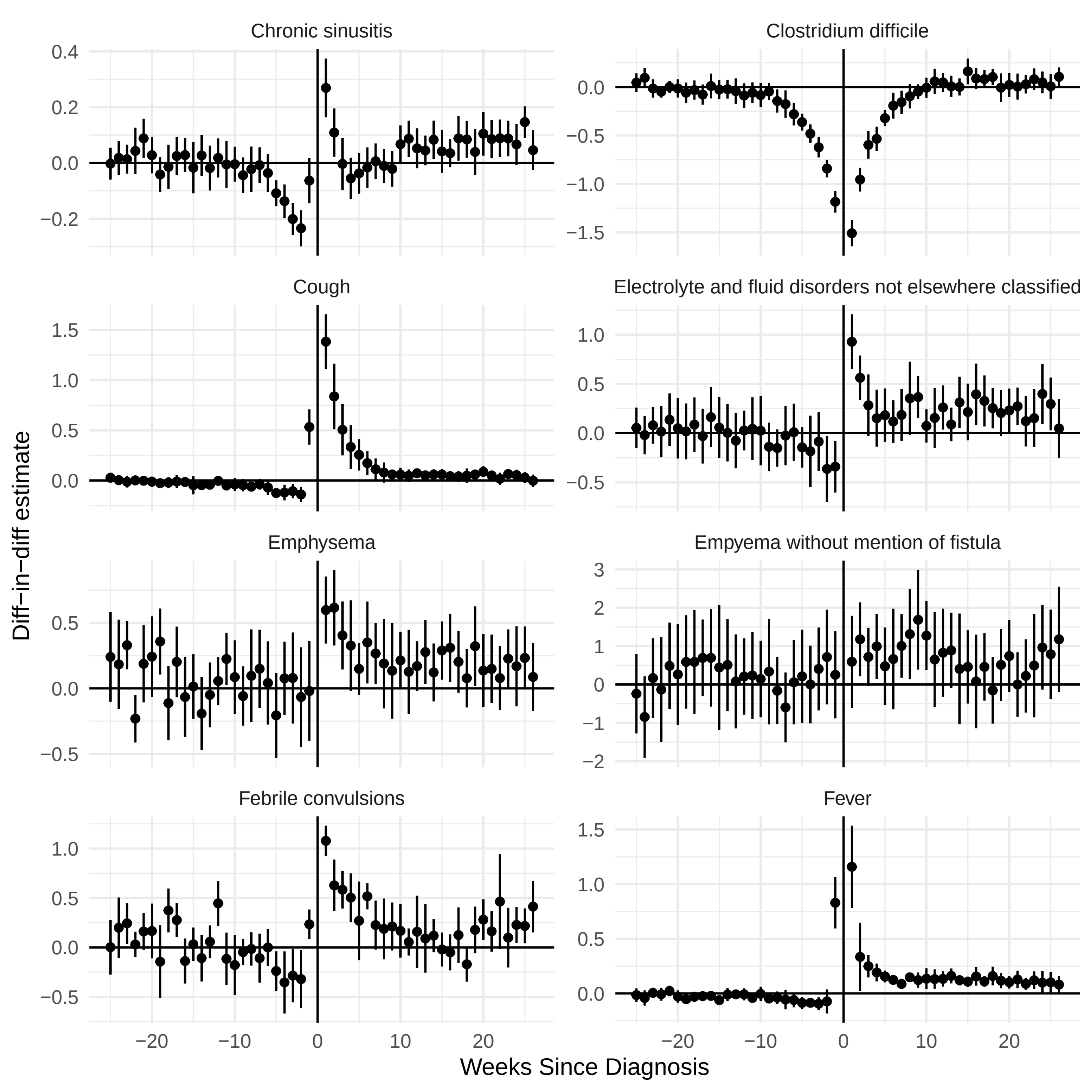

Chronic sinusitis
Clostridium difficile
Cough
Electrolyte and fluid disorders not elsewhere classified
Emphysema
Empyema without mention of fistula
Febrile convulsions
Fever
Diff−in−diff estimate
Weeks Since Diagnosis
−20
−10
0
10
20

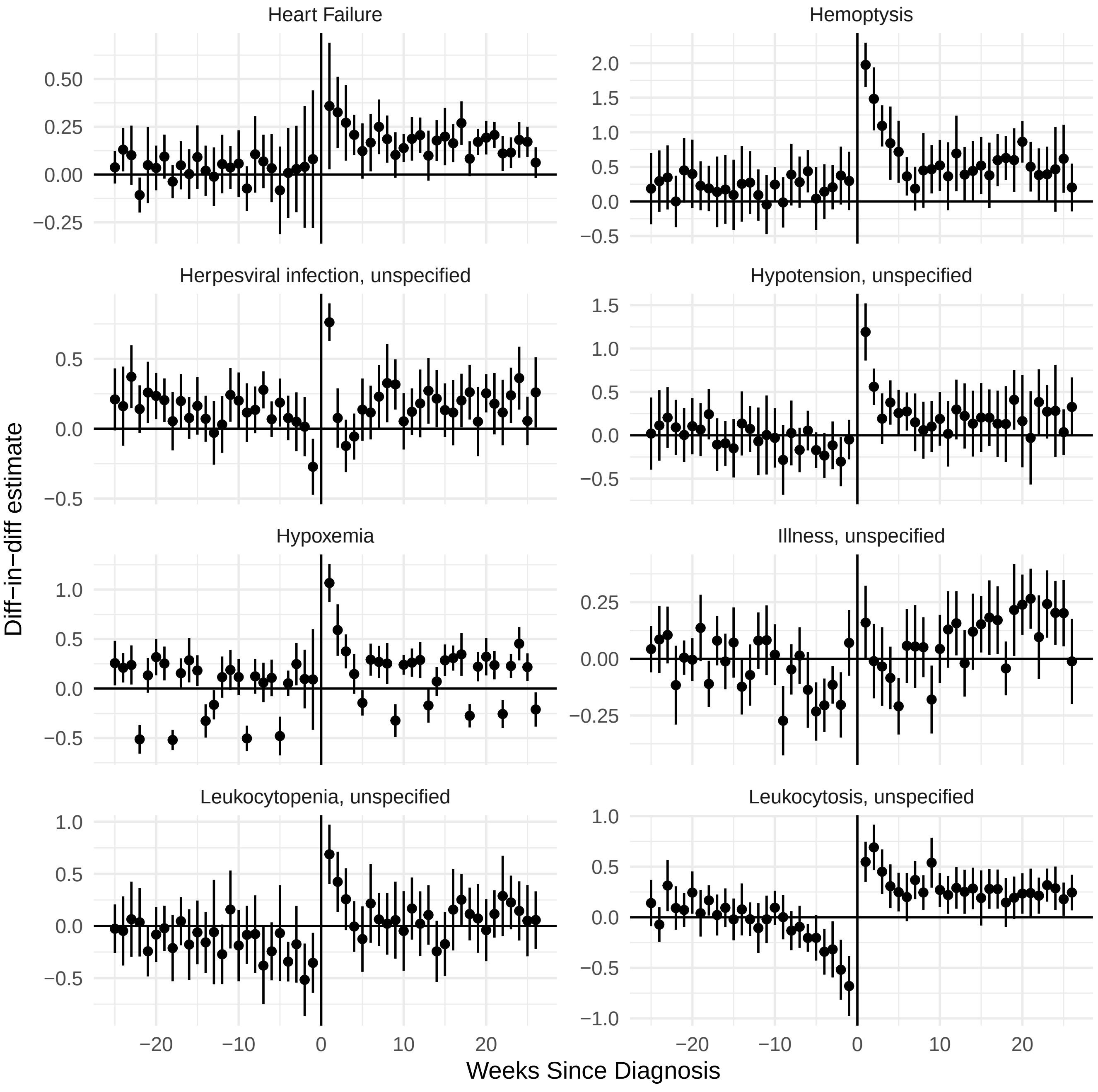
Heart Failure
Hemoptysis
Herpesviral infection, unspecified
Hypotension, unspecified
Hypoxemia
Illness, unspecified
Leukocytopenia, unspecified
Leukocytosis, unspecified
Diff−in−diff estimate
Weeks Since Diagnosis
0.50
0.25
0.00
−0.25
2.0
1.5
1.0
0.5
0.0
−0.5
−1.0
−20
−10
0
10
20

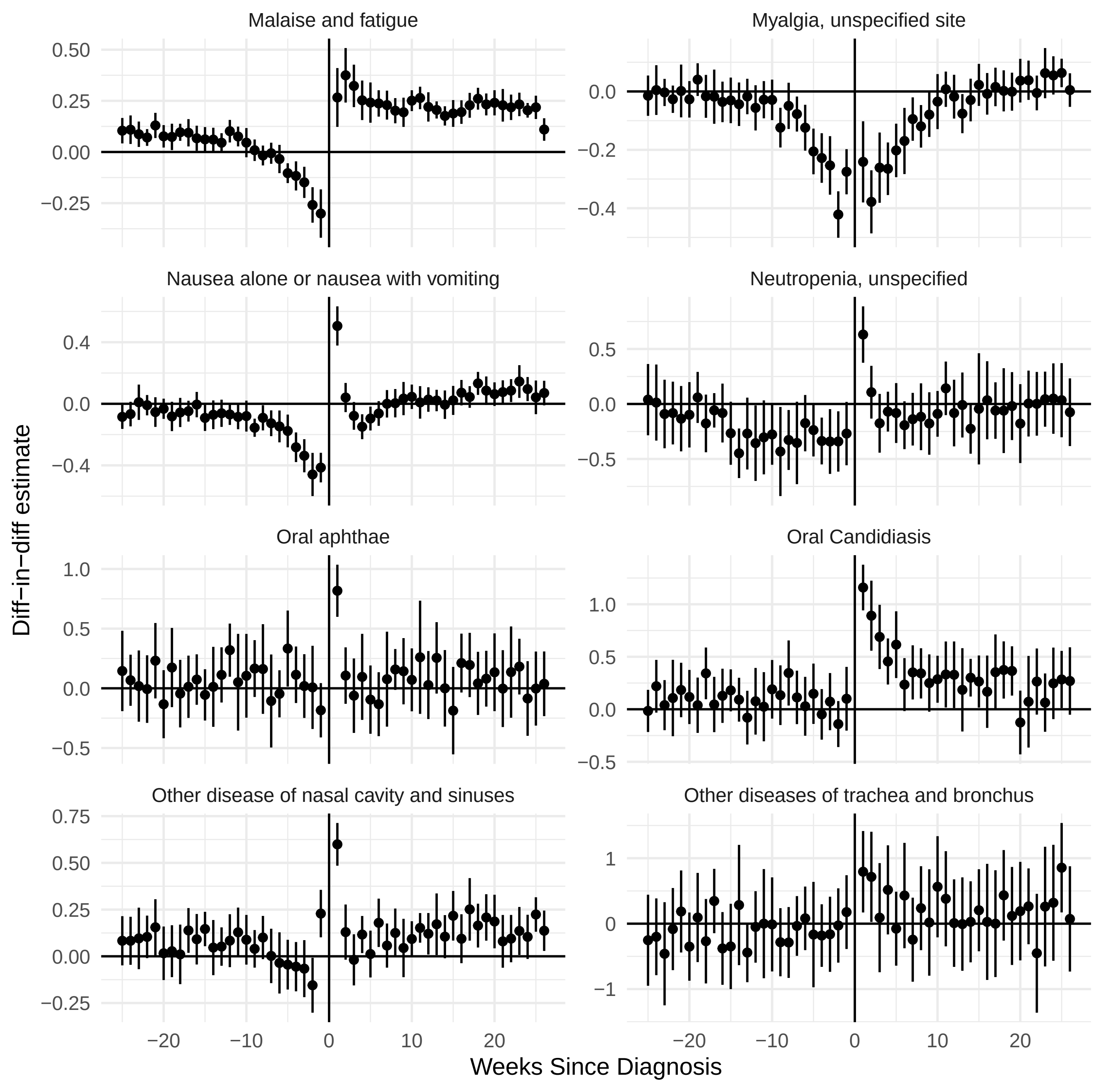
Malaise and fatigue
Myalgia, unspecified site
Nausea alone or nausea with vomiting
Neutropenia, unspecified
Oral aphthae
Oral Candidiasis
Other disease of nasal cavity and sinuses
Other diseases of trachea and bronchus
Diff−in−diff estimate
Weeks Since Diagnosis
−20
−10
0
10
20

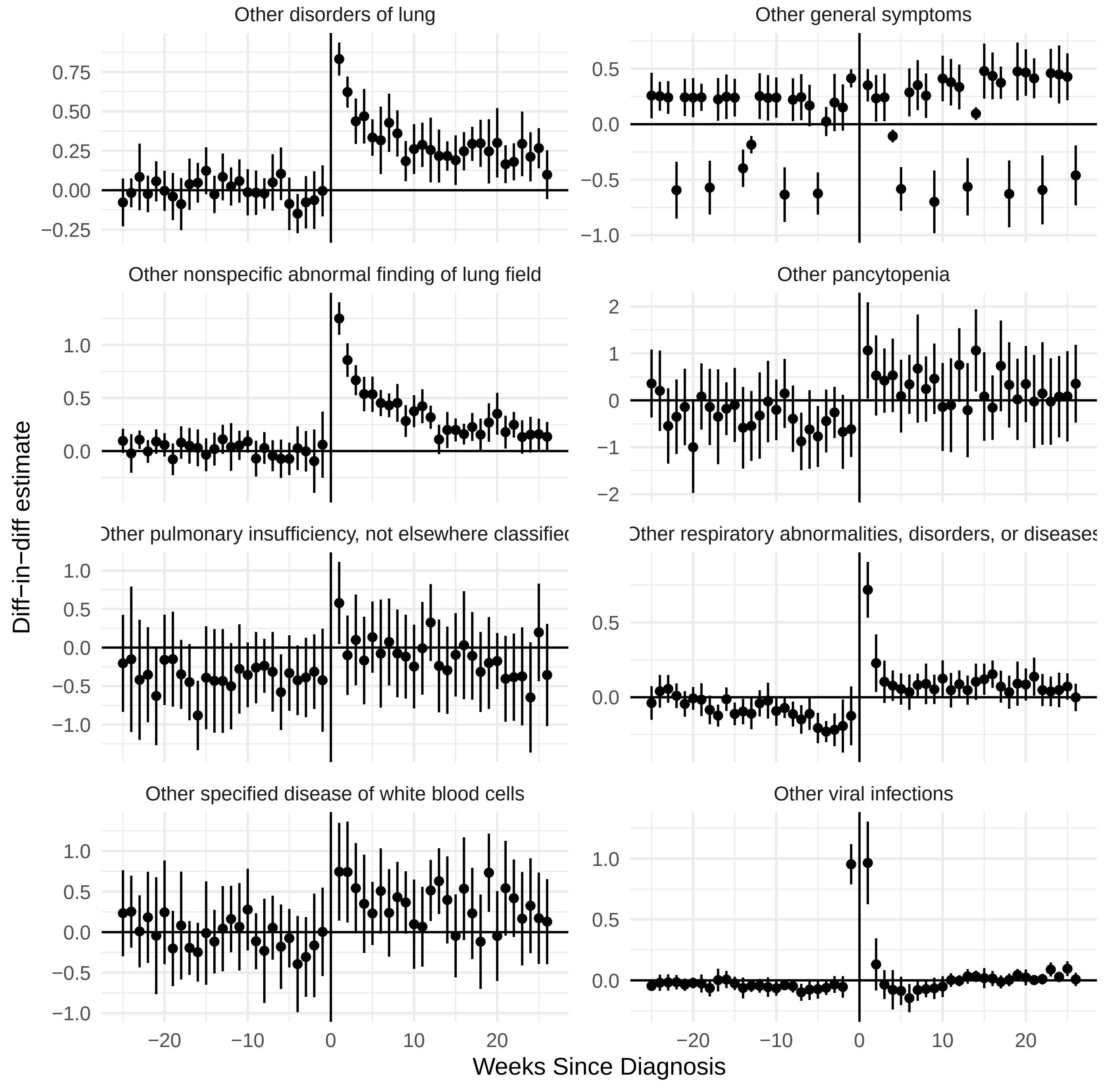

Other disorders of lung
Other general symptoms
Other nonspecific abnormal finding of lung field
Other pancytopenia
Other pulmonary insufficiency, not elsewhere classified
Other respiratory abnormalities, disorders, or diseases
Other specified disease of white blood cells
Other viral infections
Diff−in−diff estimate
Weeks Since Diagnosis

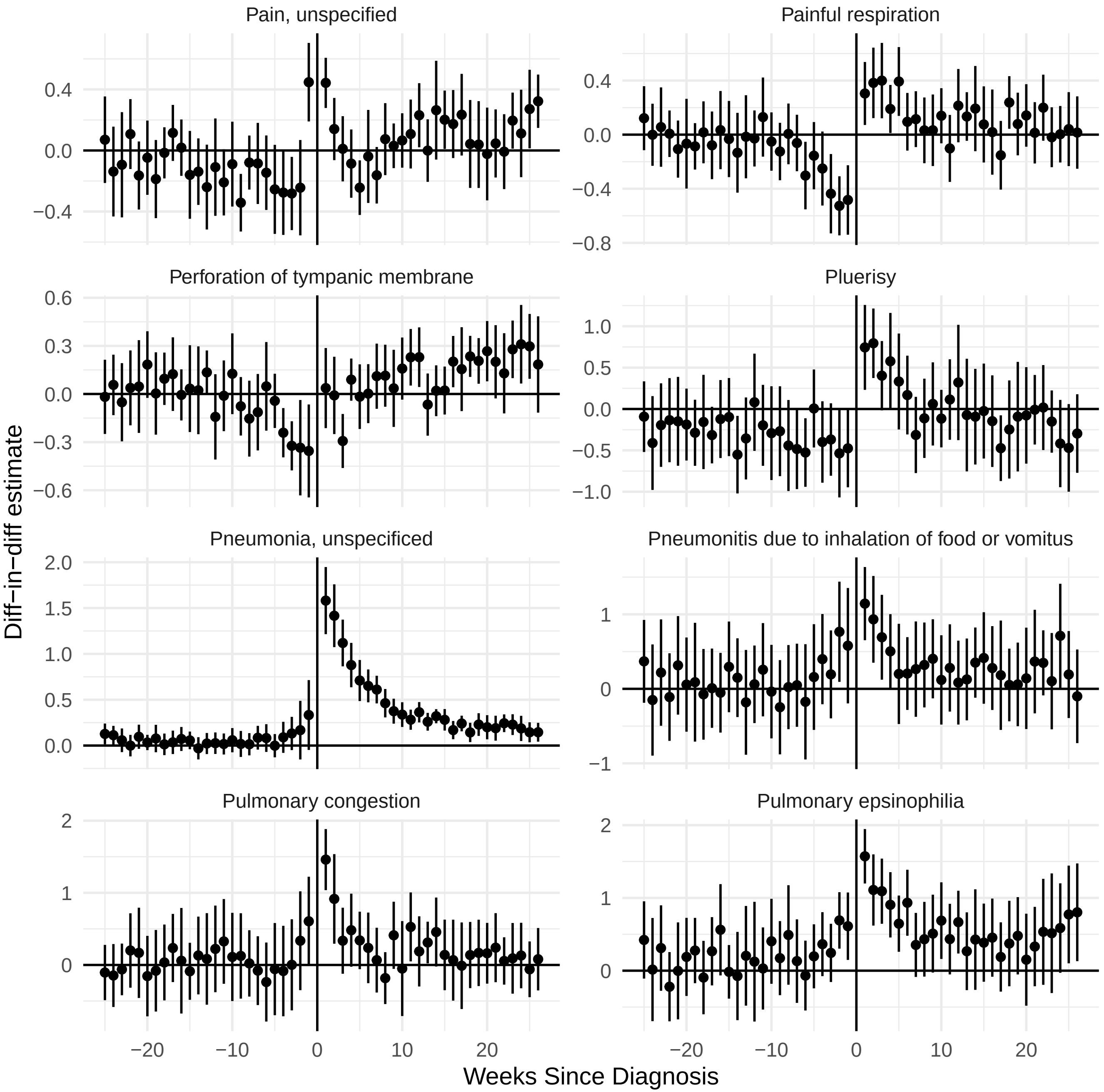

Pain, unspecified
Painful respiration
Perforation of tympanic membrane
Pluerisy
Pneumonia, unspecificed
Pneumonitis due to inhalation of food or vomitus
Pulmonary congestion
Pulmonary epsinophilia
Diff−in−diff estimate
Weeks Since Diagnosis

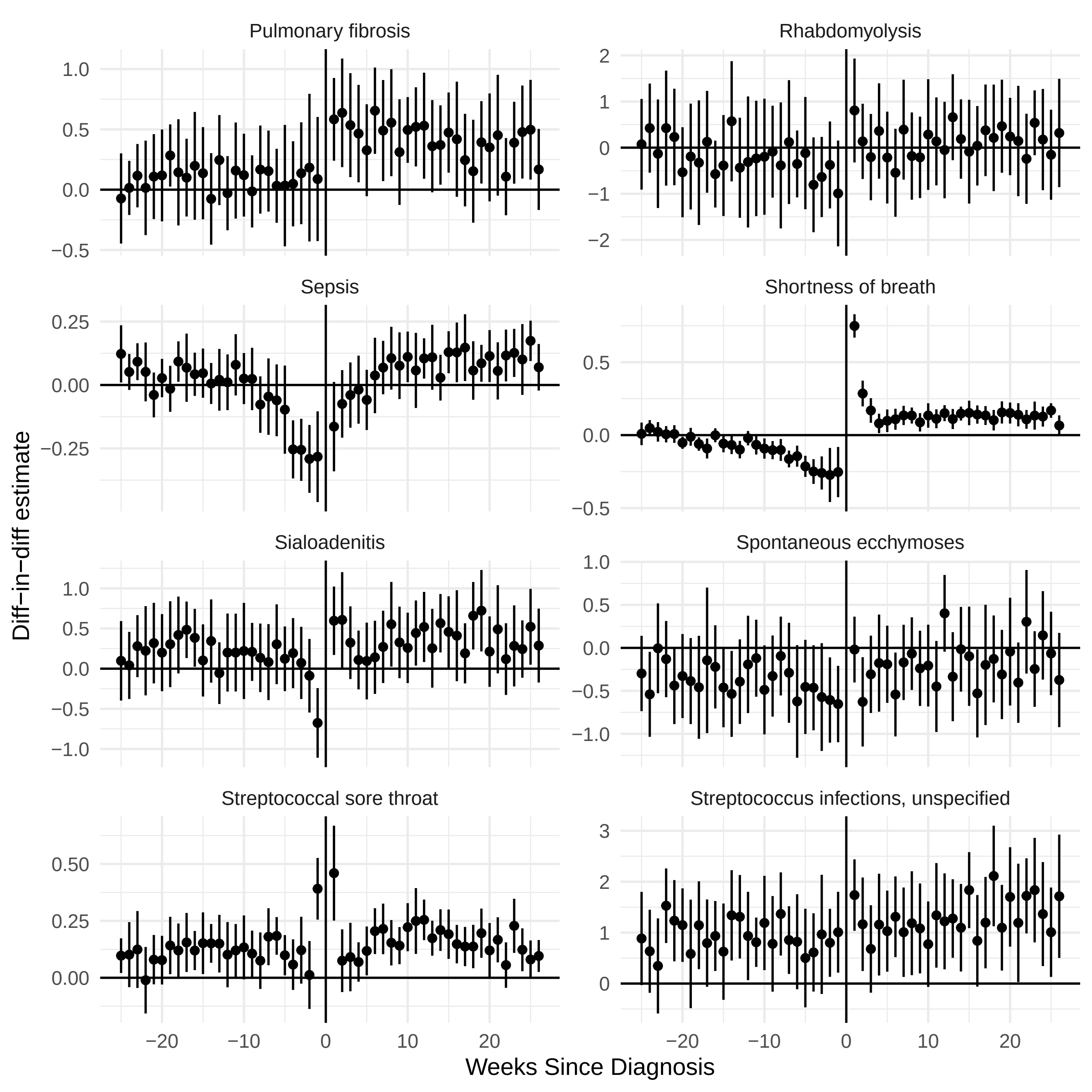
Pulmonary fibrosis
Rhabdomyolysis
Sepsis
Shortness of breath
Sialoadenitis
Spontaneous ecchymoses
Streptococcal sore throat
Streptococcus infections, unspecified
Diff−in−diff estimate
Weeks Since Diagnosis

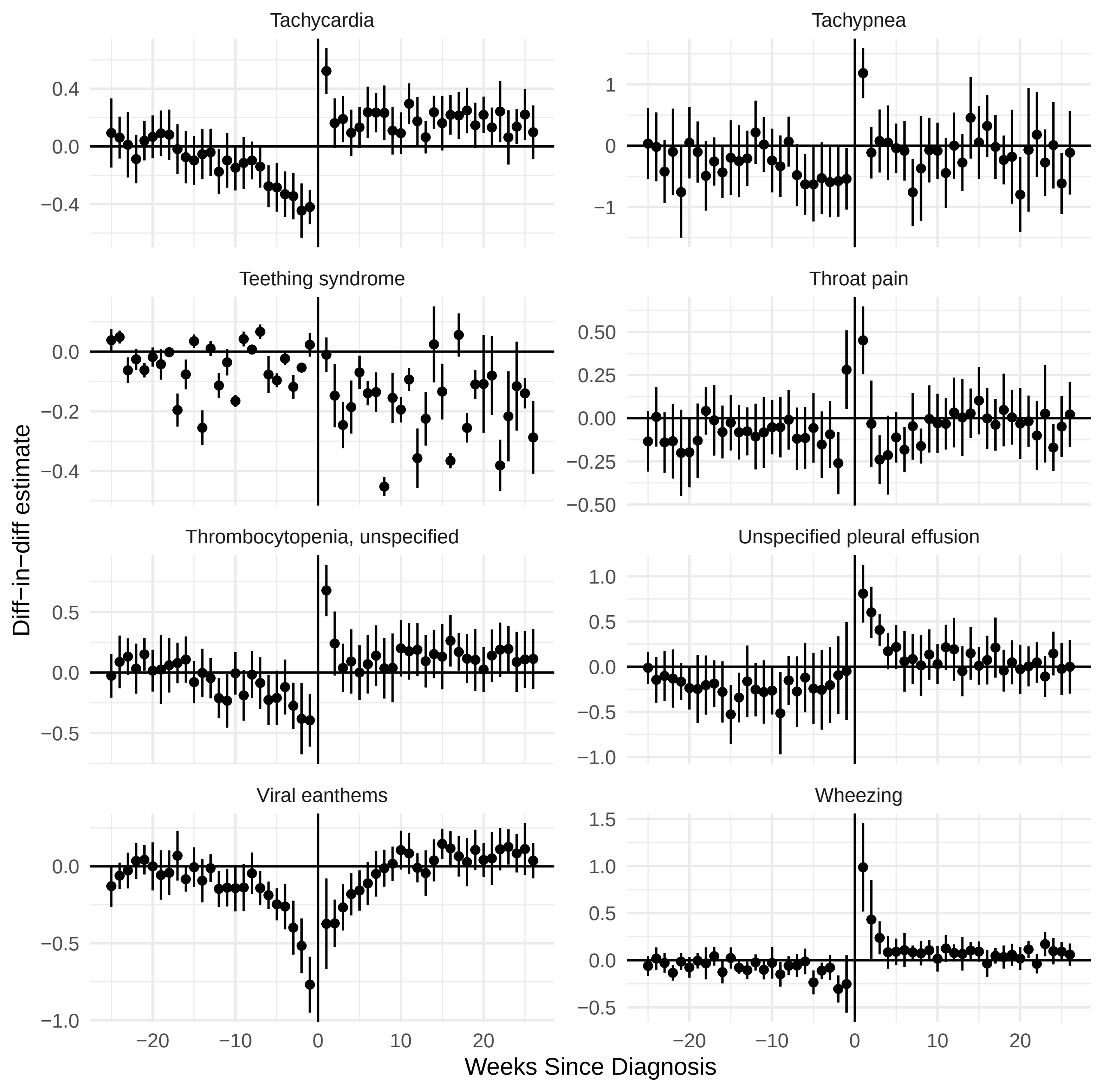
Tachycardia
Tachypnea
Teething syndrome
Throat pain
Thrombocytopenia, unspecified
Unspecified pleural effusion
Viral eanthems
Wheezing
Diff−in−diff estimate
Weeks Since Diagnosis
