# Supplementary material for: Estimating the Range and Duration of Post-influenza Symptoms, Conditions, and Diseases
Source: Open Forum Infect Dis. 2026 Oct 6;13(10):ofag629. doi: 10.1093/ofid/ofag629 (PMC13660689; doi:10.1093/ofid/ofag629)
Supplement: Supplementary file 1 [file ofag629_supplementary_data.zip › Appendix_Revised_Final.docx]

**APPENDIX MATERIAL**

**Appendix Methods**

**Appendix Methods 1 – *Stage 1: Cluster approach for identification of potential post-acute sequelae***

We utilized the clustering approach outlined in Miller *et al.* (2023) to identify clusters of healthcare visits that exhibited a trend consistent with potential post-acute sequelae following influenza.^20^ Specifically, for the 2,000 most frequently occurring ICD-9 and 2,000 most frequently occurring ICD-10 codes following an influenza diagnosis, we computed the trend in the proportion of healthcare visits each day before or after the index event that involved the given diagnosis for all case patients. Next, we normalized each daily frequency value between 0-1 by subtracting the minimum daily frequency and dividing by the range in daily frequency. We then compute a smoothed trend across all daily frequencies using a LOESS (locally estimated scatterplot smoothing) model. For each condition, this resulted in 730 values, one for each day before and after the index diagnosis (365 before and 365 after index). These values were then used to cluster ICD-9/10 codes into clusters of conditions that exhibited similar trends before and after the index event. We used K-means clustering and considered values for *k* from 3 to 30.

We identified *focal clusters* of interest based on two approaches. First, we used a single focal condition of “cough” (ICD-9 786.2 and ICD-10 R05) to identify all other conditions that occurred in a similar cluster. For this analysis, we computed the frequency with which a given condition clustered with cough across different values for the number of clusters *k* and ordered conditions by their frequency in this cough cluster. Second, we analyzed the mean shape of the resulting clusters identified, defined as the mean trend across conditions selected in a given cluster. We then identified those conditions which fell into clusters where the mean trend following influenza exhibited a pattern consistent with post-acute sequelae (i.e., generally elevated relative to the period prior to influenza). For this analysis, we restricted our attention to results involving a larger number of clusters (i.e., *k=30*).

**Appendix Methods 2 – *Stage 2: Difference-in-differences approach for estimating the increased frequency of visits following influenza*.**

Because follow-up care -- disease screening and exposure to healthcare environments in general -- can induce surveillance effects (e.g., screening for other diseases) or secondary healthcare-associated events (e.g., follow-up care, healthcare-associated infections), this phenomenon potentially creates the appearance of elevated healthcare patterns following a given visit/diagnosis. Thus, we consider a difference-in-differences (diff-in-diff) modelling approach to estimate the degree to which the frequency of a given condition is elevated in the weeks following influenza. Specifically, we evaluate the following basic model:

$$E\left( {No. Events}_{w,c,s,a,t} \right)=exp(\boldsymbol{\beta}({Week}_{w}:C{ase}_{c})+\gamma_{a}^{Age Group}{+ \gamma}_{s}^{Sex}+\gamma_{w}^{Week}+\gamma_{i}^{Case}+\gamma_{t}^{Index Month})$$

where $w,c,a, s,t$ are indices denoting the weeks since the index visit, the cohort, age group, sex and time of the index event date; the term $\boldsymbol{\beta}({Week}_{w}:C{ase}_{c})$ captures the interaction effects between weeks since index and case or control cohorts; and the various $\gamma$ values capture the fixed main effects of age-group, sex, week, case status and the timing of the index event. The primary effects of interest are the interaction terms between cases and controls, $\boldsymbol{\beta}\left( {Week}_{w}:C{ase}_{c} \right),$ particularly during the weeks following the index influenza event. Note, in this setup the fixed main effect for $\gamma_{i}^{Case}$ accounts for any underlying differences between the influenza case cohort and the matched control cohort (e.g., healthcare-seeking behavior) while $\gamma_{w}^{Week}$ accounts for any weeks-since-index effects that are consistent between cases and controls (e.g., surveillance or follow-up care patterns).

To estimate the above relationship for a given ICD code (or set of ICD codes), we aggregated counts of healthcare visits for the given code by the weeks since/before the index diagnosis, the cohort, and the strata that defined a matched cohort (i.e., age, sex, month and year of the index event). We included an offset term in the above model to account for the number of enrollees in a given stratum. Finally, we estimate the above relationship using a quasi-Poisson distribution and the *fixest* package (version 0.11.2) in the R programming language. We apply this model to each of the different conditions identified as potential post-acute sequalae for influenza, described in the prior section. After obtaining these estimates for each condition, we identified those conditions that have a consistently elevated trend post-influenza.

**Appendix Methods 3 – *Stage 3: Identification of time period following influenza in which conditions were elevated.***

After identifying diagnosis codes that demonstrated a healthcare visit pattern consistent with potential post-acute sequelae, we then reviewed all codes for clinical plausibility, aggregated codes into meaningful condition-specific categories, and estimated the length of time the condition appeared to be elevated following influenza.

First, each ICD code was reviewed independently by two physicians to determine if the code was “clinically plausible” as a post-acute sequelae of influenza. Any differences were reviewed by a third reviewer until a consensus was reached. Next, for all plausible remaining codes, ICD-9 and ICD-10 codes were aggregated into clinically meaningful categories by these same three reviewers.

Second, for each of the clinically meaningful categories, we recomputed weekly visit counts for the aggregated code set (across both ICD-9 and ICD-10 codes). We then re-ran the diff-in-diff model, described in stage 2, for each aggregated code set.

Third, we used the results of the aggregated diff-in-diff analysis to identify the number of weeks following influenza in which healthcare visits for the given condition were elevated among cases compared to controls. Our diff-in-diff analysis revealed two situations in which a given post-acute condition, which was elevated among cases relative to controls, at some point returned to a “baseline” following influenza: (1) the condition returned to a pre-influenza baseline where there was no significant difference between cases and controls, or (2) the condition returned to a new elevated-baseline where visits for the condition stabilized to a slightly elevated level among cases during the follow-up period. Thus, we considered two approaches to identify the number of weeks following influenza in which a condition was elevated among case patients compared to controls, based on these two patterns.

For conditions for which the incidence rate among cases returned to a baseline that was no different than controls (see for example acute bronchitis and cough in Figure 2), we used the number of consecutive weeks following the influenza diagnosis in which the incidence rate ratio from the interaction term $\boldsymbol{\beta}\left( {Week}_{w}:C{ase}_{c} \right)$ in the difference-in-differences model was significantly greater than zero.

Some conditions appeared to return to a higher-than-normal baseline, in which cases maintained a higher incidence rate over the study period following influenza (see for example pneumonia or fever). In such cases, we used the CUSUM (cumulative sum control chart) technique to identify the week in which the incidence rate ratio returned to this new baseline. To do so, we sorted the point estimates from the term $\boldsymbol{\beta}\left( {Week}_{w}:C{ase}_{c} \right)$ in reverse order, starting from 52 weeks post influenza to 1 week post influenza. Then we applied the CUSUM approach to this time series and identified the point of deviation.

**Appendix Tables**

**Appendix Table 1** – Diagnosis codes used to identify cases of influenza

| **Diagnosis Codes** | **ICD Version** | **Description** |
| --- | --- | --- |
| 487 | 9 | Influenza |
| 4870 | 9 | Influenza with pneumonia |
| 4871 | 9 | Influenza with other respiratory manifestations |
| 4878 | 9 | Influenza with other manifestations |
| 488 | 9 | Influenza due to certain identified influenza viruses |
| 4880 | 9 | Influenza due to identified avian influenza virus |
| 48801 | 9 | Influenza due to identified avian influenza virus with pneumonia |
| 48802 | 9 | Influenza due to identified avian influenza virus with other respiratory manifestations |
| 48809 | 9 | Influenza due to identified avian influenza virus with other manifestations |
| 4881 | 9 | Influenza due to identified 2009 H1N1 influenza virus |
| 48811 | 9 | Influenza due to identified 2009 H1N1 influenza virus with pneumonia |
| 48812 | 9 | Influenza due to identified 2009 H1N1 influenza virus with other respiratory manifestations |
| 48819 | 9 | Influenza due to identified 2009 H1N1 influenza virus with other manifestations |
| 4888 | 9 | Influenza due to novel influenza A |
| 48881 | 9 | Influenza due to identified novel influenza A virus with pneumonia |
| 48882 | 9 | Influenza due to identified novel influenza A virus with other respiratory manifestations |
| 48889 | 9 | Influenza due to identified novel influenza A virus with other manifestations |
| J09 | 10 | Influenza due to certain identified influenza viruses |
| J09X | 10 | Influenza due to identified novel influenza A virus |
| J09X1 | 10 | Influenza due to identified novel influenza A virus with pneumonia |
| J09X2 | 10 | Influenza due to identified novel influenza A virus with other respiratory manifestations |
| J09X3 | 10 | Influenza due to identified novel influenza A virus with gastrointestinal manifestations |
| J09X9 | 10 | Influenza due to identified novel influenza A virus with other manifestations |
| J10 | 10 | Influenza due to other identified influenza virus |
| J100 | 10 | Influenza due to other identified influenza virus with pneumonia |
| J1000 | 10 | Influenza due to other identified influenza virus with unspecified type of pneumonia |
| J1001 | 10 | Influenza due to other identified influenza virus with the same other identified influenza virus pneumonia |
| J1008 | 10 | Influenza due to other identified influenza virus with other specified pneumonia |
| J101 | 10 | Influenza due to other identified influenza virus with other respiratory manifestations |
| J102 | 10 | Influenza due to other identified influenza virus with gastrointestinal manifestations |
| J108 | 10 | Influenza due to other identified influenza virus with other manifestations |
| J1081 | 10 | Influenza due to other identified influenza virus with encephalopathy |
| J1082 | 10 | Influenza due to other identified influenza virus with myocarditis |
| J1083 | 10 | Influenza due to other identified influenza virus with otitis media |
| J1089 | 10 | Influenza due to other identified influenza virus with other manifestations |
| J11 | 10 | Influenza due to unidentified influenza virus |
| J110 | 10 | Influenza due to unidentified influenza virus with pneumonia |
| J1100 | 10 | Influenza due to unidentified influenza virus with unspecified type of pneumonia |
| J1108 | 10 | Influenza due to unidentified influenza virus with specified pneumonia |
| J111 | 10 | Influenza due to unidentified influenza virus with other respiratory manifestations |
| J112 | 10 | Influenza due to unidentified influenza virus with gastrointestinal manifestations |
| J118 | 10 | Influenza due to unidentified influenza virus with other manifestations |
| J1181 | 10 | Influenza due to unidentified influenza virus with encephalopathy |
| J1182 | 10 | Influenza due to unidentified influenza virus with myocarditis |
| J1183 | 10 | Influenza due to unidentified influenza virus with otitis media |
| J1189 | 10 | Influenza due to unidentified influenza virus with other manifestations |

**Appendix Table 2** – ICD-9 diagnosis conditions appearing in the “cough” focal cluster

| **ICD-9 Code** | **Description** | **% of times in focal cluster** |
| --- | --- | --- |
| 78060 | Fever, unspecified | 100 |
| 7862 | Cough | 100 |
| 462 | Acute pharyngitis | 100 |
| 4660 | Acute bronchitis | 100 |
| 486 | Pneumonia, organism unspecified | 100 |
| 07999 | Unspecified viral infection | 100 |
| 78650 | Chest pain, unspecified | 100 |
| 490 | Bronchitis, not specified as acute or chronic | 100 |
| 78605 | Shortness of breath | 100 |
| 7806 | Fever and other physiologic disturbances of temperature regulation | 100 |
| 78701 | Nausea with vomiting | 100 |
| 27651 | Dehydration | 100 |
| 78607 | Wheezing | 100 |
| 78703 | Vomiting alone | 100 |
| 49392 | Asthma, unspecified type, with (acute) exacerbation | 100 |
| 7802 | Syncope and collapse | 100 |
| 78061 | Fever presenting with conditions classified elsewhere | 100 |
| 51911 | Acute bronchospasm | 100 |
| 49121 | Obstructive chronic bronchitis with (acute) exacerbation | 100 |
| 4829 | Bacterial pneumonia, unspecified | 100 |
| 7931 | Lung field | 100 |
| 7850 | Tachycardia, unspecified | 100 |
| 79319 | Other nonspecific abnormal finding of lung field | 100 |
| 2768 | Hypopotassemia | 100 |
| 7869 | Other symptoms involving respiratory system and chest | 100 |
| 5180 | Pulmonary collapse | 100 |
| 28860 | Leukocytosis, unspecified | 100 |
| 64893 | Other current conditions classifiable elsewhere of mother, antepartum condition or complication | 100 |
| V679 | Unspecified follow-up examination | 100 |
| 485 | Bronchopneumonia, organism unspecified | 100 |
| 2761 | Hyposmolality and/or hyponatremia | 100 |
| 0389 | Unspecified septicemia | 100 |
| 4589 | Hypotension, unspecified | 100 |
| 481 | Pneumococcal pneumonia [Streptococcus pneumoniae pneumonia] | 100 |
| 78031 | Febrile convulsions (simple), unspecified | 100 |
| 4809 | Viral pneumonia, unspecified | 100 |
| 27650 | Volume depletion, unspecified | 100 |
| 7867 | Abnormal chest sounds | 100 |
| 2765 | Volume depletion | 100 |
| 48289 | Pneumonia due to other specified bacteria | 100 |
| 78630 | Hemoptysis, unspecified | 100 |
| 4838 | Pneumonia due to other specified organism | 100 |
| 27652 | Hypovolemia | 100 |
| V3000 | Single liveborn, born in hospital, delivered without mention of cesarean section | 100 |
| 64783 | Other specified infectious and parasitic diseases of mother, antepartum condition or complication | 100 |
| 4808 | Pneumonia due to other virus not elsewhere classified | 100 |
| 99590 | Systemic inflammatory response syndrome, unspecified | 100 |
| 3490 | Reaction to spinal or lumbar puncture | 100 |
| V642 | Surgical or other procedure not carried out because of patient's decision | 100 |
| 0578 | Other specified viral exanthemata | 100 |
| 4659 | Acute upper respiratory infections of unspecified site | 96.43 |
| 514 | Pulmonary congestion and hypostasis | 96.43 |
| 78097 | Altered mental status | 96.43 |
| 78064 | Chills (without fever) | 96.43 |
| 5183 | Pulmonary eosinophilia | 96.43 |
| 07889 | Other specified diseases due to viruses | 96.43 |
| 7863 | Hemoptysis | 96.43 |
| 51881 | Acute respiratory failure | 92.86 |
| 78609 | Other respiratory abnormalities | 89.29 |
| 5849 | Acute kidney failure, unspecified | 89.29 |
| 99591 | Sepsis | 89.29 |
| 99527 | Other drug allergy | 89.29 |
| 72888 | Rhabdomyolysis | 89.29 |
| 99592 | Severe sepsis | 89.29 |
| 79902 | Hypoxemia | 85.71 |
| 7908 | Viremia, unspecified | 39.29 |
| 07989 | Other specified viral infection | 39.29 |
| 475 | Peritonsillar abscess | 39.29 |
| 49390 | Asthma, unspecified type, unspecified | 35.71 |
| 3051 | Tobacco use disorder | 35.71 |
| 51889 | Other diseases of lung, not elsewhere classified | 35.71 |
| 78096 | Generalized pain | 35.71 |
| 075 | Infectious mononucleosis | 35.71 |
| 5119 | Unspecified pleural effusion | 35.71 |
| V0179 | Contact with or exposure to other viral diseases | 35.71 |
| 59080 | Pyelonephritis, unspecified | 35.71 |
| 7907 | Bacteremia | 35.71 |
| 5272 | Sialoadenitis | 35.71 |
| 0542 | Herpetic gingivostomatitis | 35.71 |
| 34831 | Metabolic encephalopathy | 35.71 |
| 7861 | Stridor | 35.71 |
| 42833 | Acute on chronic diastolic heart failure | 35.71 |
| 4821 | Pneumonia due to Pseudomonas | 35.71 |
| 78552 | Septic shock | 35.71 |
| 42821 | Acute systolic heart failure | 35.71 |
| 7864 | Abnormal sputum | 35.71 |
| 4619 | Acute sinusitis, unspecified | 21.43 |
| 3829 | Unspecified otitis media | 21.43 |
| 7840 | Headache | 21.43 |
| 38200 | Acute suppurative otitis media without spontaneous rupture of eardrum | 21.43 |
| 4739 | Unspecified sinusitis (chronic) | 21.43 |
| 4610 | Acute maxillary sinusitis | 21.43 |
| 463 | Acute tonsillitis | 21.43 |
| 47819 | Other disease of nasal cavity and sinuses | 21.43 |
| 460 | Acute nasopharyngitis [common cold] | 21.43 |
| 78702 | Nausea alone | 21.43 |
| 4280 | Congestive heart failure, unspecified | 21.43 |
| V709 | Unspecified general medical examination | 21.43 |
| 7841 | Throat pain | 21.43 |
| 4658 | Acute upper respiratory infections of other multiple sites | 21.43 |
| 4644 | Croup | 21.43 |
| 42789 | Other specified cardiac dysrhythmias | 21.43 |
| 49302 | Extrinsic asthma with (acute) exacerbation | 21.43 |
| V6759 | Other follow-up examination | 21.43 |
| 4293 | Cardiomegaly | 21.43 |
| 0549 | Herpes simplex without mention of complication | 21.43 |
| 46400 | Acute laryngitis without mention of obstruction | 21.43 |
| 5198 | Other diseases of respiratory system, not elsewhere classified | 21.43 |
| 2875 | Thrombocytopenia, unspecified | 21.43 |
| 46611 | Acute bronchiolitis due to respiratory syncytial virus (RSV) | 21.43 |
| 5110 | Pleurisy without mention of effusion or current tuberculosis | 21.43 |
| 1120 | Candidiasis of mouth | 21.43 |
| 0579 | Viral exanthem, unspecified | 21.43 |
| 28850 | Leukocytopenia, unspecified | 21.43 |
| 2888 | Other specified disease of white blood cells | 21.43 |
| 99520 | Unspecified adverse effect of unspecified drug, medicinal and biological substance | 21.43 |
| 38201 | Acute suppurative otitis media with spontaneous rupture of eardrum | 21.43 |
| 5070 | Pneumonitis due to inhalation of food or vomitus | 21.43 |
| 51882 | Other pulmonary insufficiency, not elsewhere classified | 21.43 |
| 0796 | Respiratory syncytial virus (RSV) | 21.43 |
| 5362 | Persistent vomiting | 21.43 |
| 29420 | Dementia, unspecified, without behavioral disturbance | 21.43 |
| 49122 | Obstructive chronic bronchitis with acute bronchitis | 21.43 |
| 4830 | Pneumonia due to mycoplasma pneumoniae | 21.43 |
| 52800 | Stomatitis and mucositis, unspecified | 21.43 |
| 46410 | Acute tracheitis without mention of obstruction | 21.43 |
| 6930 | Dermatitis due to drugs and medicines taken internally | 21.43 |
| 2762 | Acidosis | 21.43 |
| V1261 | Personal history of pneumonia (recurrent) | 21.43 |
| 51884 | Acute and chronic respiratory failure | 21.43 |
| 5191 | Other diseases of trachea and bronchus, not elsewhere classified | 21.43 |
| V1209 | Personal history of other infectious and parasitic diseases | 21.43 |
| 2760 | Hyperosmolality and/or hypernatremia | 21.43 |
| 4911 | Mucopurulent chronic bronchitis | 21.43 |
| 78606 | Tachypnea | 21.43 |
| 5109 | Empyema without mention of fistula | 21.43 |
| 41070 | Subendocardial infarction, episode of care unspecified | 21.43 |
| 5845 | Acute kidney failure with lesion of tubular necrosis | 21.43 |
| 5720 | Abscess of liver | 21.43 |
| 28419 | Other pancytopenia | 21.43 |
| 0380 | Streptococcal septicemia | 21.43 |
| 78602 | Orthopnea | 21.43 |
| 7837 | Adult failure to thrive | 21.43 |
| 0790 | Adenovirus infection in conditions classified elsewhere and of unspecified site | 21.43 |
| 78791 | Diarrhea | 17.86 |
| 78659 | Other chest pain | 17.86 |
| 78652 | Painful respiration | 17.86 |
| 5168 | Other specified alveolar and parietoalveolar pneumonopathies | 17.86 |
| 78079 | Other malaise and fatigue | 10.71 |
| 496 | Chronic airway obstruction, not elsewhere classified | 10.71 |
| 5589 | Other and unspecified noninfectious gastroenteritis and colitis | 10.71 |
| 4618 | Other acute sinusitis | 10.71 |
| V7260 | Laboratory examination, unspecified | 10.71 |
| 79431 | Nonspecific abnormal electrocardiogram [ECG] [EKG] | 10.71 |
| 46619 | Acute bronchiolitis due to other infectious organisms | 10.71 |
| 38100 | Acute nonsuppurative otitis media, unspecified | 10.71 |
| 4611 | Acute frontal sinusitis | 10.71 |
| 7847 | Epistaxis | 10.71 |
| 4650 | Acute laryngopharyngitis | 10.71 |
| 49391 | Asthma, unspecified type, with status asthmaticus | 10.71 |
| 49120 | Obstructive chronic bronchitis without exacerbation | 10.71 |
| 4928 | Other emphysema | 10.71 |
| 78600 | Respiratory abnormality, unspecified | 10.71 |
| 64683 | Other specified complications of pregnancy, antepartum condition or complication | 10.71 |
| 7993 | Debility, unspecified | 10.71 |
| 00845 | Intestinal infection due to Clostridium difficile | 10.71 |
| 4919 | Unspecified chronic bronchitis | 10.71 |
| 4910 | Simple chronic bronchitis | 10.71 |
| 586 | Renal failure, unspecified | 10.71 |
| 5733 | Hepatitis, unspecified | 10.71 |
| 5282 | Oral aphthae | 10.71 |
| 59010 | Acute pyelonephritis without lesion of renal medullary necrosis | 10.71 |
| 4789 | Other and unspecified diseases of upper respiratory tract | 10.71 |
| 49312 | Intrinsic asthma with (acute) exacerbation | 10.71 |
| 49322 | Chronic obstructive asthma with (acute) exacerbation | 10.71 |
| 51919 | Other diseases of trachea and bronchus | 10.71 |
| 04181 | Other specified bacterial infections in conditions classified elsewhere and of unspecified site, mycoplasma | 10.71 |
| 42490 | Endocarditis, valve unspecified, unspecified cause | 10.71 |
| 7827 | Spontaneous ecchymoses | 10.71 |
| V8522 | Body Mass Index 26.0-26.9, adult | 10.71 |
| V1269 | Personal history of other diseases of respiratory system | 10.71 |
| 5780 | Hematemesis | 10.71 |
| 4788 | Upper respiratory tract hypersensitivity reaction, site unspecified | 10.71 |
| 4918 | Other chronic bronchitis | 10.71 |
| 2930 | Delirium due to conditions classified elsewhere | 10.71 |
| V8534 | Body Mass Index 34.0-34.9, adult | 10.71 |
| 78099 | Other general symptoms | 7.14 |
| 38101 | Acute serous otitis media | 7.14 |
| V726 | Laboratory examination | 7.14 |
| 7999 | Other unknown and unspecified cause of morbidity and mortality | 7.14 |
| 0088 | Intestinal infection due to other organism, not elsewhere classified | 7.14 |
| 7089 | Urticaria, unspecified | 7.14 |
| 78009 | Other alteration of consciousness | 7.14 |
| 7969 | Other nonspecific abnormal findings | 7.14 |
| V1582 | Personal history of tobacco use | 7.14 |
| 2893 | Lymphadenitis, unspecified, except mesenteric | 7.14 |
| 7866 | Swelling, mass, or lump in chest | 7.14 |
| V140 | Personal history of allergy to penicillin | 7.14 |
| 28800 | Neutropenia, unspecified | 7.14 |
| 7892 | Splenomegaly | 7.14 |
| V811 | Screening for hypertension | 7.14 |
| 41071 | Subendocardial infarction, initial episode of care | 7.14 |
| V6406 | Vaccination not carried out because of patient refusal | 7.14 |
| 46420 | Acute laryngotracheitis without mention of obstruction | 7.14 |
| 0340 | Streptococcal sore throat | 3.57 |
| 5990 | Urinary tract infection, site not specified | 3.57 |
| 42731 | Atrial fibrillation | 3.57 |
| 7856 | Enlargement of lymph nodes | 3.57 |
| 78039 | Other convulsions | 3.57 |
| 7904 | Nonspecific elevation of levels of transaminase or lactic acid dehydrogenase [LDH] | 3.57 |
| 515 | Postinflammatory pulmonary fibrosis | 3.57 |
| 49320 | Chronic obstructive asthma, unspecified | 3.57 |
| 79311 | Solitary pulmonary nodule | 3.57 |
| 78499 | Other symptoms involving head and neck | 3.57 |
| 3824 | Unspecified suppurative otitis media | 3.57 |
| 683 | Acute lymphadenitis | 3.57 |
| 4580 | Orthostatic hypotension | 3.57 |
| 2769 | Electrolyte and fluid disorders not elsewhere classified | 3.57 |
| 49301 | Extrinsic asthma with status asthmaticus | 3.57 |
| 2752 | Disorders of magnesium metabolism | 3.57 |
| 04101 | Streptococcus infection in conditions classified elsewhere and of unspecified site, streptococcus, group A | 3.57 |
| 0341 | Scarlet fever | 3.57 |
| V5882 | Fitting and adjustment of nonvascular catheter, NEC | 3.57 |
| 5601 | Paralytic ileus | 3.57 |
| 79399 | Other nonspecific (abnormal) findings on radiological and other examinations of body structure | 3.57 |
| V142 | Personal history of allergy to sulfonamides | 3.57 |
| 07799 | Unspecified diseases of conjunctiva due to viruses | 3.57 |
| 5207 | Teething syndrome | 3.57 |
| 7824 | Jaundice, unspecified, not of newborn | 3.57 |
| V141 | Personal history of allergy to other antibiotic agent | 3.57 |
| 3643 | Unspecified iridocyclitis | 3.57 |
| V8532 | Body Mass Index 32.0-32.9, adult | 3.57 |
| 47829 | Other diseases of pharynx, not elsewhere classified | 3.57 |
| V048 | Other viral diseases | 3.57 |
| 9952 | Other and unspecified adverse effect of drug, medicinal and biological substance | 3.57 |
| 2870 | Allergic purpura | 3.57 |
| 4941 | Bronchiectasis with acute exacerbation | 3.57 |
| 7469 | Unspecified congenital anomaly of heart | 3.57 |
| V8535 | Body Mass Index 35.0-35.9, adult | 3.57 |
| 42823 | Acute on chronic systolic heart failure | 3.57 |
| 64880 | Abnormal glucose tolerance of mother, unspecified as to episode of care or not applicable | 3.57 |
| 49321 | Chronic obstructive asthma with status asthmaticus | 3.57 |
| 1983 | Secondary malignant neoplasm of brain and spinal cord | 3.57 |
| 78603 | Apnea | 3.57 |
| V749 | Screening examination for unspecified bacterial and spirochetal diseases | 3.57 |
| 42830 | Diastolic heart failure, unspecified | 3.57 |
| 38104 | Acute allergic serous otitis media | 3.57 |
| 25052 | Diabetes with ophthalmic manifestations, type II or unspecified type, uncontrolled | 3.57 |
| 2841 | Pancytopenia | 3.57 |
| 4471 | Stricture of artery | 3.57 |
| 5290 | Glossitis | 3.57 |

**Appendix Table 3** – ICD-10 diagnosis conditions appearing in the “cough” focal cluster

| **ICD-10 Code** | **Description** | **% of times in focal cluster** |
| --- | --- | --- |
| R509 | Fever, unspecified | 100 |
| R05 | Cough | 100 |
| J209 | Acute bronchitis, unspecified | 100 |
| B349 | Viral infection, unspecified | 100 |
| J189 | Pneumonia, unspecified organism | 100 |
| R0602 | Shortness of breath | 100 |
| J40 | Bronchitis, not specified as acute or chronic | 100 |
| R062 | Wheezing | 100 |
| E860 | Dehydration | 100 |
| R918 | Other nonspecific abnormal finding of lung field | 100 |
| J45901 | Unspecified asthma with (acute) exacerbation | 100 |
| R55 | Syncope and collapse | 100 |
| R000 | Tachycardia, unspecified | 100 |
| J9801 | Acute bronchospasm | 100 |
| J208 | Acute bronchitis due to other specified organisms | 100 |
| Z20828 | Contact with and (suspected) exposure to other viral communicable diseases | 100 |
| J181 | Lobar pneumonia, unspecified organism | 100 |
| E876 | Hypokalemia | 100 |
| J441 | Chronic obstructive pulmonary disease with (acute) exacerbation | 100 |
| J22 | Unspecified acute lower respiratory infection | 100 |
| A419 | Sepsis, unspecified organism | 100 |
| J159 | Unspecified bacterial pneumonia | 100 |
| Z8709 | Personal history of other diseases of the respiratory system | 100 |
| E871 | Hypo-osmolality and hyponatremia | 100 |
| I959 | Hypotension, unspecified | 100 |
| J9601 | Acute respiratory failure with hypoxia | 100 |
| J180 | Bronchopneumonia, unspecified organism | 100 |
| M7910 | Myalgia, unspecified site | 100 |
| R5600 | Simple febrile convulsions | 100 |
| R042 | Hemoptysis | 100 |
| G933 | Postviral fatigue syndrome | 100 |
| R091 | Pleurisy | 100 |
| J188 | Other pneumonia, unspecified organism | 100 |
| E869 | Volume depletion, unspecified | 100 |
| J129 | Viral pneumonia, unspecified | 100 |
| J168 | Pneumonia due to other specified infectious organisms | 100 |
| O99512 | Diseases of the respiratory system complicating pregnancy, second trimester | 100 |
| L270 | Generalized skin eruption due to drugs and medicaments taken internally | 100 |
| T887XXA | Unspecified adverse effect of drug or medicament, initial encounter | 100 |
| J158 | Pneumonia due to other specified bacteria | 100 |
| O99513 | Diseases of the respiratory system complicating pregnancy, third trimester | 100 |
| O99511 | Diseases of the respiratory system complicating pregnancy, first trimester | 100 |
| J36 | Peritonsillar abscess | 100 |
| J9809 | Other diseases of bronchus, not elsewhere classified | 100 |
| J202 | Acute bronchitis due to streptococcus | 100 |
| R061 | Stridor | 100 |
| R0603 | Acute respiratory distress | 100 |
| R6510 | Systemic inflammatory response syndrome (SIRS) of non-infectious origin without acute organ dysfunction | 100 |
| M6282 | Rhabdomyolysis | 100 |
| G9341 | Metabolic encephalopathy | 100 |
| R093 | Abnormal sputum | 100 |
| R112 | Nausea with vomiting, unspecified | 92.86 |
| R0902 | Hypoxemia | 92.86 |
| I5031 | Acute diastolic (congestive) heart failure | 85.71 |
| N179 | Acute kidney failure, unspecified | 57.14 |
| E872 | Acidosis | 57.14 |
| B09 | Unspecified viral infection characterized by skin and mucous membrane lesions | 50 |
| B974 | Respiratory syncytial virus as the cause of diseases classified elsewhere | 50 |
| R0682 | Tachypnea, not elsewhere classified | 50 |
| J029 | Acute pharyngitis, unspecified | 32.14 |
| J069 | Acute upper respiratory infection, unspecified | 32.14 |
| R5081 | Fever presenting with conditions classified elsewhere | 32.14 |
| R1110 | Vomiting, unspecified | 32.14 |
| H66001 | Acute suppurative otitis media without spontaneous rupture of ear drum, right ear | 32.14 |
| B9789 | Other viral agents as the cause of diseases classified elsewhere | 32.14 |
| R0600 | Dyspnea, unspecified | 32.14 |
| R0989 | Other specified symptoms and signs involving the circulatory and respiratory systems | 32.14 |
| Z09 | Encounter for follow-up examination after completed treatment for conditions other than malignant neoplasm | 32.14 |
| R6883 | Chills (without fever) | 32.14 |
| J9811 | Atelectasis | 32.14 |
| B338 | Other specified viral diseases | 32.14 |
| B348 | Other viral infections of unspecified site | 32.14 |
| J200 | Acute bronchitis due to Mycoplasma pneumoniae | 32.14 |
| J157 | Pneumonia due to Mycoplasma pneumoniae | 32.14 |
| J440 | Chronic obstructive pulmonary disease with acute lower respiratory infection | 32.14 |
| K1120 | Sialoadenitis, unspecified | 32.14 |
| H66011 | Acute suppurative otitis media with spontaneous rupture of ear drum, right ear | 32.14 |
| B950 | Streptococcus, group A, as the cause of diseases classified elsewhere | 32.14 |
| T50905A | Adverse effect of unspecified drugs, medicaments and biological substances, initial encounter | 32.14 |
| J0410 | Acute tracheitis without obstruction | 32.14 |
| J042 | Acute laryngotracheitis | 32.14 |
| I5023 | Acute on chronic systolic (congestive) heart failure | 32.14 |
| J206 | Acute bronchitis due to rhinovirus | 32.14 |
| H65111 | Acute and subacute allergic otitis media (mucoid) (sanguinous) (serous), right ear | 32.14 |
| N19 | Unspecified kidney failure | 32.14 |
| H65112 | Acute and subacute allergic otitis media (mucoid) (sanguinous) (serous), left ear | 32.14 |
| K750 | Abscess of liver | 28.57 |
| H6691 | Otitis media, unspecified, right ear | 25 |
| H65199 | Other acute nonsuppurative otitis media, unspecified ear | 25 |
| J00 | Acute nasopharyngitis [common cold] | 21.43 |
| D72819 | Decreased white blood cell count, unspecified | 21.43 |
| D709 | Neutropenia, unspecified | 17.86 |
| R079 | Chest pain, unspecified | 10.71 |
| H6692 | Otitis media, unspecified, left ear | 10.71 |
| H66002 | Acute suppurative otitis media without spontaneous rupture of ear drum, left ear | 10.71 |
| H6693 | Otitis media, unspecified, bilateral | 10.71 |
| H66003 | Acute suppurative otitis media without spontaneous rupture of ear drum, bilateral | 10.71 |
| J4521 | Mild intermittent asthma with (acute) exacerbation | 10.71 |
| J4541 | Moderate persistent asthma with (acute) exacerbation | 10.71 |
| B2790 | Infectious mononucleosis, unspecified without complication | 10.71 |
| B370 | Candidal stomatitis | 10.71 |
| B001 | Herpesviral vesicular dermatitis | 10.71 |
| Z9289 | Personal history of other medical treatment | 10.71 |
| Z8701 | Personal history of pneumonia (recurrent) | 10.71 |
| J9600 | Acute respiratory failure, unspecified whether with hypoxia or hypercapnia | 10.71 |
| A493 | Mycoplasma infection, unspecified site | 10.71 |
| J4551 | Severe persistent asthma with (acute) exacerbation | 10.71 |
| O26892 | Other specified pregnancy related conditions, second trimester | 10.71 |
| J0141 | Acute recurrent pansinusitis | 10.71 |
| G309 | Alzheimer's disease, unspecified | 10.71 |
| J811 | Chronic pulmonary edema | 10.71 |
| D61818 | Other pancytopenia | 10.71 |
| K920 | Hematemesis | 10.71 |
| R260 | Ataxic gait | 10.71 |
| J80 | Acute respiratory distress syndrome | 10.71 |
| L049 | Acute lymphadenitis, unspecified | 10.71 |
| J45902 | Unspecified asthma with status asthmaticus | 10.71 |
| Z119 | Encounter for screening for infectious and parasitic diseases, unspecified | 10.71 |
| N6311 | Unspecified lump in the right breast, upper outer quadrant | 10.71 |
| F0281 | Dementia in other diseases classified elsewhere with behavioral disturbance | 10.71 |
| E11649 | Type 2 diabetes mellitus with hypoglycemia without coma | 10.71 |
| T1490XA | Injury, unspecified, initial encounter | 10.71 |
| F17220 | Nicotine dependence, chewing tobacco, uncomplicated | 10.71 |
| Z3A26 | 26 weeks gestation of pregnancy | 10.71 |
| Z886 | Allergy status to analgesic agent status | 10.71 |
| J0190 | Acute sinusitis, unspecified | 7.14 |
| J45909 | Unspecified asthma, uncomplicated | 7.14 |
| R0981 | Nasal congestion | 7.14 |
| R52 | Pain, unspecified | 7.14 |
| J0100 | Acute maxillary sinusitis, unspecified | 7.14 |
| R070 | Pain in throat | 7.14 |
| J028 | Acute pharyngitis due to other specified organisms | 7.14 |
| F17210 | Nicotine dependence, cigarettes, uncomplicated | 7.14 |
| R9431 | Abnormal electrocardiogram [ECG] [EKG] | 7.14 |
| J050 | Acute obstructive laryngitis [croup] | 7.14 |
| R69 | Illness, unspecified | 7.14 |
| D72829 | Elevated white blood cell count, unspecified | 7.14 |
| J988 | Other specified respiratory disorders | 7.14 |
| J984 | Other disorders of lung | 7.14 |
| I517 | Cardiomegaly | 7.14 |
| J4531 | Mild persistent asthma with (acute) exacerbation | 7.14 |
| D696 | Thrombocytopenia, unspecified | 7.14 |
| R0689 | Other abnormalities of breathing | 7.14 |
| R4182 | Altered mental status, unspecified | 7.14 |
| R1111 | Vomiting without nausea | 7.14 |
| K120 | Recurrent oral aphthae | 7.14 |
| H6642 | Suppurative otitis media, unspecified, left ear | 7.14 |
| Z3689 | Encounter for other specified antenatal screening | 7.14 |
| R7881 | Bacteremia | 7.14 |
| Z2821 | Immunization not carried out because of patient refusal | 7.14 |
| Z7182 | Exercise counseling | 7.14 |
| K121 | Other forms of stomatitis | 7.14 |
| I4892 | Unspecified atrial flutter | 7.14 |
| J410 | Simple chronic bronchitis | 7.14 |
| M50321 | Other cervical disc degeneration at C4-C5 level | 7.14 |
| Z7984 | Long term (current) use of oral hypoglycemic drugs | 7.14 |
| L040 | Acute lymphadenitis of face, head and neck | 7.14 |
| F0280 | Dementia in other diseases classified elsewhere without behavioral disturbance | 7.14 |
| H35033 | Hypertensive retinopathy, bilateral | 7.14 |
| Z7951 | Long term (current) use of inhaled steroids | 7.14 |
| I712 | Thoracic aortic aneurysm, without rupture | 7.14 |
| Z9181 | History of falling | 7.14 |
| R358 | Other polyuria | 7.14 |
| B9620 | Unspecified Escherichia coli [E. coli] as the cause of diseases classified elsewhere | 7.14 |
| R791 | Abnormal coagulation profile | 7.14 |
| A491 | Streptococcal infection, unspecified site | 7.14 |
| G4719 | Other hypersomnia | 7.14 |
| J399 | Disease of upper respiratory tract, unspecified | 7.14 |
| K591 | Functional diarrhea | 7.14 |
| J690 | Pneumonitis due to inhalation of food and vomit | 7.14 |
| G4089 | Other seizures | 7.14 |
| I259 | Chronic ischemic heart disease, unspecified | 7.14 |
| D6489 | Other specified anemias | 7.14 |
| I6529 | Occlusion and stenosis of unspecified carotid artery | 7.14 |
| I6789 | Other cerebrovascular disease | 7.14 |
| H401132 | Primary open-angle glaucoma, bilateral, moderate stage | 7.14 |
| E7801 | Familial hypercholesterolemia | 7.14 |
| I5033 | Acute on chronic diastolic (congestive) heart failure | 7.14 |
| B359 | Dermatophytosis, unspecified | 7.14 |
| I5030 | Unspecified diastolic (congestive) heart failure | 7.14 |
| I2720 | Pulmonary hypertension, unspecified | 7.14 |
| R3121 | Asymptomatic microscopic hematuria | 7.14 |
| I8312 | Varicose veins of left lower extremity with inflammation | 7.14 |
| R338 | Other retention of urine | 7.14 |
| Z363 | Encounter for antenatal screening for malformations | 7.14 |
| H6521 | Chronic serous otitis media, right ear | 7.14 |
| S3992XA | Unspecified injury of lower back, initial encounter | 7.14 |
| M353 | Polymyalgia rheumatica | 7.14 |
| Z3A08 | 8 weeks gestation of pregnancy | 7.14 |
| Z9989 | Dependence on other enabling machines and devices | 7.14 |
| H9311 | Tinnitus, right ear | 7.14 |
| R311 | Benign essential microscopic hematuria | 7.14 |
| K5904 | Chronic idiopathic constipation | 7.14 |
| H60391 | Other infective otitis externa, right ear | 7.14 |
| O209 | Hemorrhage in early pregnancy, unspecified | 7.14 |
| F325 | Major depressive disorder, single episode, in full remission | 7.14 |
| J0181 | Other acute recurrent sinusitis | 7.14 |
| C649 | Malignant neoplasm of unspecified kidney, except renal pelvis | 7.14 |
| R7982 | Elevated C-reactive protein (CRP) | 7.14 |
| N185 | Chronic kidney disease, stage 5 | 7.14 |
| Z85850 | Personal history of malignant neoplasm of thyroid | 7.14 |
| D72828 | Other elevated white blood cell count | 7.14 |
| J9690 | Respiratory failure, unspecified, unspecified whether with hypoxia or hypercapnia | 7.14 |
| Z3A18 | 18 weeks gestation of pregnancy | 7.14 |
| I440 | Atrioventricular block, first degree | 7.14 |
| H25811 | Combined forms of age-related cataract, right eye | 7.14 |
| C7800 | Secondary malignant neoplasm of unspecified lung | 7.14 |
| E0789 | Other specified disorders of thyroid | 7.14 |
| O321XX0 | Maternal care for breech presentation, not applicable or unspecified | 7.14 |
| J370 | Chronic laryngitis | 7.14 |
| R700 | Elevated erythrocyte sedimentation rate | 7.14 |
| G541 | Lumbosacral plexus disorders | 7.14 |
| M6250 | Muscle wasting and atrophy, not elsewhere classified, unspecified site | 7.14 |
| R4701 | Aphasia | 7.14 |
| A6000 | Herpesviral infection of urogenital system, unspecified | 7.14 |
| C561 | Malignant neoplasm of right ovary | 7.14 |
| D469 | Myelodysplastic syndrome, unspecified | 7.14 |
| D860 | Sarcoidosis of lung | 7.14 |
| I5020 | Unspecified systolic (congestive) heart failure | 7.14 |
| B002 | Herpesviral gingivostomatitis and pharyngotonsillitis | 7.14 |
| F8082 | Social pragmatic communication disorder | 7.14 |
| O219 | Vomiting of pregnancy, unspecified | 7.14 |
| Z360 | Encounter for antenatal screening for chromosomal anomalies | 7.14 |
| F3130 | Bipolar disorder, current episode depressed, mild or moderate severity, unspecified | 7.14 |
| M859 | Disorder of bone density and structure, unspecified | 7.14 |
| N6321 | Unspecified lump in the left breast, upper outer quadrant | 7.14 |
| G610 | Guillain-Barre syndrome | 7.14 |
| Z3A24 | 24 weeks gestation of pregnancy | 7.14 |
| A0472 | Enterocolitis due to Clostridium difficile, not specified as recurrent | 7.14 |
| M5023 | Other cervical disc displacement, cervicothoracic region | 7.14 |
| D573 | Sickle-cell trait | 7.14 |
| M50123 | Cervical disc disorder at C6-C7 level with radiculopathy | 7.14 |
| K8590 | Acute pancreatitis without necrosis or infection, unspecified | 7.14 |
| N610 | Mastitis without abscess | 7.14 |
| N852 | Hypertrophy of uterus | 7.14 |
| Z3A21 | 21 weeks gestation of pregnancy | 7.14 |
| J90 | Pleural effusion, not elsewhere classified | 3.57 |
| H6641 | Suppurative otitis media, unspecified, right ear | 3.57 |
| Z2820 | Immunization not carried out because of patient decision for unspecified reason | 3.57 |
| J0111 | Acute recurrent frontal sinusitis | 3.57 |
| Z112 | Encounter for screening for other bacterial diseases | 3.57 |
| N12 | Tubulo-interstitial nephritis, not specified as acute or chronic | 3.57 |
| Z885 | Allergy status to narcotic agent status | 3.57 |
| H7291 | Unspecified perforation of tympanic membrane, right ear | 3.57 |
| H66012 | Acute suppurative otitis media with spontaneous rupture of ear drum, left ear | 3.57 |
| H9211 | Otorrhea, right ear | 3.57 |
| I313 | Pericardial effusion (noninflammatory) | 3.57 |
| R3989 | Other symptoms and signs involving the genitourinary system | 3.57 |
| M1000 | Idiopathic gout, unspecified site | 3.57 |
| I38 | Endocarditis, valve unspecified | 3.57 |
| H906 | Mixed conductive and sensorineural hearing loss, bilateral | 3.57 |
| K8050 | Calculus of bile duct without cholangitis or cholecystitis without obstruction | 3.57 |
| Z3A30 | 30 weeks gestation of pregnancy | 3.57 |
| H02831 | Dermatochalasis of right upper eyelid | 3.57 |
| S29011A | Strain of muscle and tendon of front wall of thorax, initial encounter | 3.57 |
| O09513 | Supervision of elderly primigravida, third trimester | 3.57 |
| D690 | Allergic purpura | 3.57 |
| G43919 | Migraine, unspecified, intractable, without status migrainosus | 3.57 |
| H26493 | Other secondary cataract, bilateral | 3.57 |
| H60502 | Unspecified acute noninfective otitis externa, left ear | 3.57 |
| Z3A31 | 31 weeks gestation of pregnancy | 3.57 |
| D701 | Agranulocytosis secondary to cancer chemotherapy | 3.57 |
| Z3A10 | 10 weeks gestation of pregnancy | 3.57 |
| S4992XA | Unspecified injury of left shoulder and upper arm, initial encounter | 3.57 |

**Appendix Table 4** – ICD-9 conditions excluded from aggregation

| **ICD 9 codes** | **ICD 9 code descriptions** |
| --- | --- |
| 29420 | Dementia, unspecified, without behavioral disturbance |
| V6759 | Other follow-up examination |
| 64783 | Other specified infectious and parasitic diseases of mother, antepartum condition or complication |
| V1269 | Personal history of other diseases of respiratory system |
| V1209 | Personal history of other infectious and parasitic diseases |
| V1261 | Personal history of pneumonia (recurrent) |
| V679 | Unspecified follow-up examination |
| V6406 | Vaccination not carried out because of patient refusal |

**Appendix Table 5** – ICD-10 conditions excluded from aggregation

| **ICD 10 codes** | **ICD 10 code descriptions** |
| --- | --- |
|  |  |
| Z3A18 | 18 weeks gestation of pregnancy |
| Z3A08 | 8 weeks gestation of pregnancy |
| O99511 | Diseases of the respiratory system complicating pregnancy, first trimester |
| Z09 | Encounter for follow-up examination after completed treatment for conditions other than malignant neoplasm |
| F17210 | Nicotine dependence, cigarettes, uncomplicated |
| O26892 | Other specified pregnancy related conditions, second trimester |
| Z8709 | Personal history of other diseases of the respiratory system |
| Z8701 | Personal history of pneumonia (recurrent) |
| F8082 | Social pragmatic communication disorder |

**Appendix Table 6** – Aggregated conditions excluded due to patterns identified in the difference-in-difference analysis that were inconsistent with post-acute sequelea. See Appendix Figure H for the patterns in difference-in-difference results that were evaluated. For example, acute myocardial infarction demonstrated a continuously increasing effect after the influenza event and clostridium difficile infections continuously decreased on either side of the influenza event. Each of these patterns were inconsistent with concept of post-acute sequelea.

| **Aggregated Category** | **ICD-9 Codes** | **ICD-9 Label** | **ICD-10 Codes** | **ICD-10 Label** |
| --- | --- | --- | --- | --- |
| Acute myocardial infarction | 410 | Acute myocardial infarction | I21 | Acute myocardial infarction |
| Adult failure to thrive | 7837 | Adult failure to thrive | R627 | Adult failure to thrive |
| Bronchiectasis excerbation | 4941 | Bronchiectasis with acute exacerbation | J471 | Bronchiectasis with acute exacerbation |
| Clostridium difficile | 845 | Intestinal infection due to Clostridium difficile | A047 | Enterocolitis due to Clostridium Difficile |
| Empyema without mention of fistula | 5109 | Empyema without mention of fistula | J869 | Pyothorax without fistula |
| Illness, unspecified | 7999 | Other unknown and unspecified cause of morbidity and mortality | R69 | Illness, unspecified |
| Illness, unspecified | 79989 | Other ill-defined conditions |  |  |
| Myalgia, unspecified site | 7291 | myalgia and myositis | M7910 | Myalgia, unspecified site |
| Other general symptoms | 78099 | Other general symptoms | R6889 | Other general symptoms and signs |
| Perforation of tympanic membrane | 3842 | Perforation of tympanic membrane | H72 | Perforation of tympanic membrane |
| Sepsis | 99591 | Sepsis | A419 | Sepsis, unspecified organism |
| Sepsis | 389 | Unspecified septicemia | |  |
| Spontaneous ecchymoses | 7827 | Spontaneous ecchymoses | R233 | Spontaneous ecchymoses |
| Teething syndrome | 5207 | Teething syndrome | K007 | Teething syndrome |
| Viral eanthems | 579 | Viral exanthem, unspecified | B09 | Unspecified viral infection characterized by skin and mucous membrane lesions |
| Viral eanthems | 578 | Other specified viral exanthemata | | |

**Appendix Figures**

**Appendix Figures A1-A28** – Cluster trends for ICD-9 codes using k=3:30

(See attachment FigA1_A28.pdf)

**Appendix Figures B1-B28** – Cluster trends for ICD-10 codes using k=3:30

(See attachment FigB1_B28.pdf)

**Appendix Figures C**– Cluster trends for ICD-9 codes using k=30, highlighting the cluster shapes deemed to be a plausible candidate for evaluation. Plausible shapes are highlighted in red.

**Appendix Figures D**– Cluster trends for ICD-10 codes using k=30, highlighting the cluster shapes deemed to be a plausible candidate for evaluation. Plausible shapes are highlighted in red.

**Appendix Figure E** – Difference-in-differences results for selected conditions representing potential post-influenza sequelae

**Appendix Figures F** – Difference-in-differences results for ICD-9 conditions

(See attachment FigF_icd9_DiD_res.pdf)

**Appendix Figures G** – Difference-in-differences results for ICD-10 conditions

(See attachment FigG_icd9_DiD_res.pdf)

**Appendix Figures H** – Difference-in-differences results for the final 80 aggregated conditions that were evaluated

(See attachment Appendix_Figure_H.pdf)
