# Supplementary material for: Estimating the Range and Duration of Post-influenza Symptoms, Conditions, and Diseases
Source: Open Forum Infect Dis. 2026 Oct 6;13(10):ofag629. doi: 10.1093/ofid/ofag629 (PMC13660689; doi:10.1093/ofid/ofag629)

Figure A1: k = 3

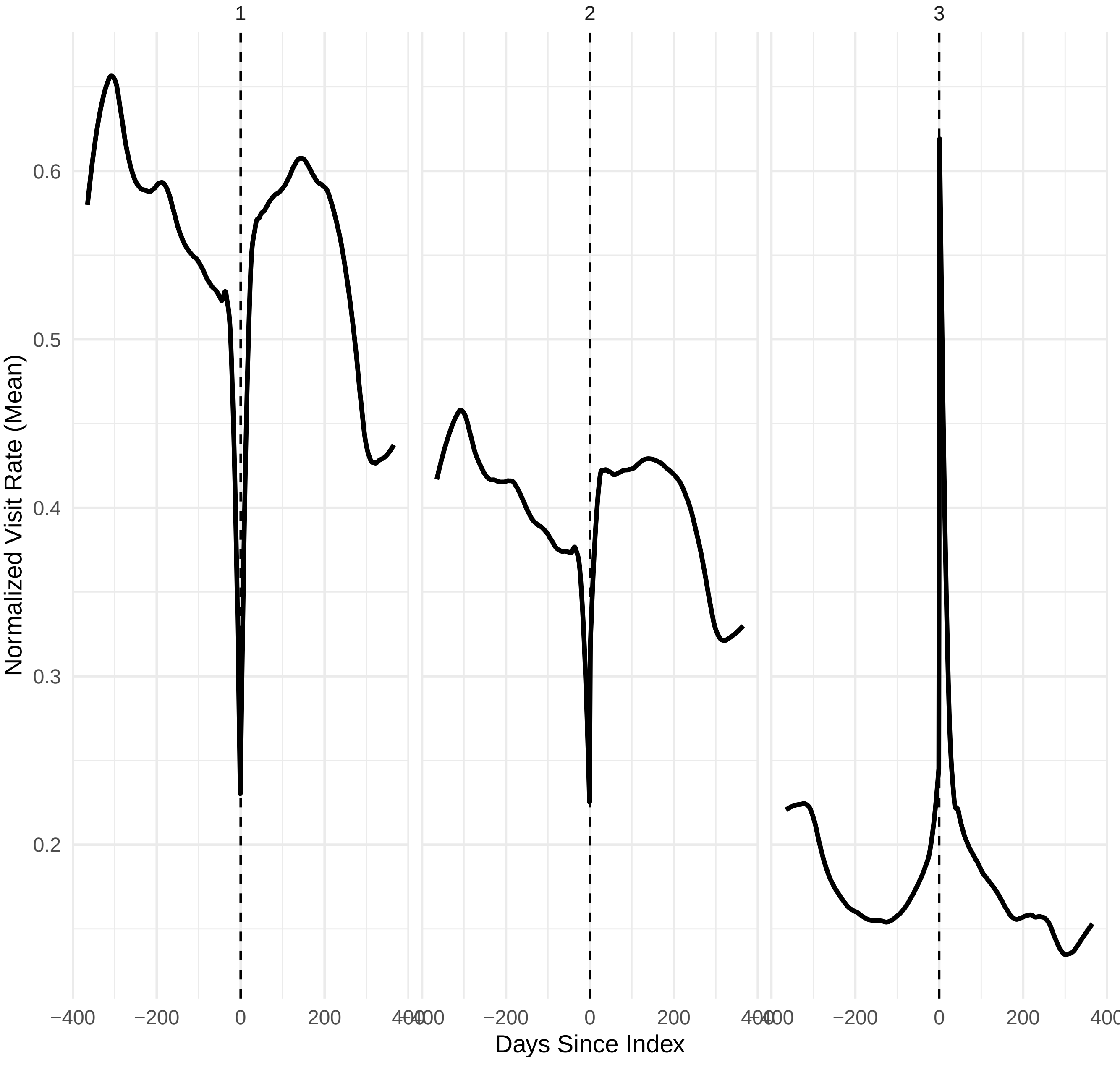
1
2
3
Normalized Visit Rate (Mean)
0.6
0.5
0.4
0.3
0.2
−400
−200
0
200
400
Days Since Index

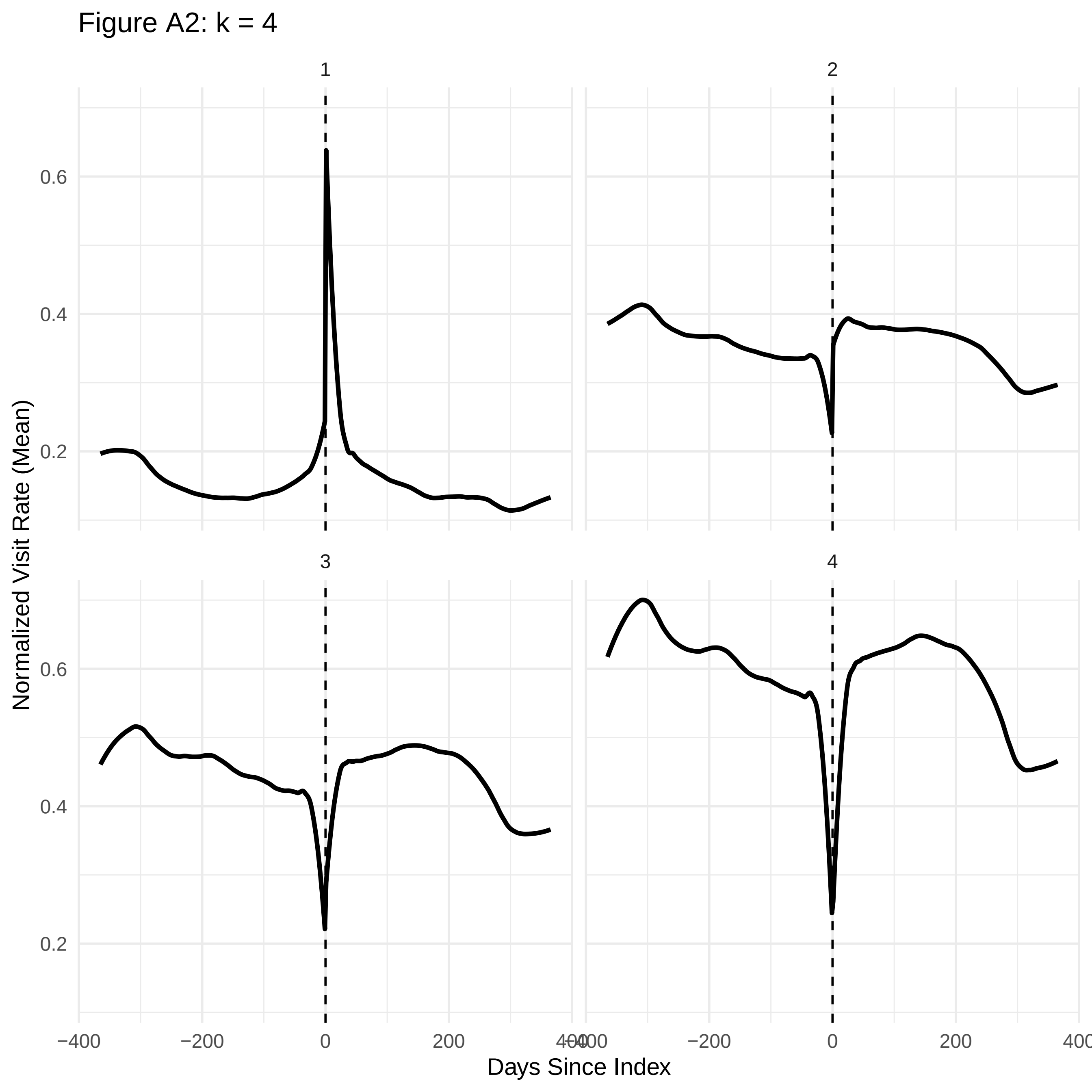

Figure A2: k = 4
1
2
3
4
0.2
0.4
0.6
−400
−200
0
200
400
Normalized Visit Rate (Mean)
Days Since Index

Figure A3: k = 5

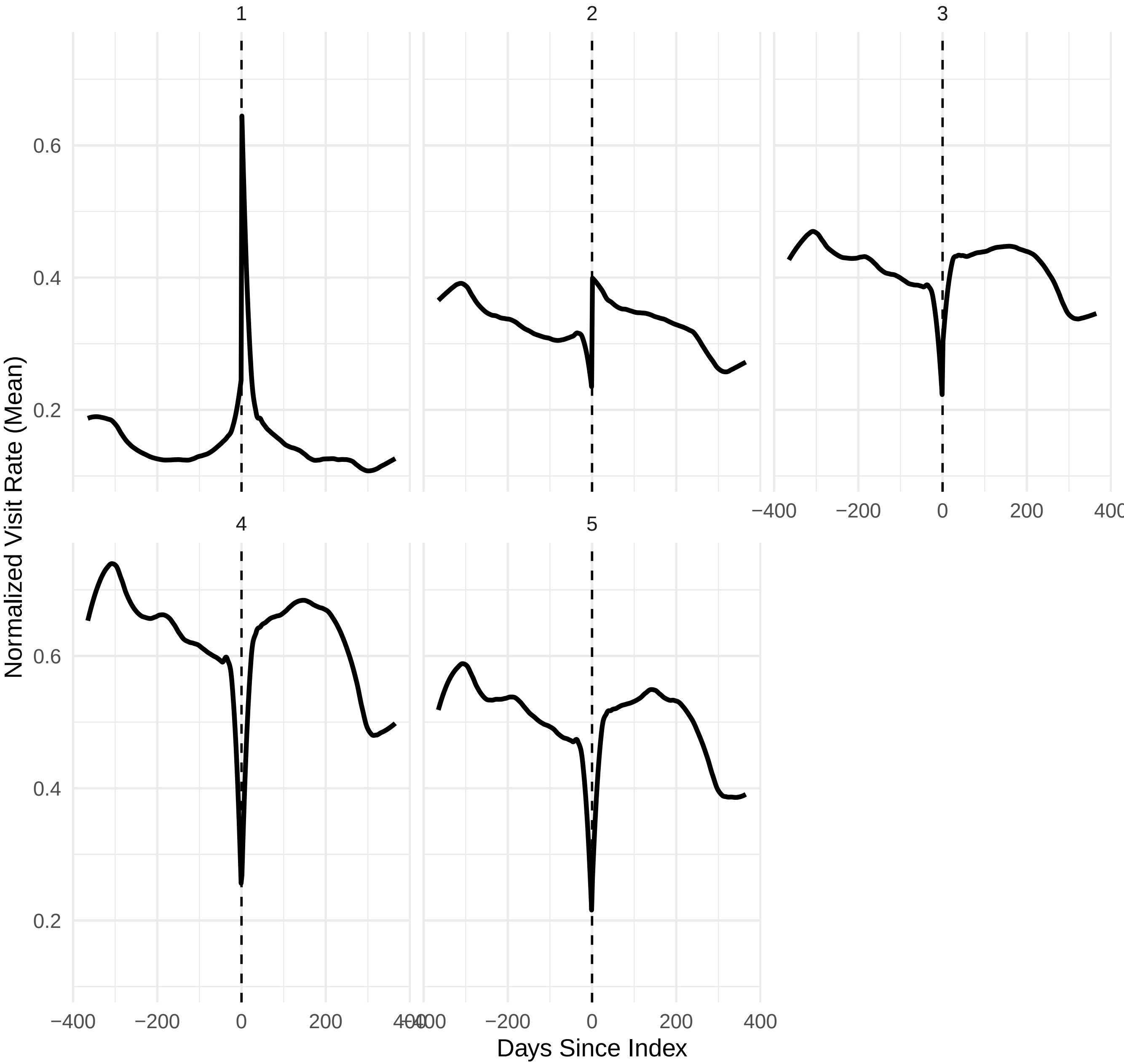

1
2
3
4
5
0.6
0.4
0.2
−400
−200
0
200
400
Normalized Visit Rate (Mean)
Days Since Index

## Figure A4: k = 6

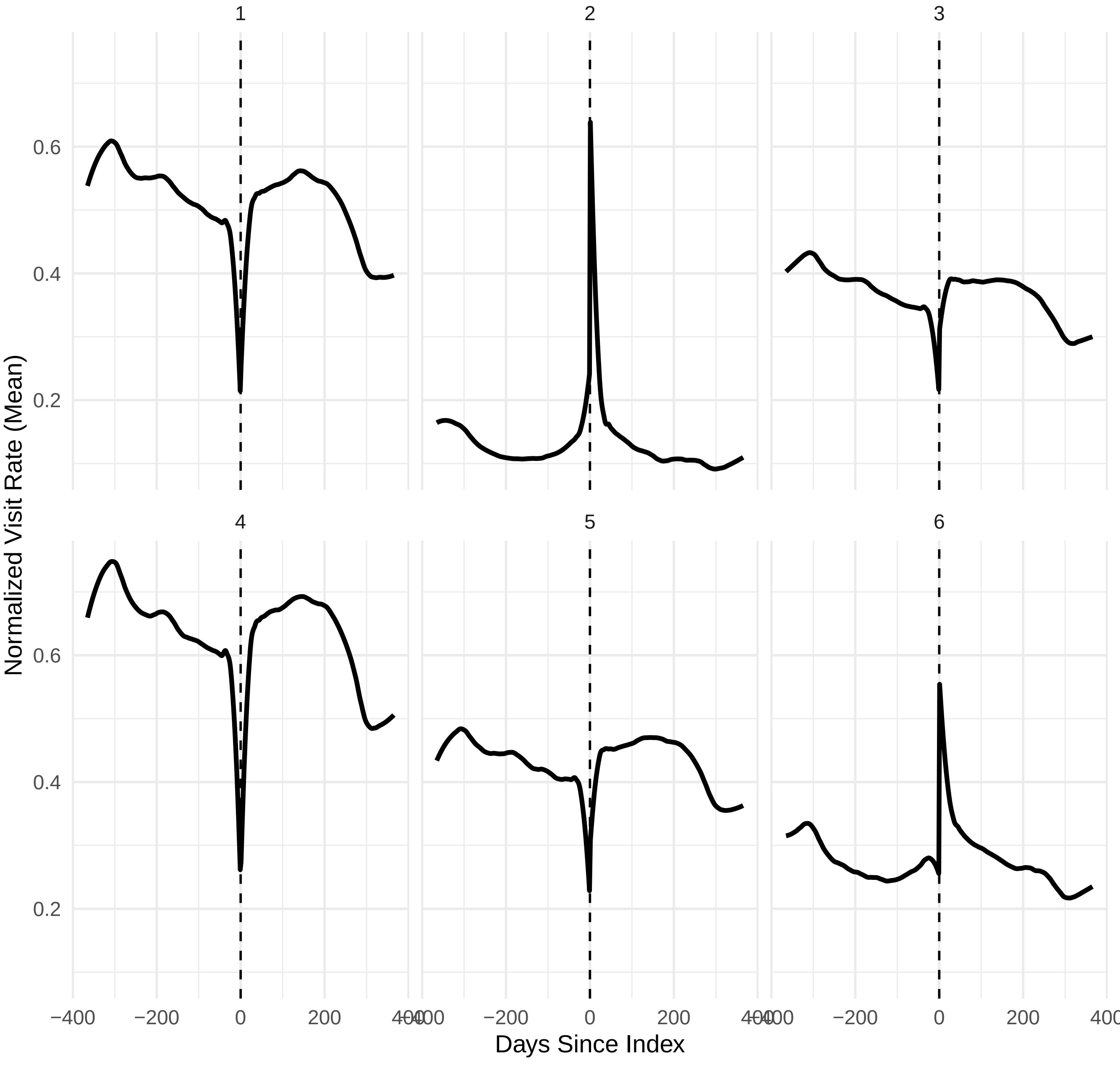
1
2
3
4
5
6
0.6
0.4
0.2
Normalized Visit Rate (Mean)
−400
−200
0
200
400
Days Since Index

Figure A5: k = 7

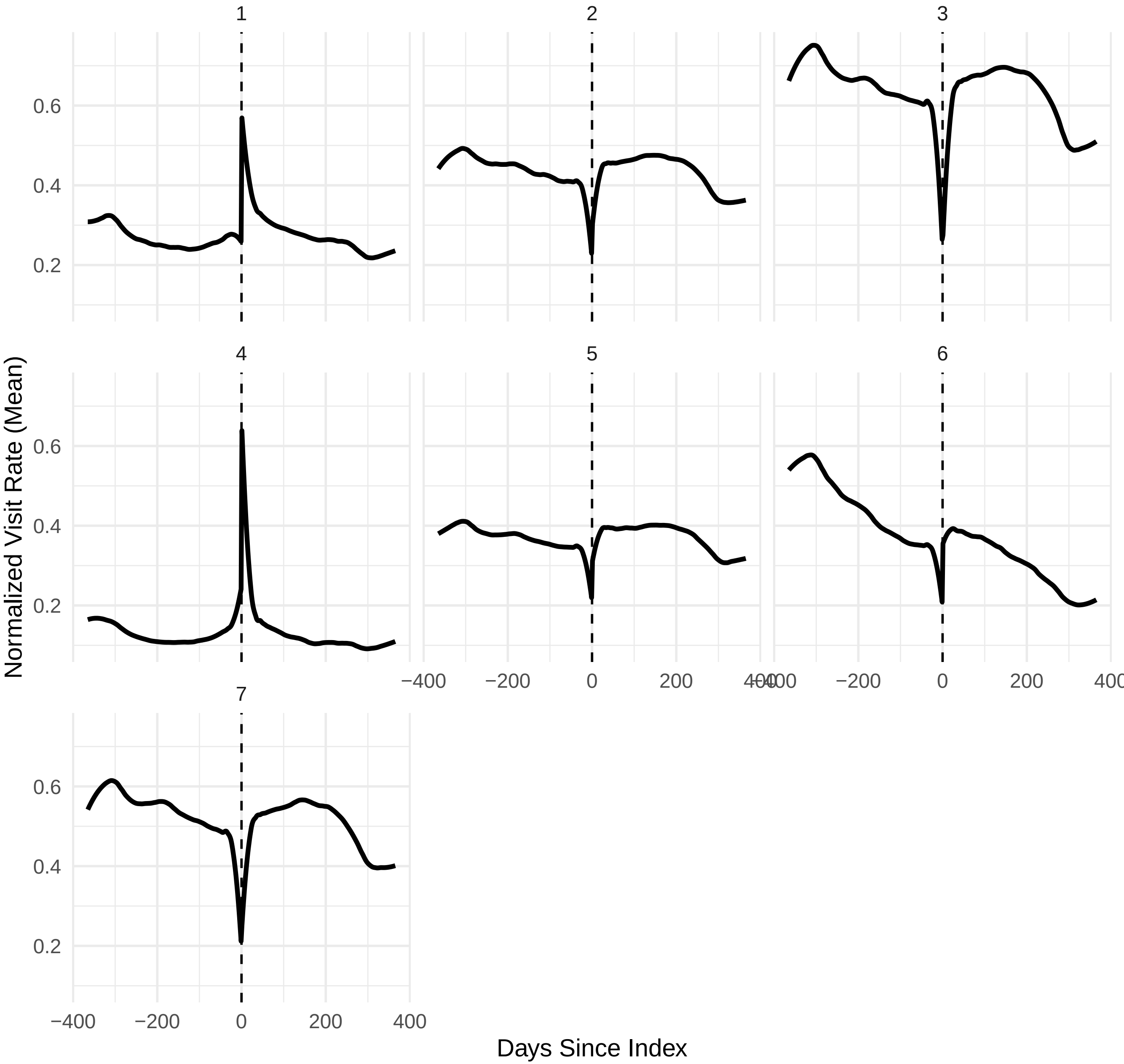
1
2
3
4
5
6
7
0.6
0.4
0.2
−400
−200
0
200
400
Normalized Visit Rate (Mean)
Days Since Index

Figure A6: k = 8

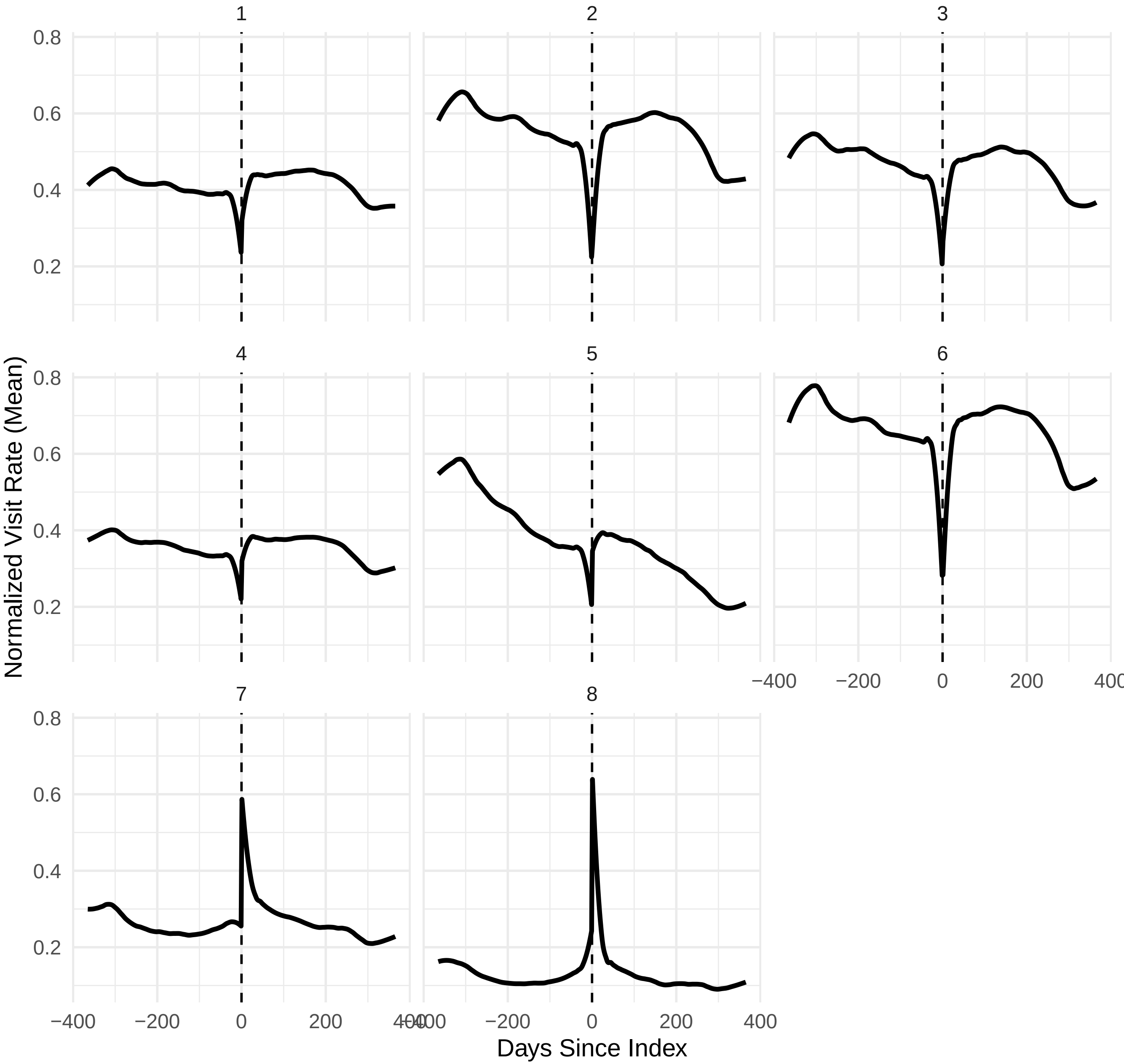

1
2
3
4
5
6
7
8
0.8
0.6
0.4
0.2
−400
−200
0
200
400
Normalized Visit Rate (Mean)
Days Since Index

Figure A7: k = 9

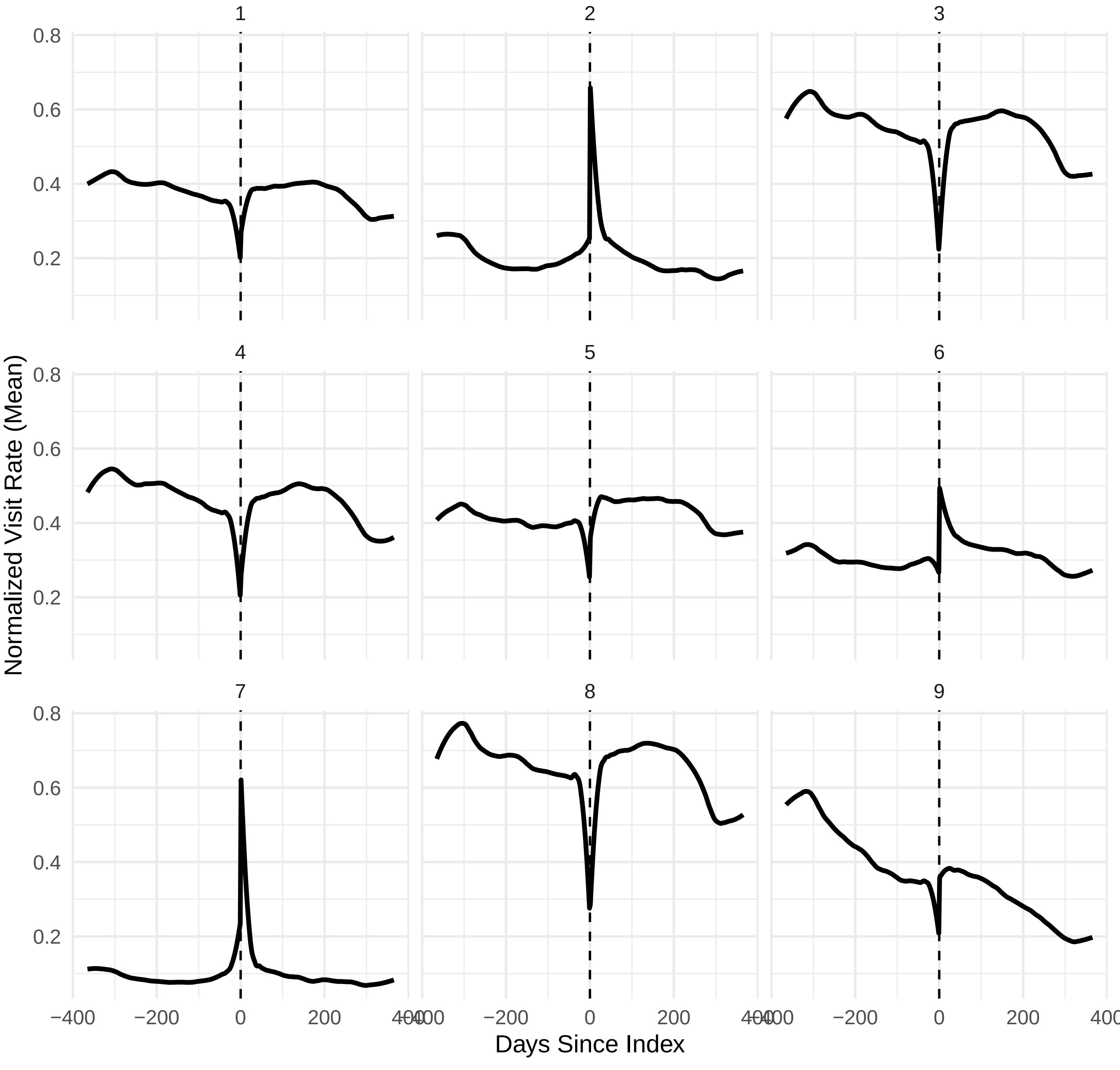

1
2
3
4
5
6
7
8
9
0.8
0.6
0.4
0.2
−400
−200
0
200
400
Normalized Visit Rate (Mean)
Days Since Index

Figure A8: k = 10

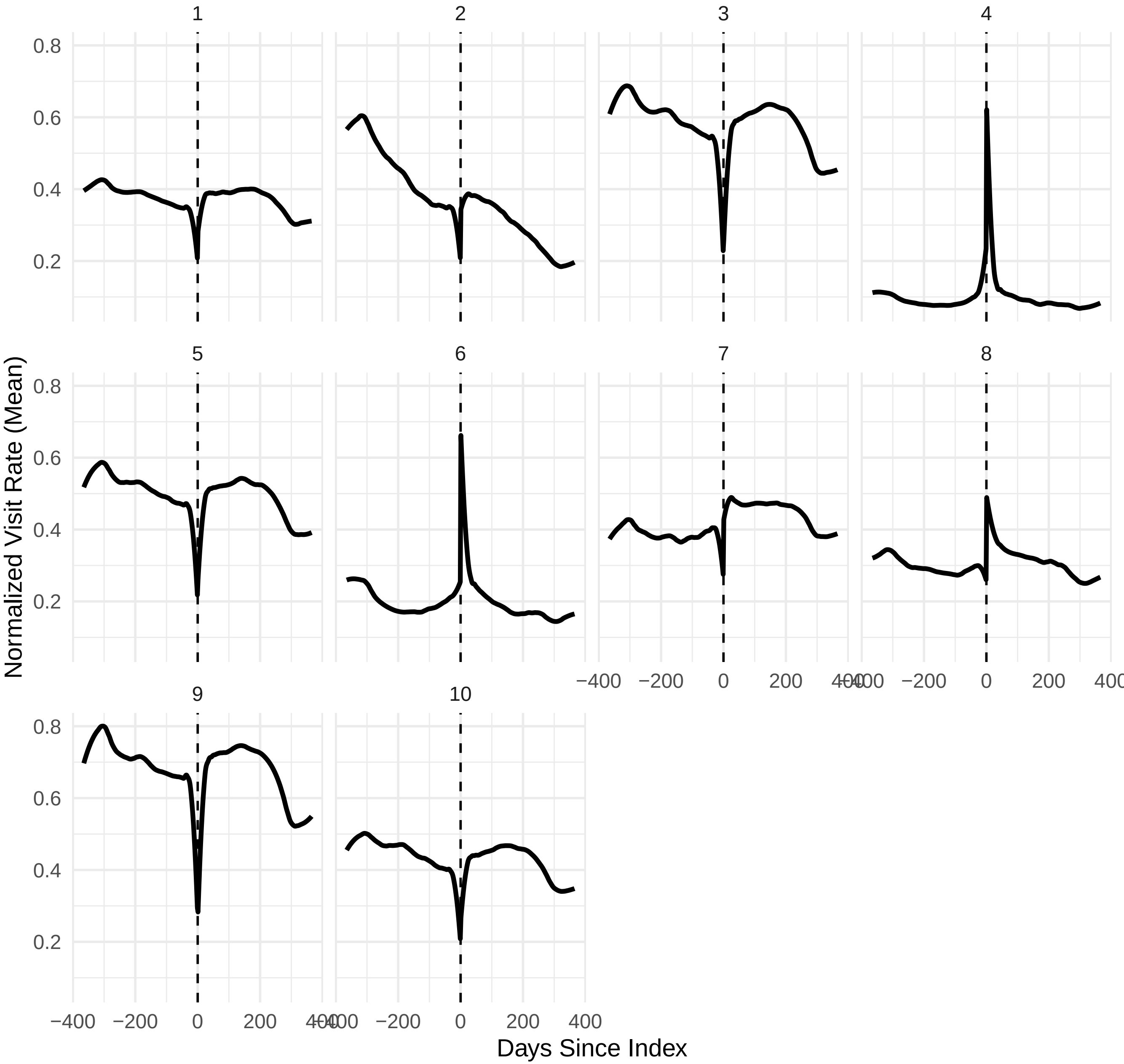

Figure A9: k = 11

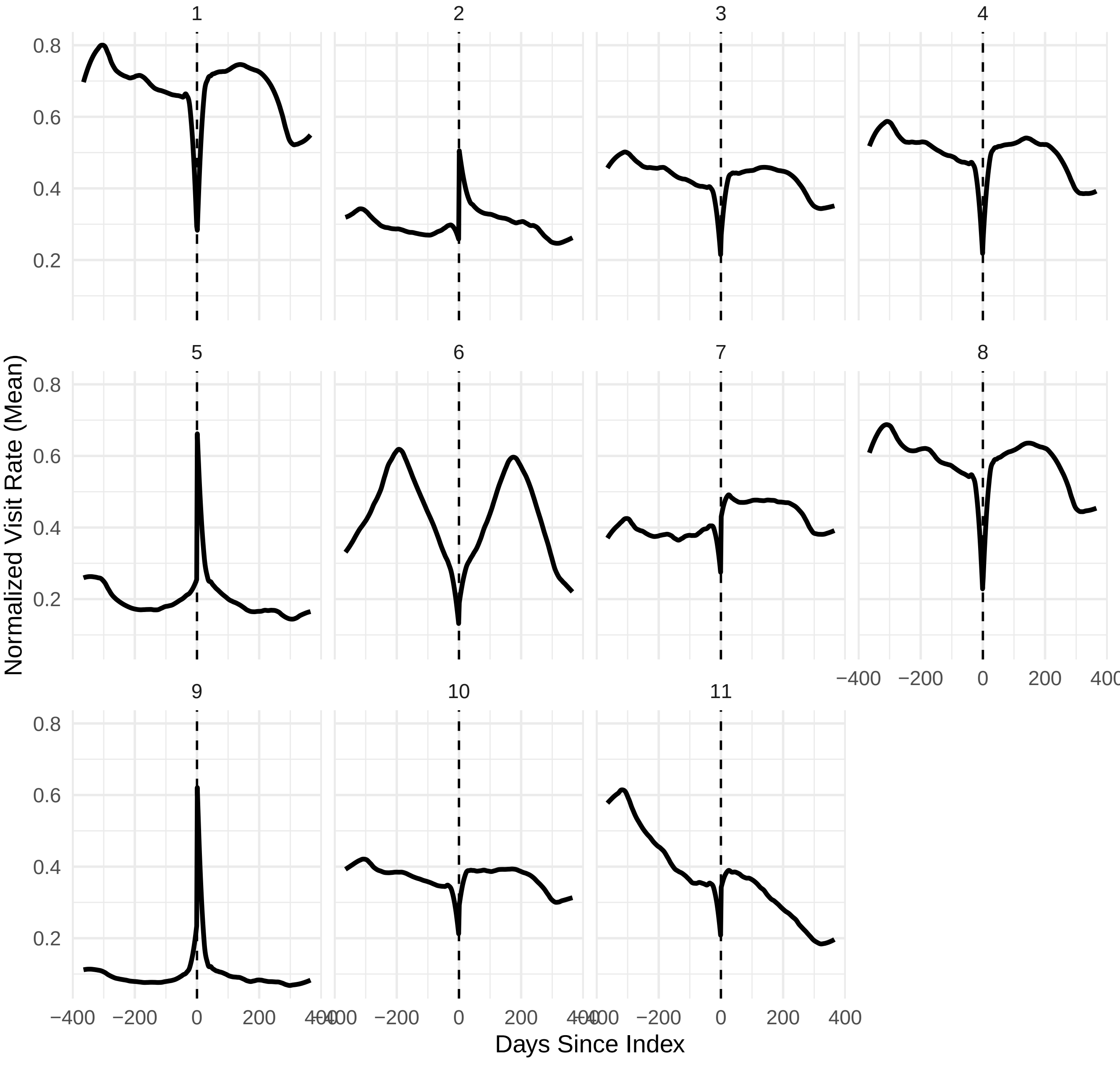

# Figure A10: k = 12

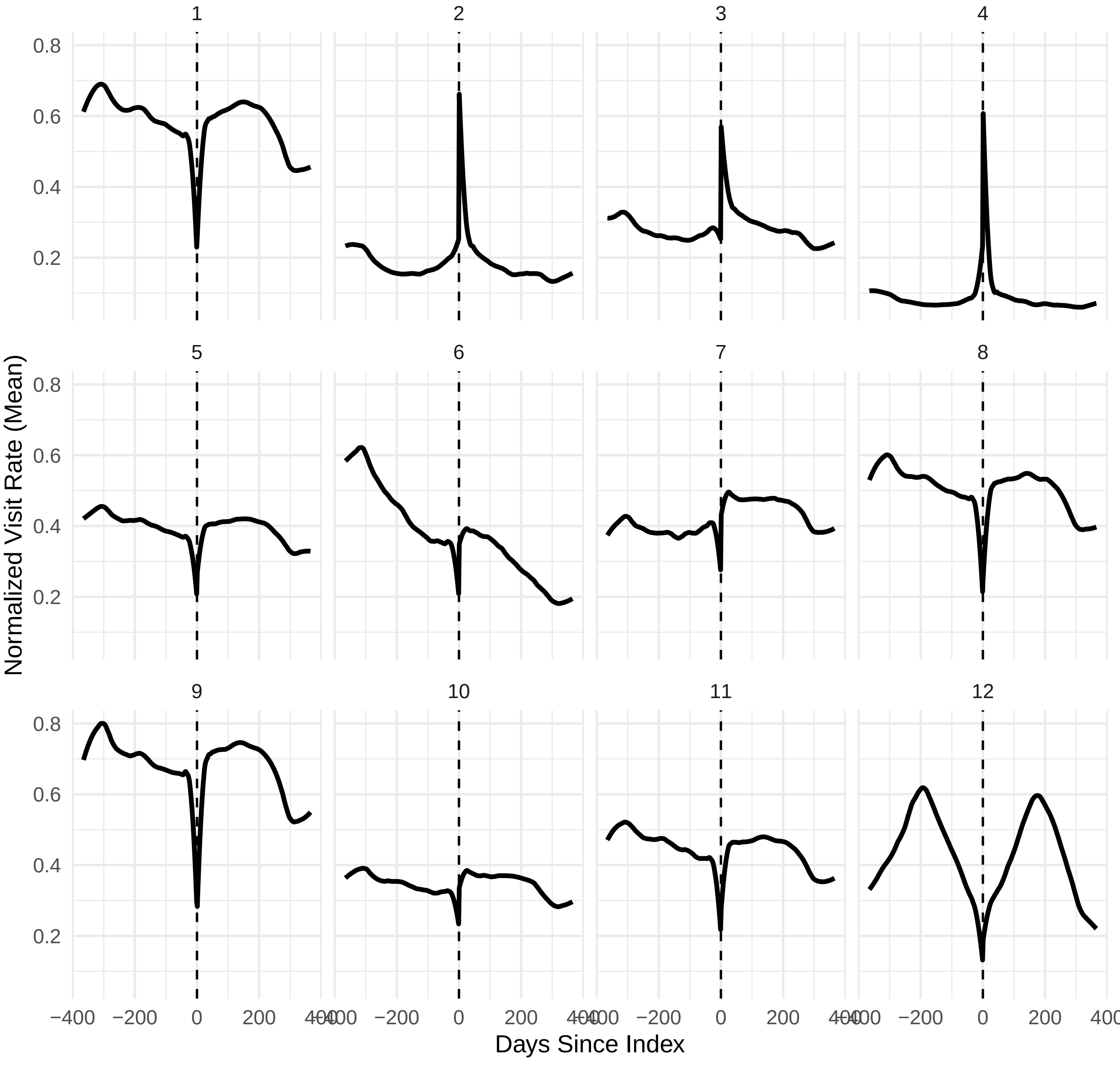

Figure A11: k = 13

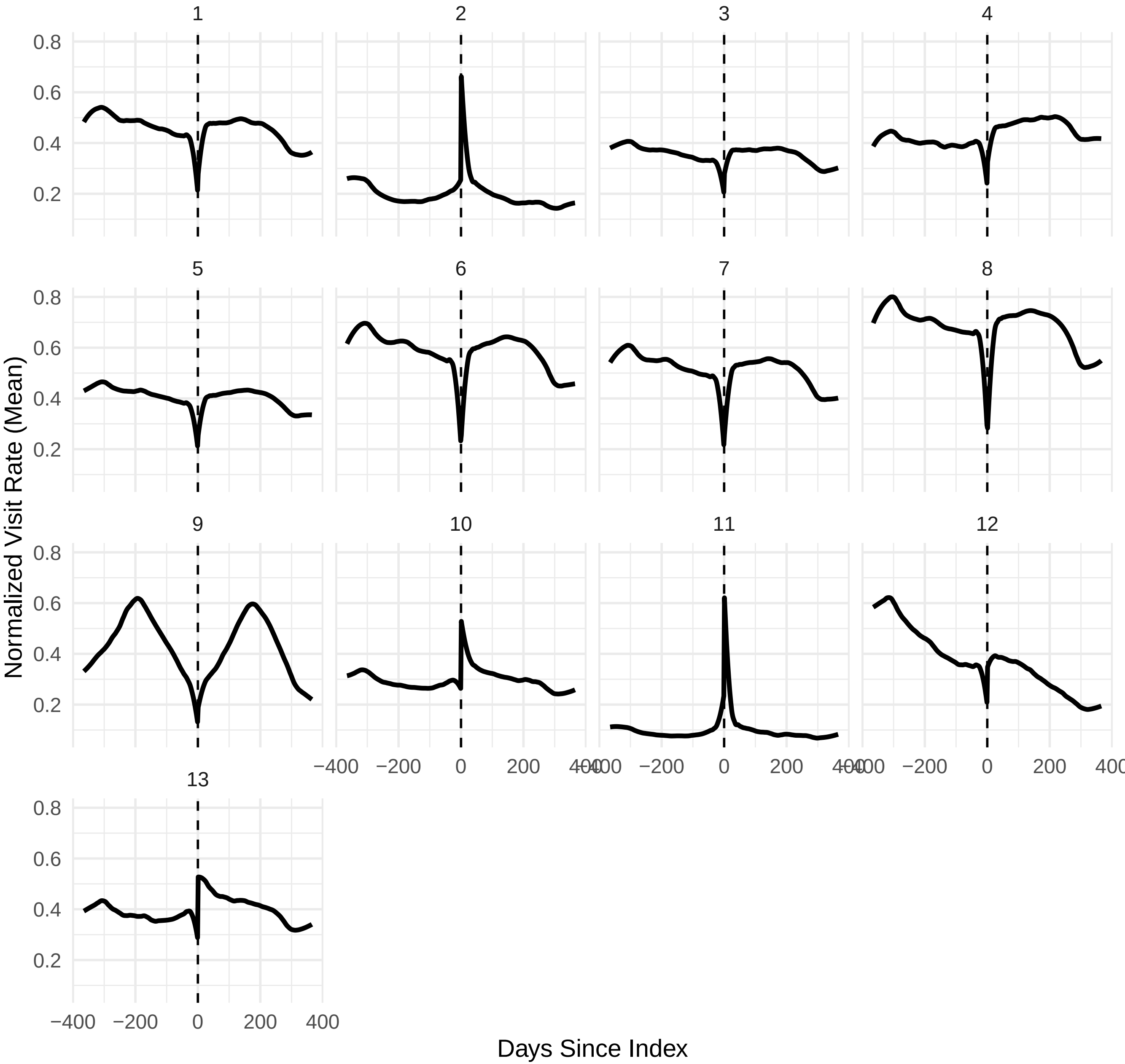

Figure A12: k = 14

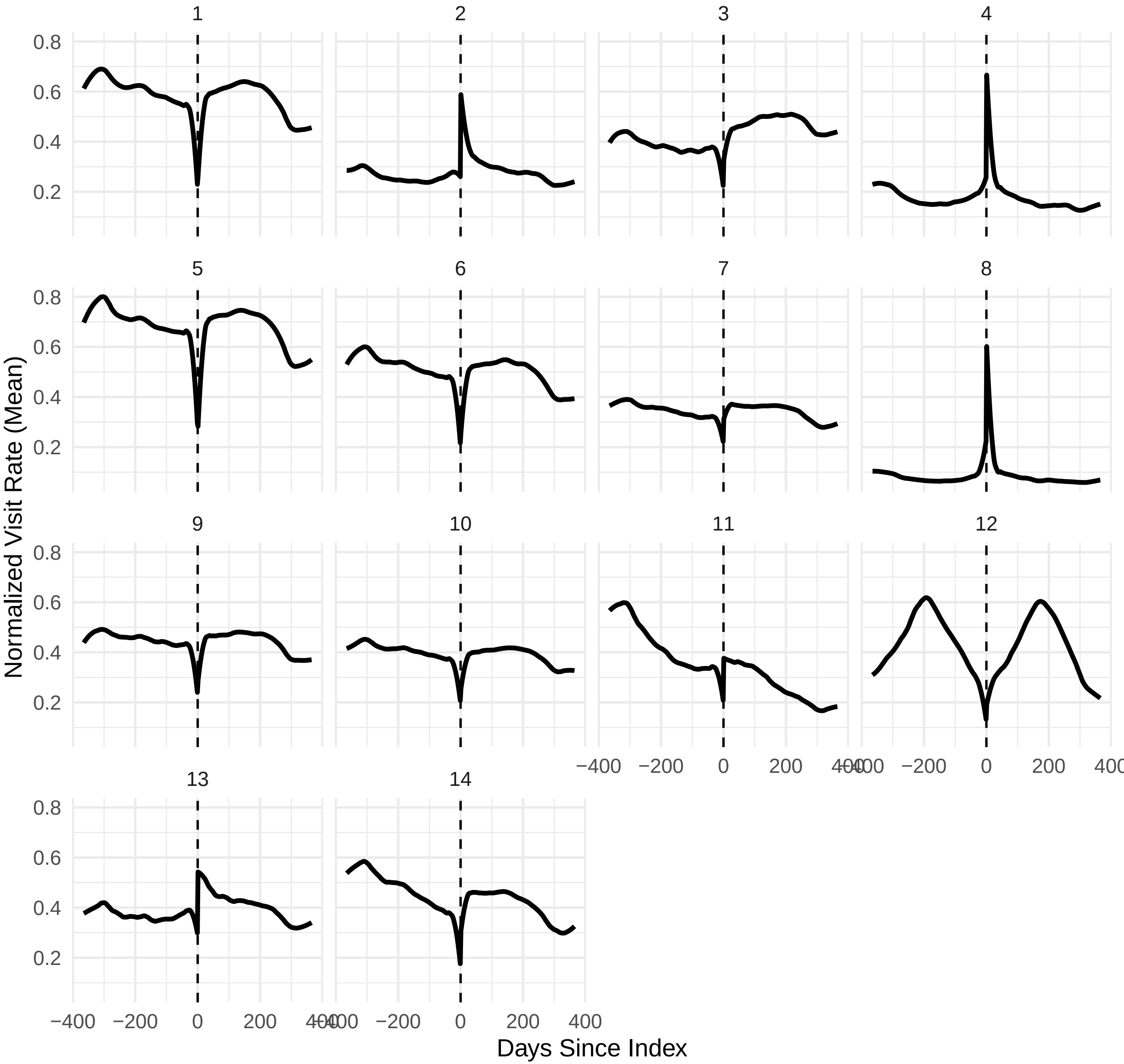

Figure A13: k = 15

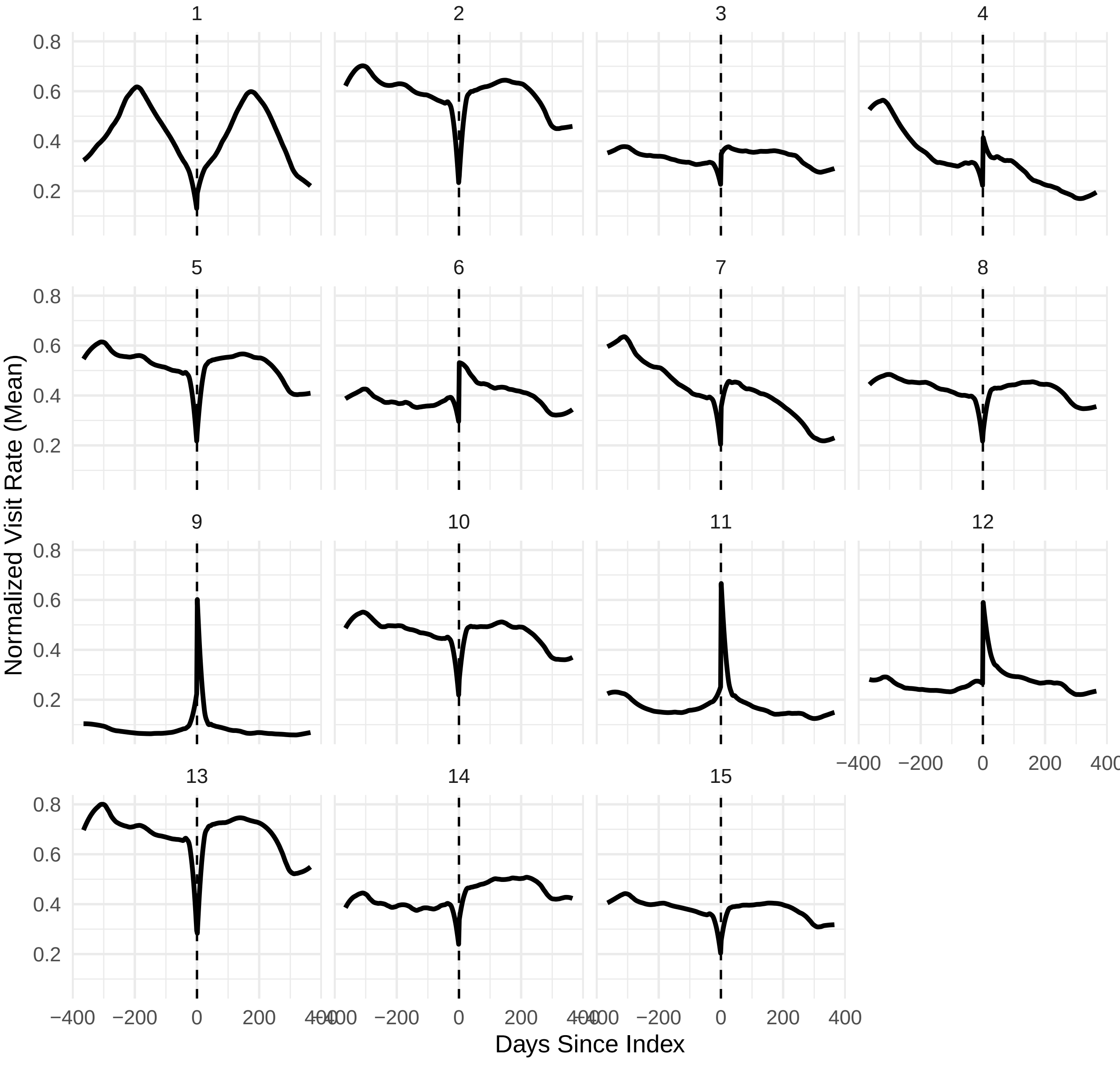

Figure A14: k = 16

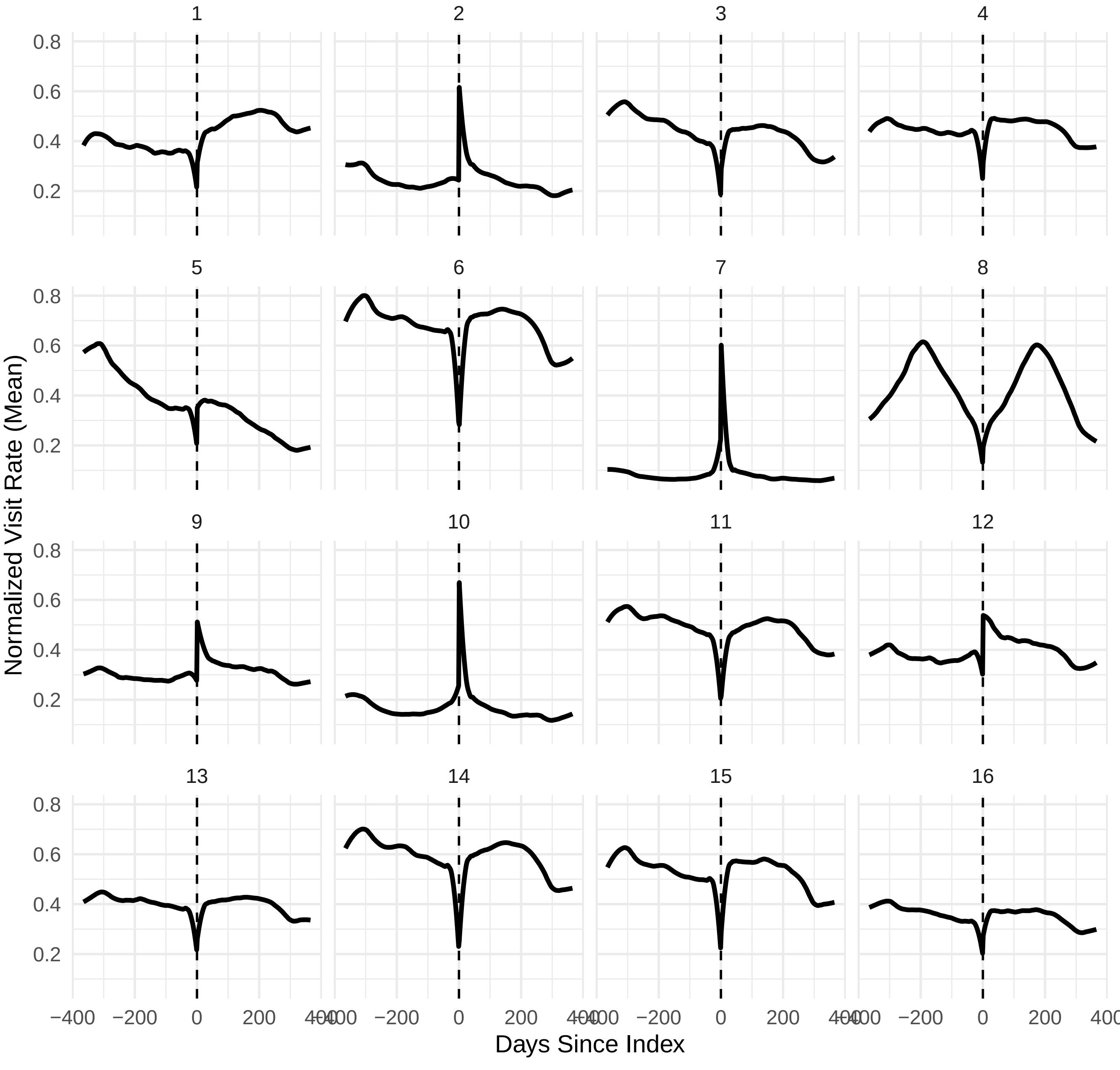

Figure A15: k = 17

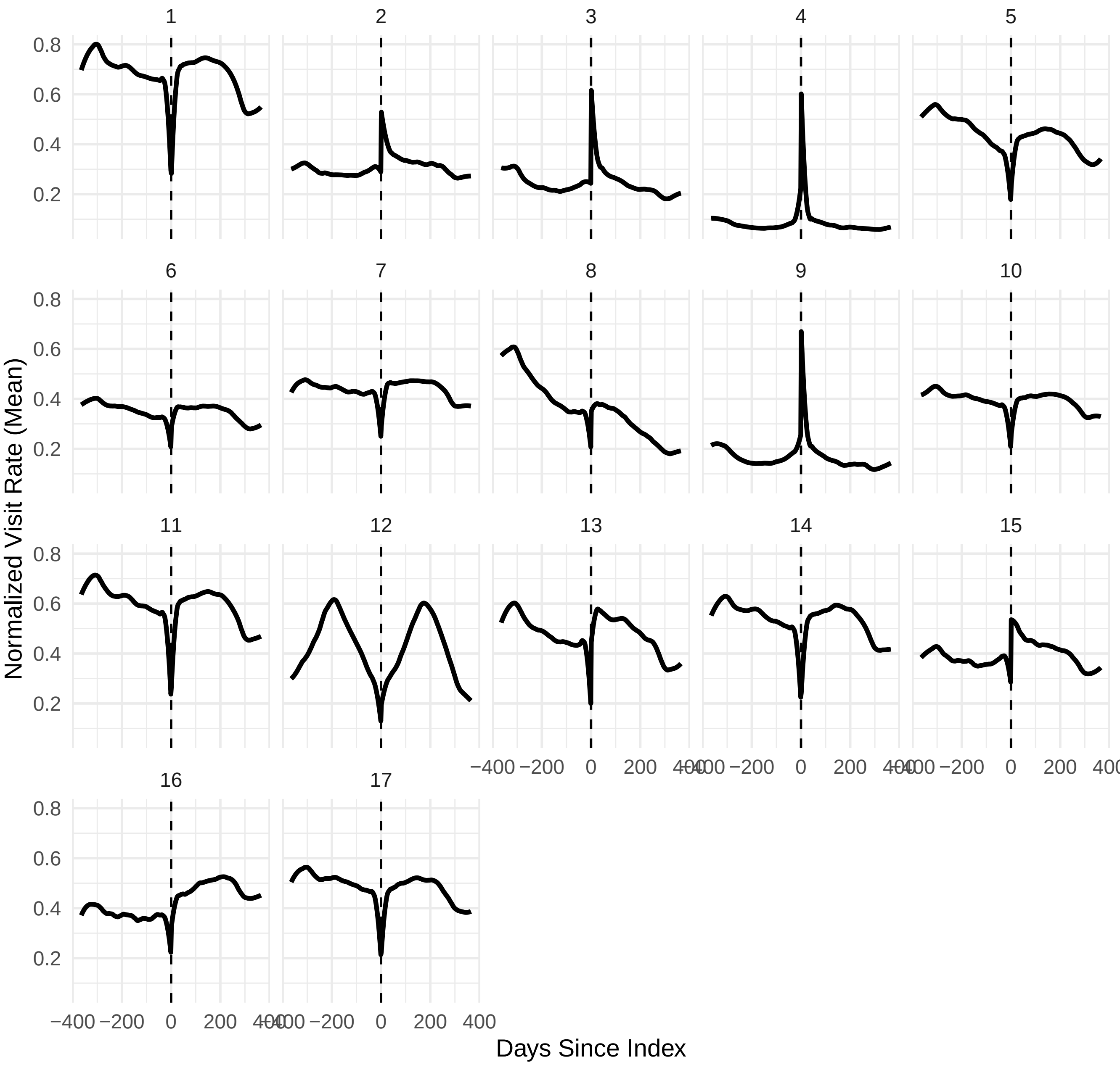

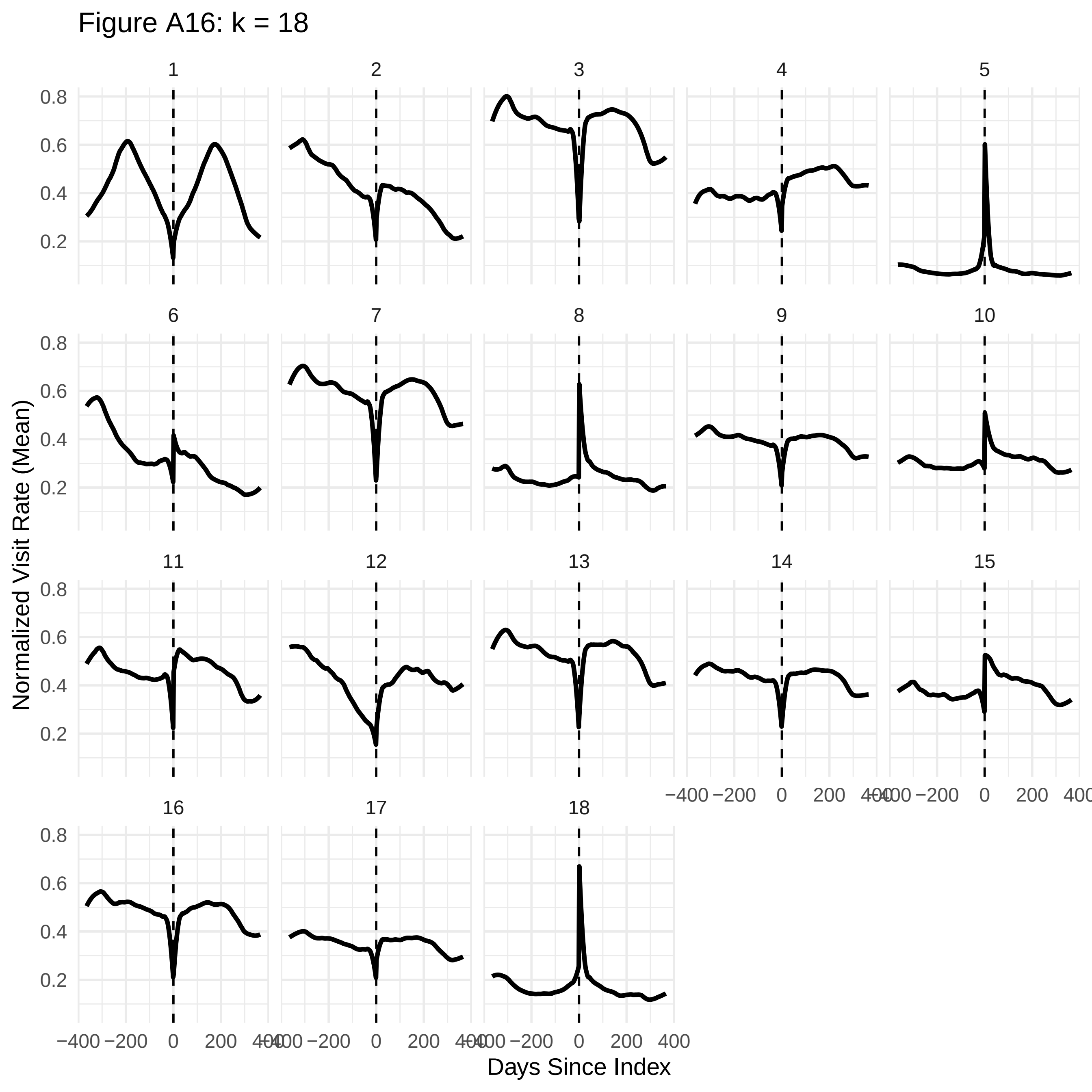
Figure A16: k = 18
1
2
3
4
5
6
7
8
9
10
11
12
13
14
15
16
17
18
0.8
0.6
0.4
0.2
−400 −200 0 200 400
Normalized Visit Rate (Mean)
Days Since Index

# Figure A17: k = 19

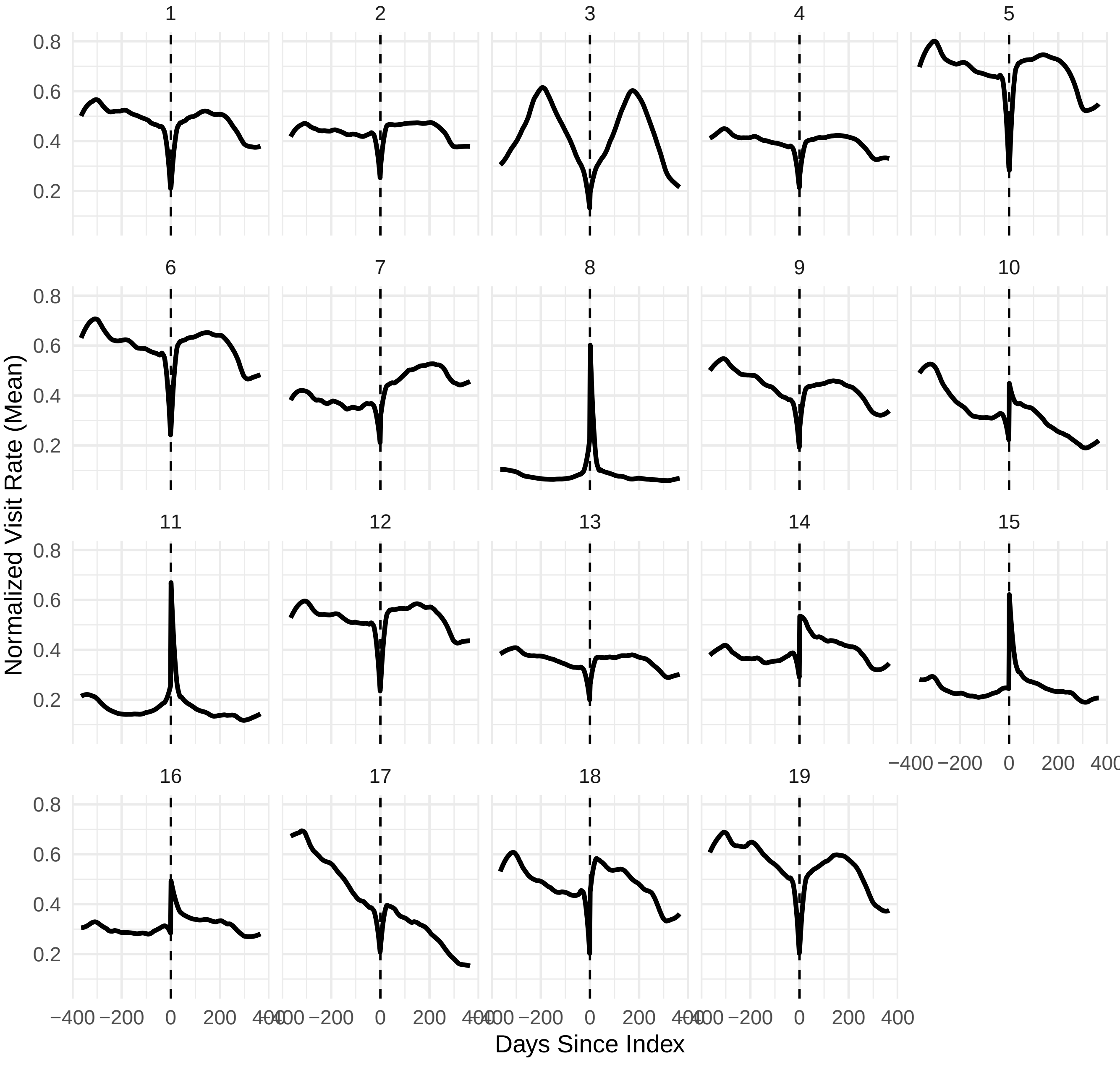

Figure A18: k = 20

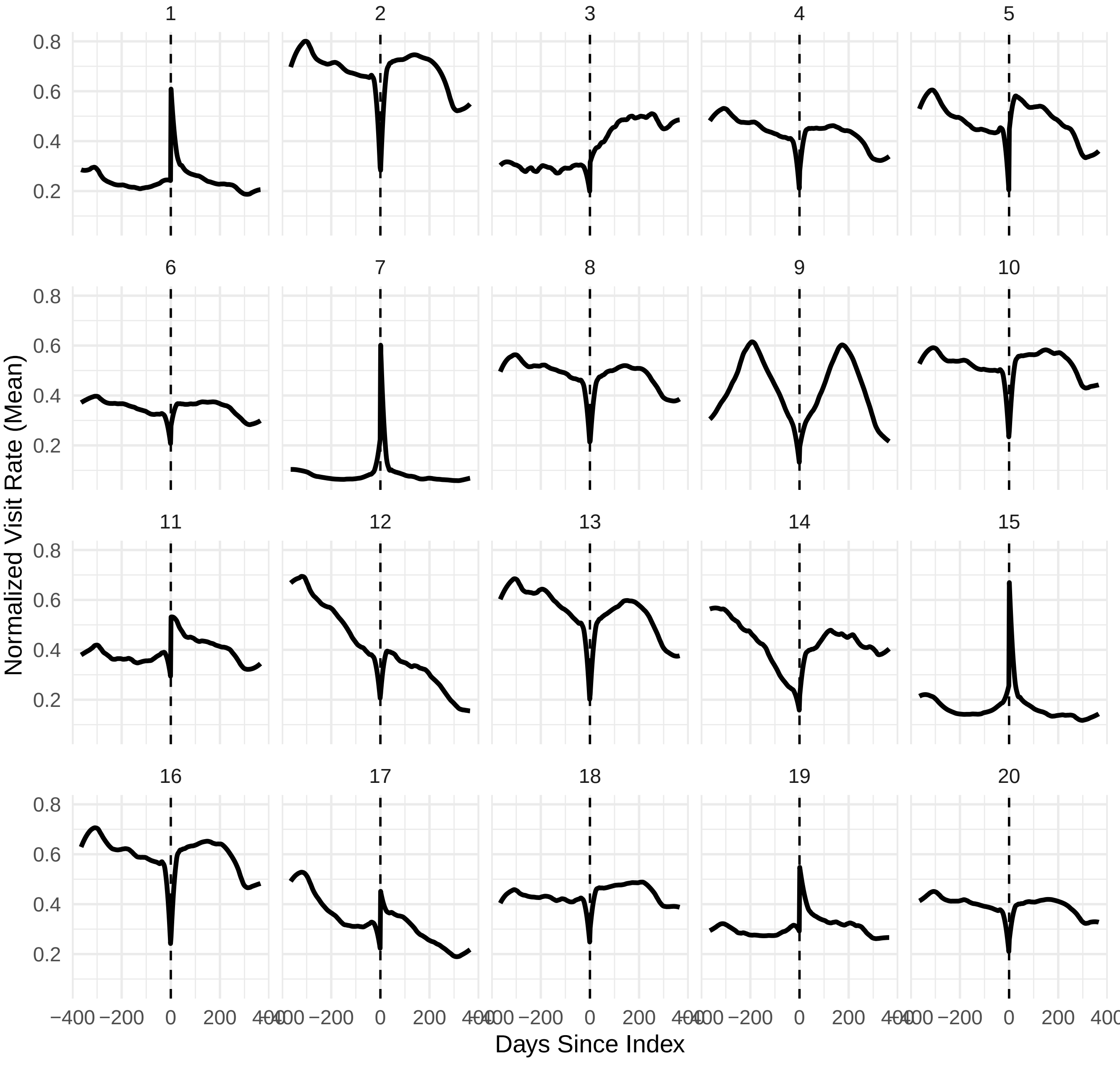

Figure A19: k = 21

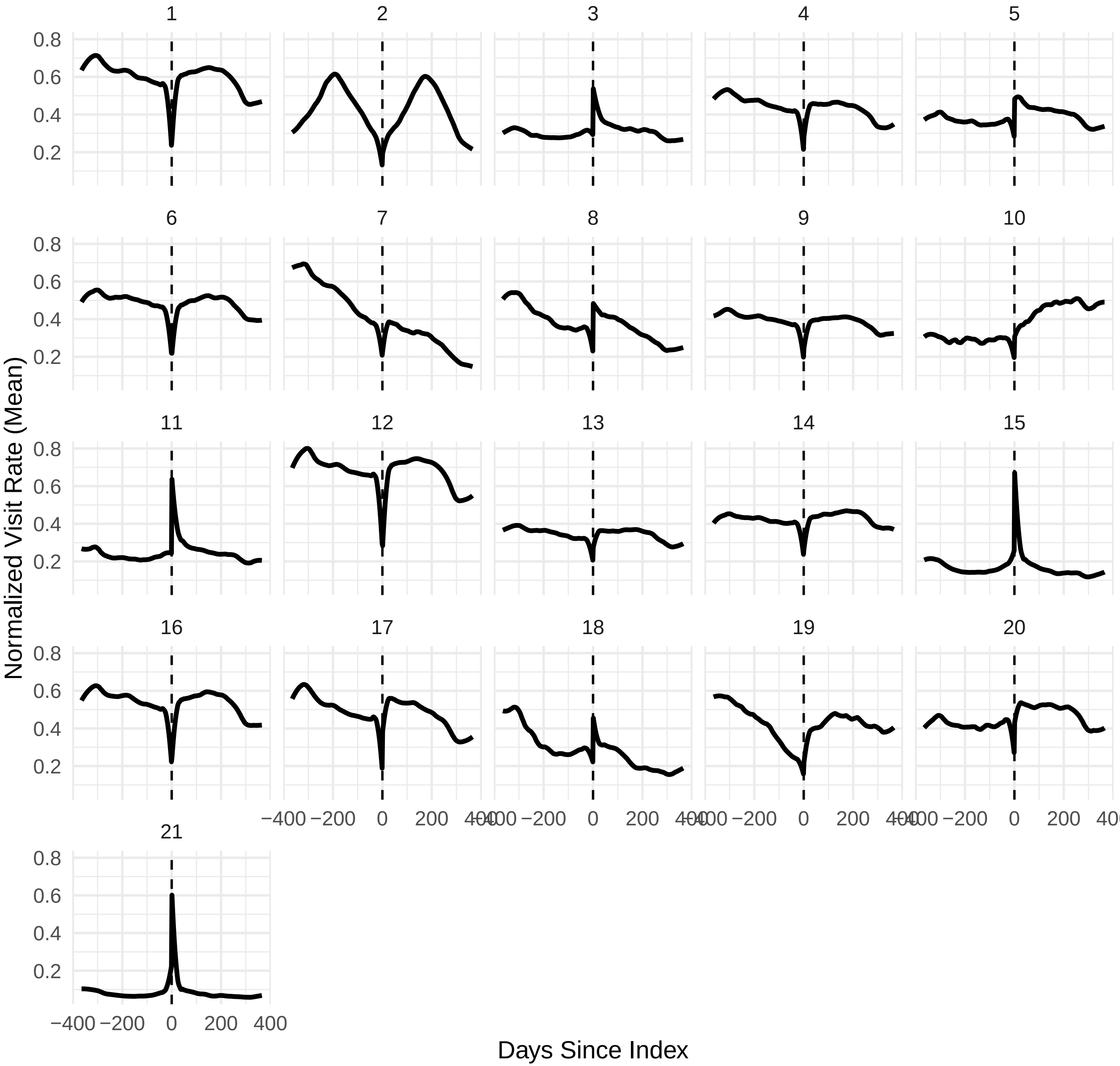

Figure A20: k = 22

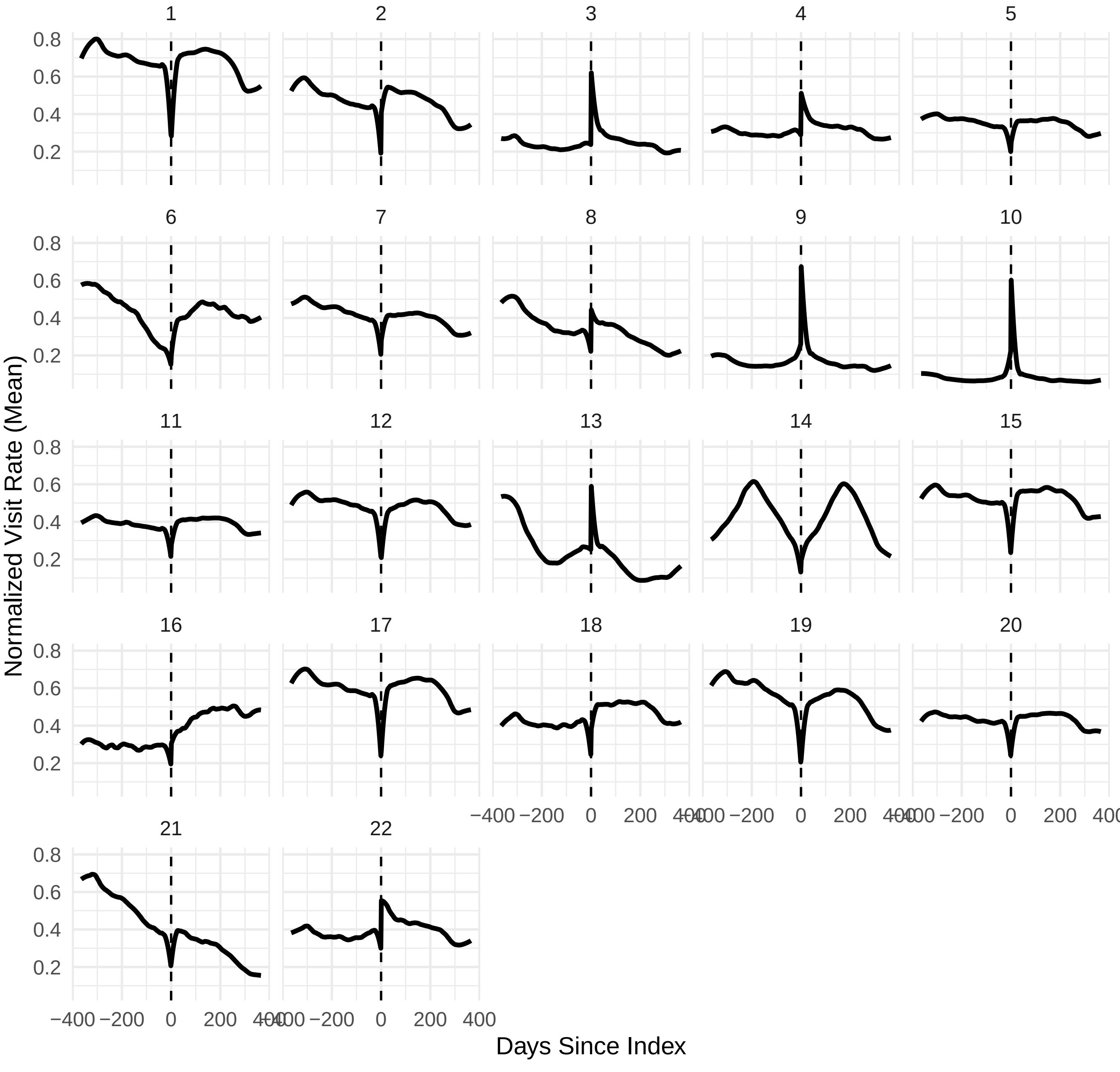

# Figure A21: k = 23

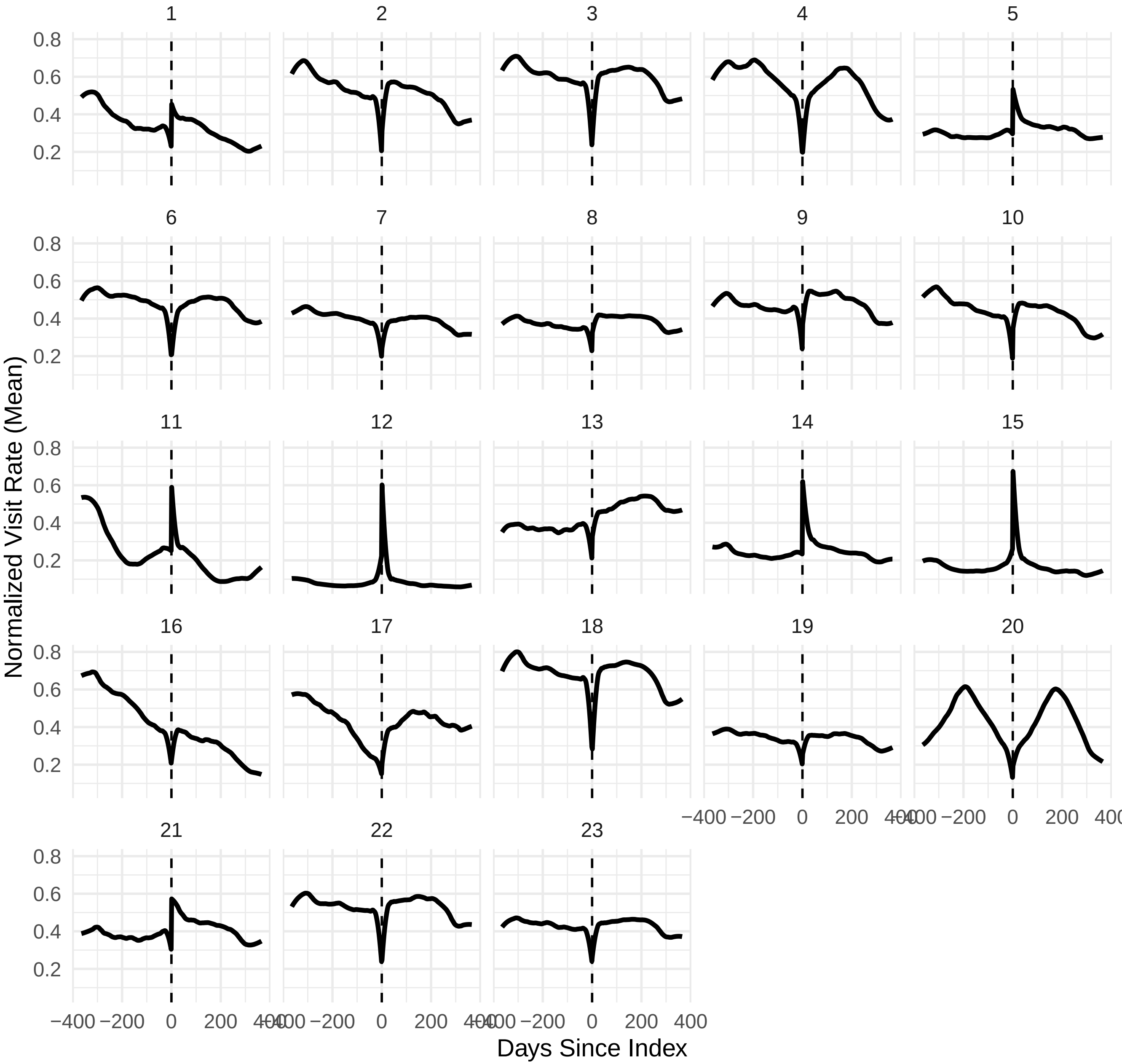

Figure A22: k = 24

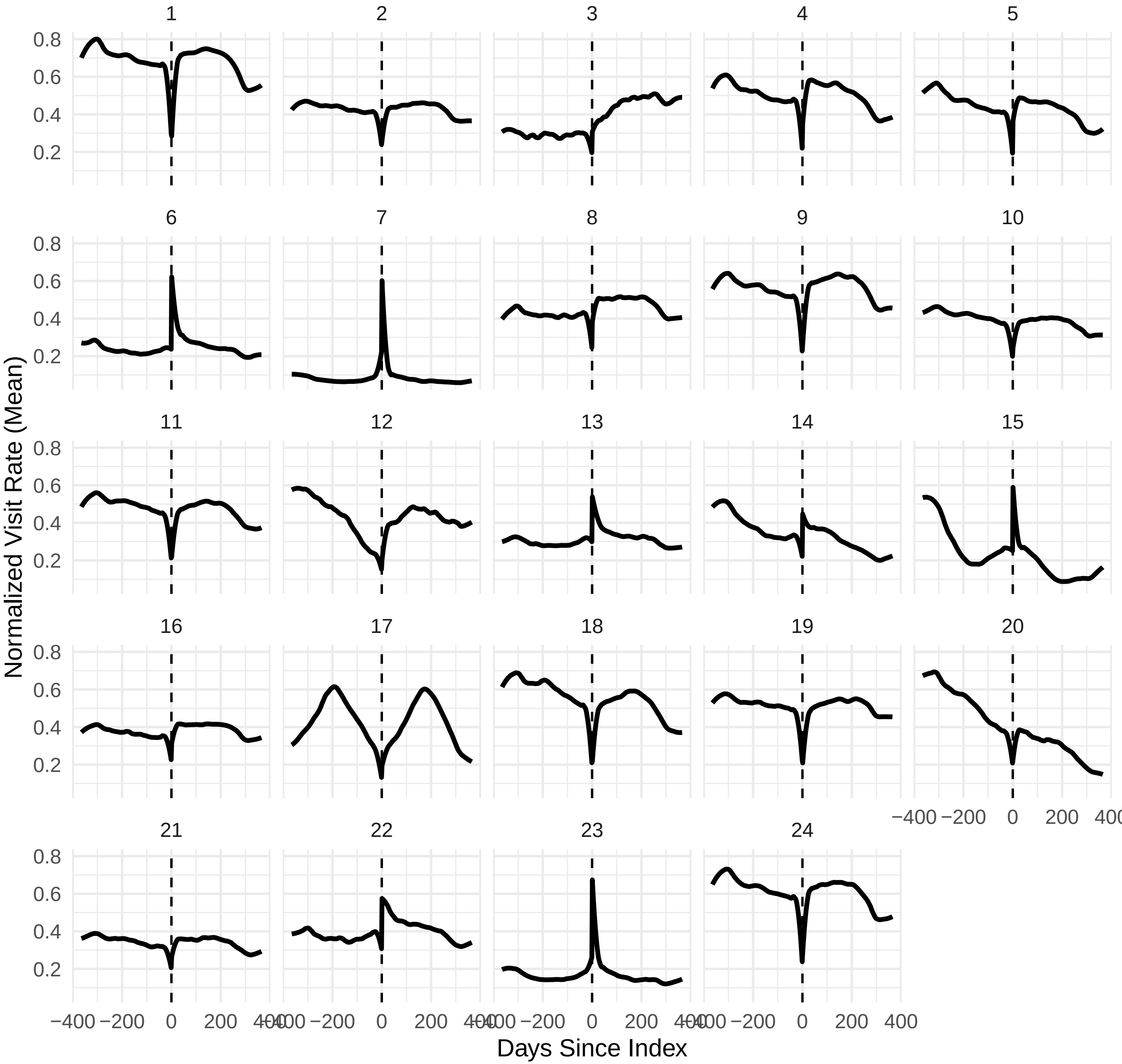

Figure A23: k = 25

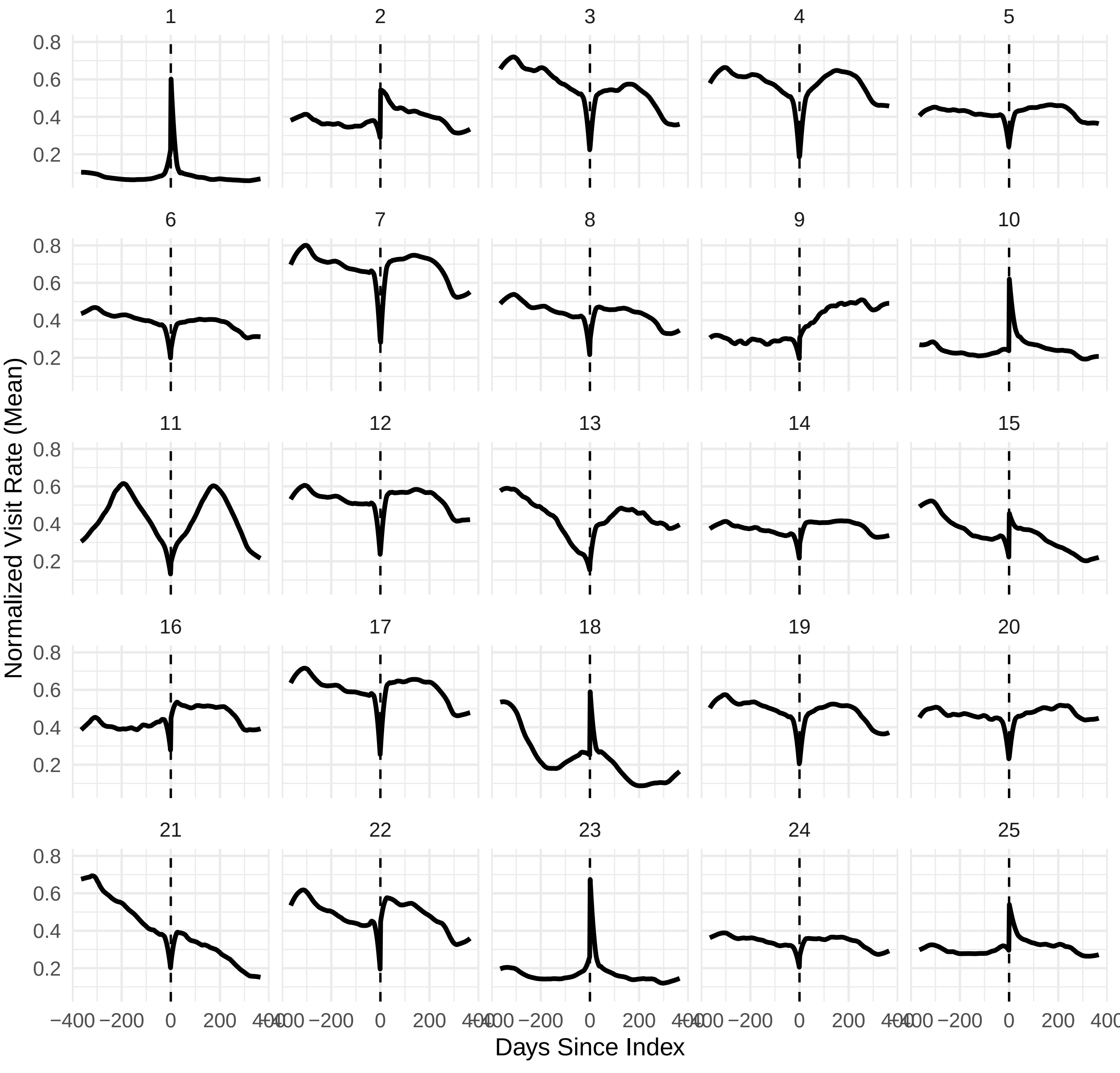

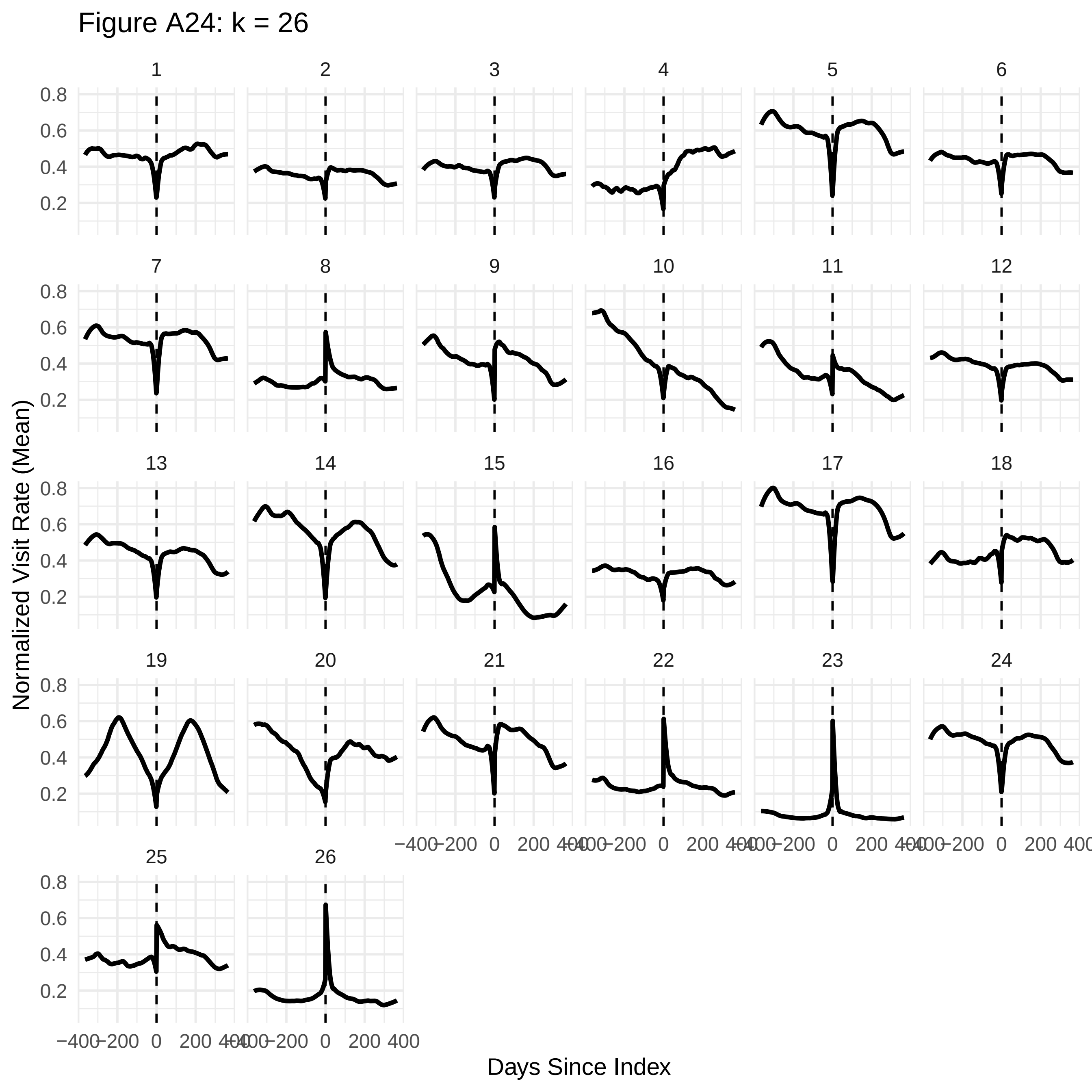
Figure A24: k = 26
1
2
3
4
5
6
7
8
9
10
11
12
13
14
15
16
17
18
19
20
21
22
23
24
25
26
0.8
0.6
0.4
0.2
−400
−200
0
200
400
Normalized Visit Rate (Mean)
Days Since Index

Figure A25: k = 27

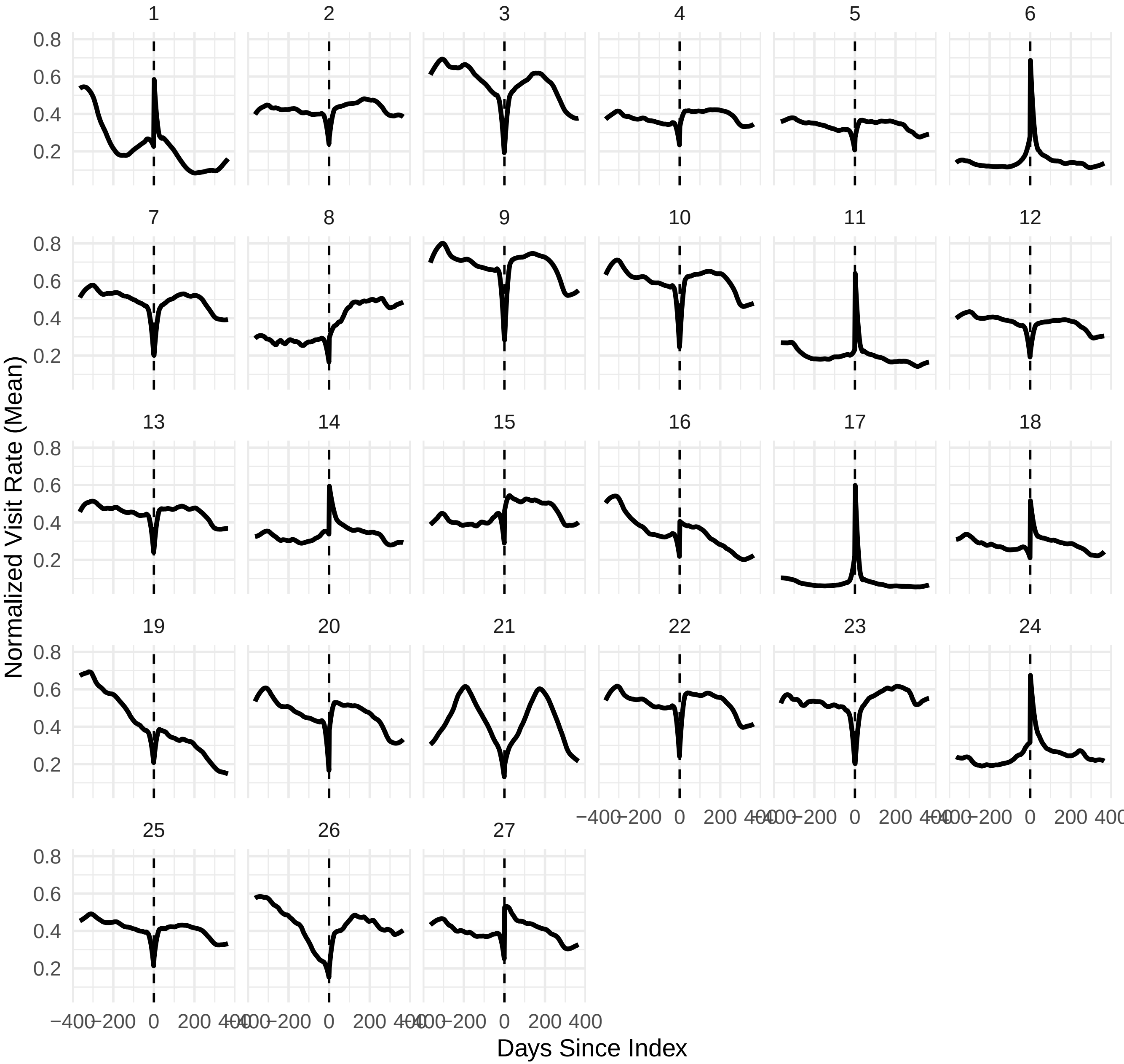

Figure A26: k = 28

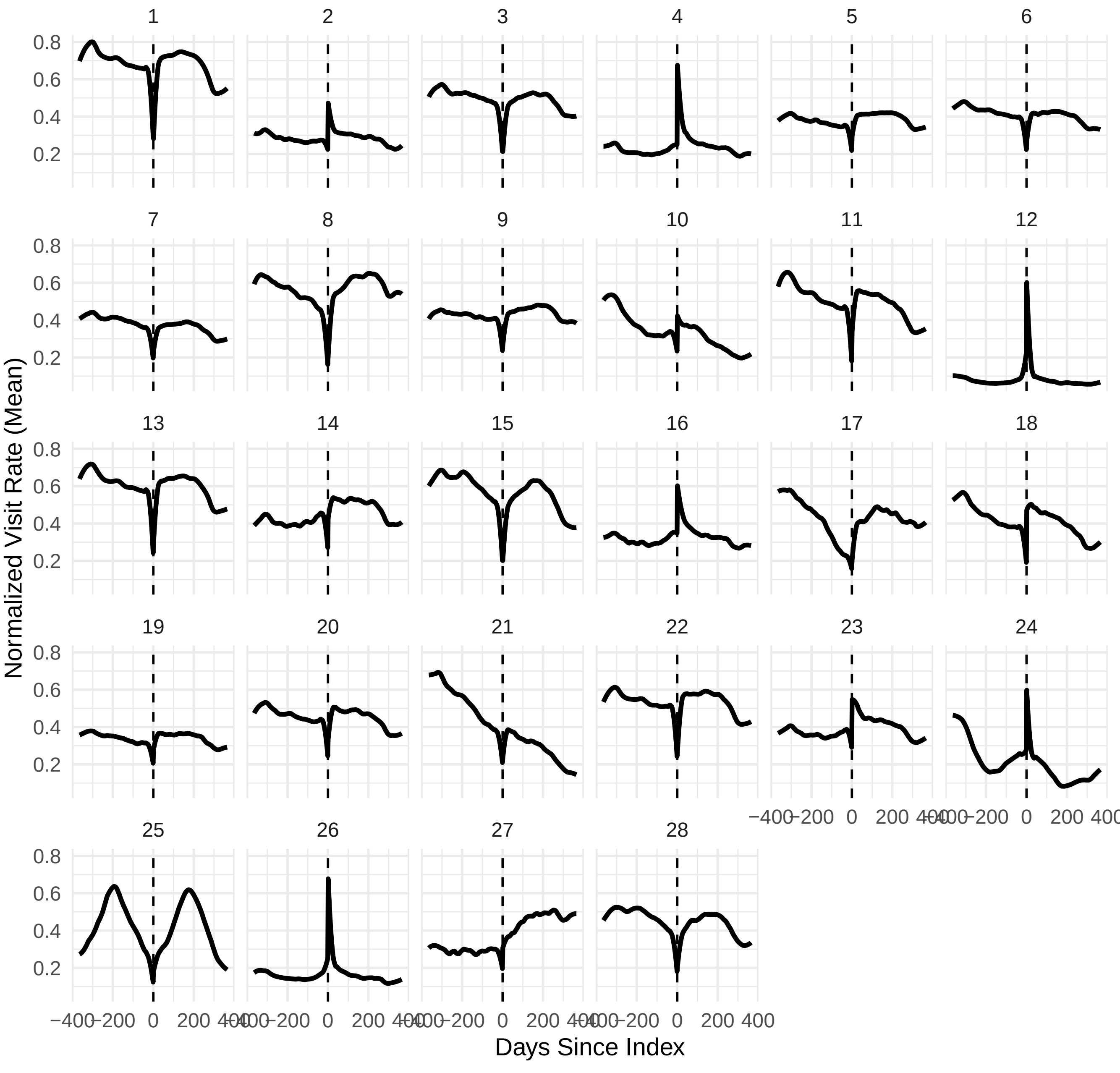

Figure A27: k = 29

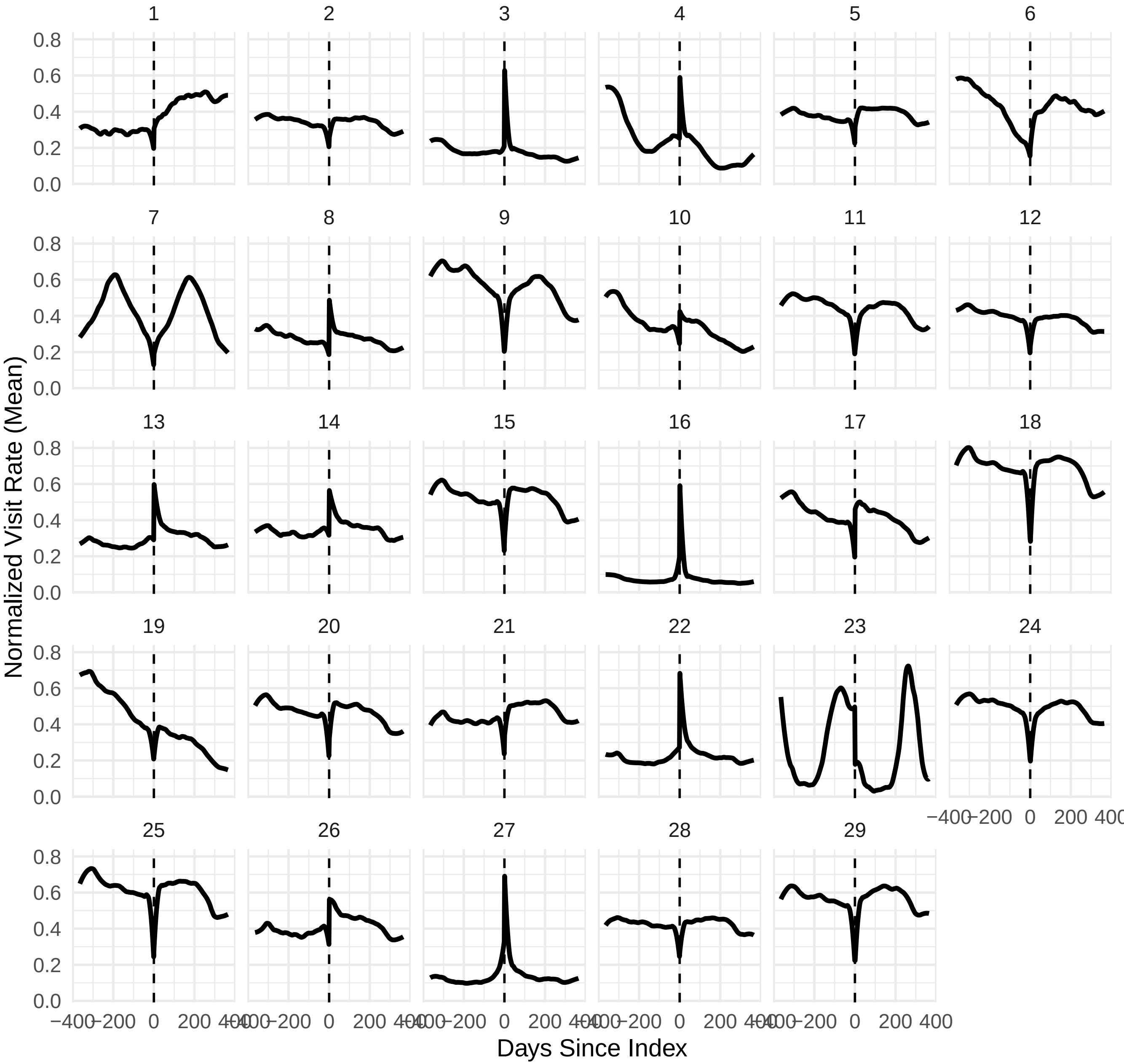

Figure A28: k = 30

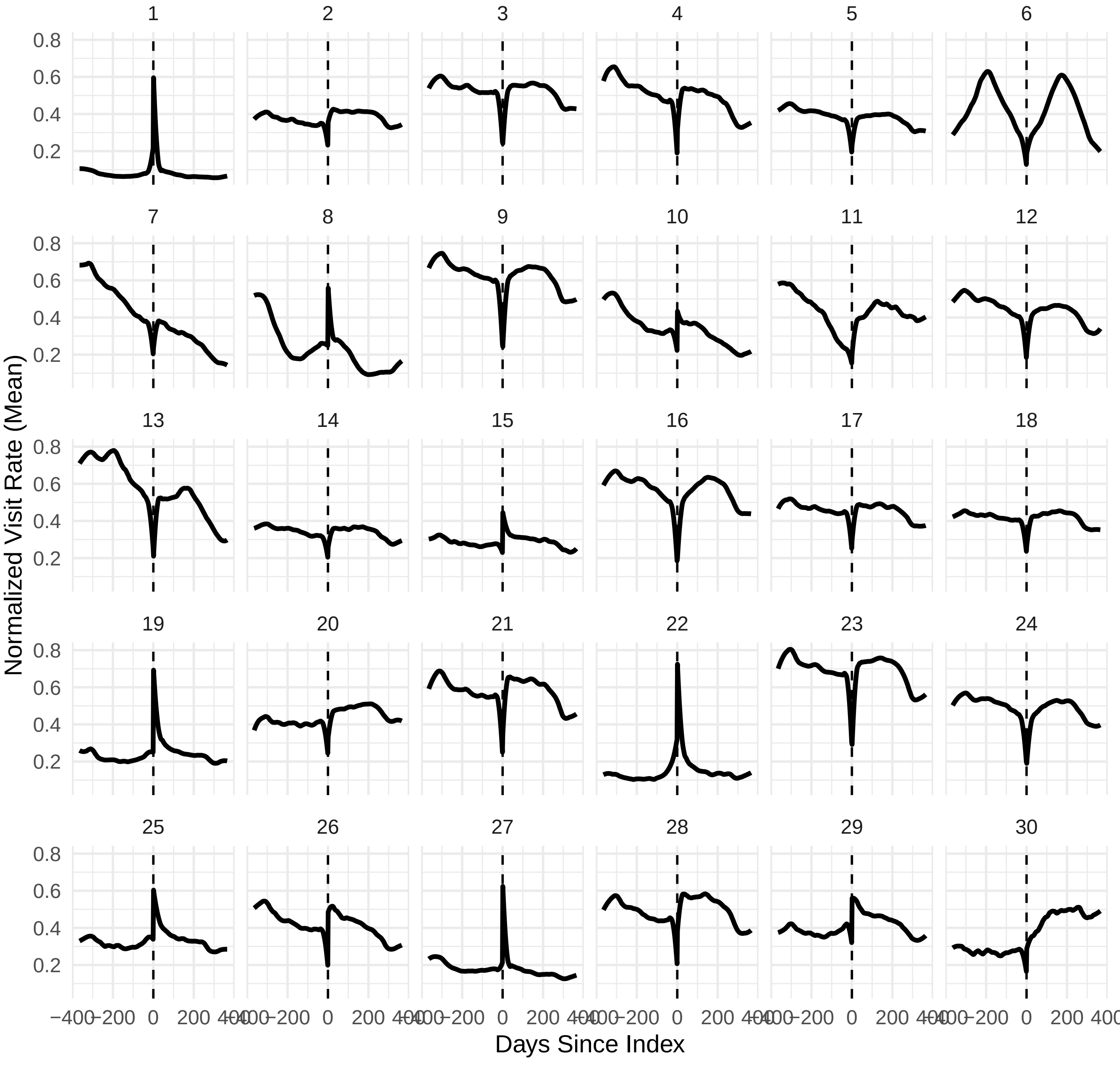

Supplement: Supplementary file 1 [file ofag629_supplementary_data.zip › FigA1_A28.pdf]
