# Supplementary material for: Estimating the Range and Duration of Post-influenza Symptoms, Conditions, and Diseases
Source: Open Forum Infect Dis. 2026 Oct 6;13(10):ofag629. doi: 10.1093/ofid/ofag629 (PMC13660689; doi:10.1093/ofid/ofag629)

Figure B1: k = 3

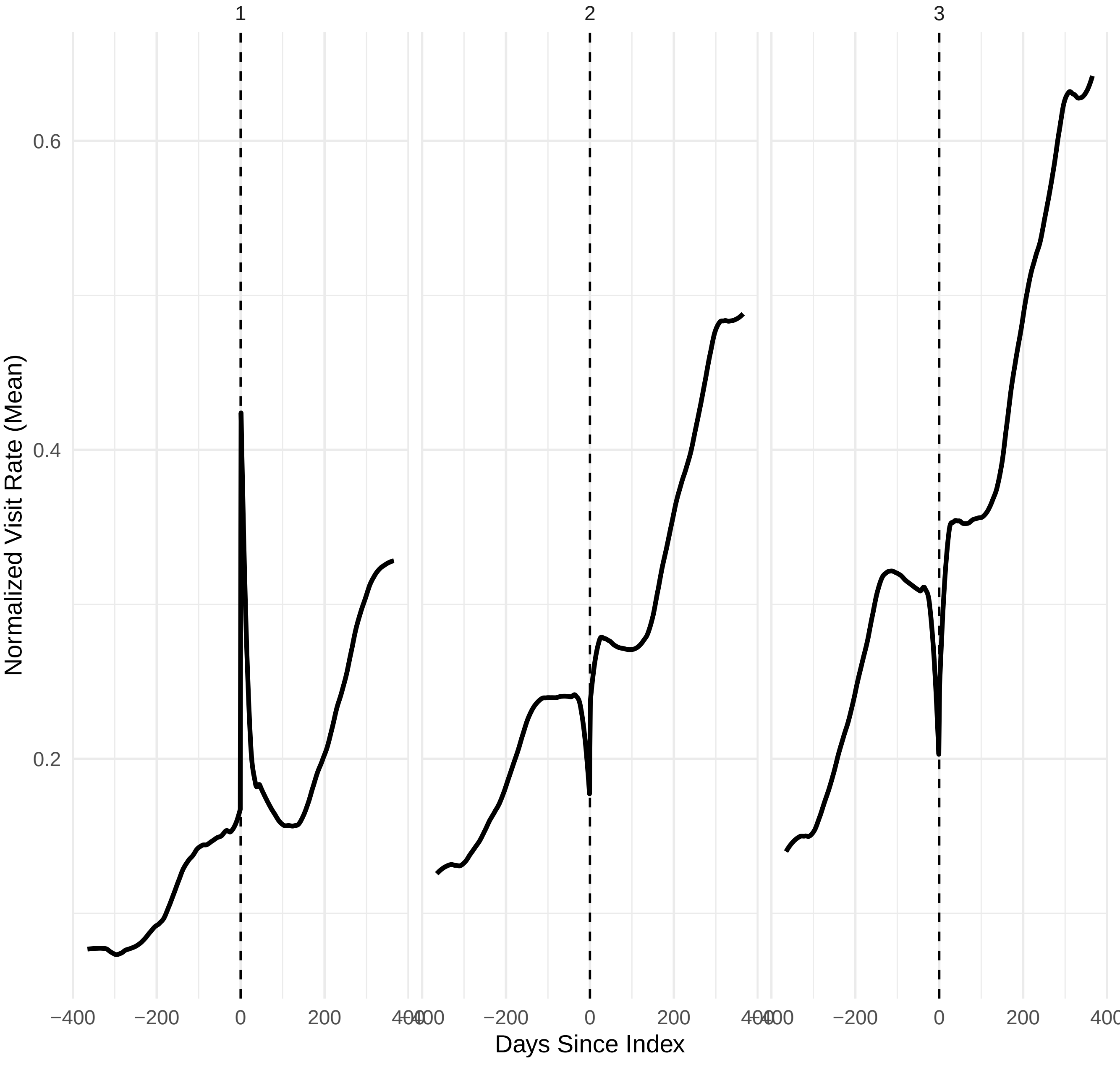
1
2
3
0.6
0.4
0.2
Normalized Visit Rate (Mean)
−400
−200
0
200
400
Days Since Index

Figure B2: k = 4

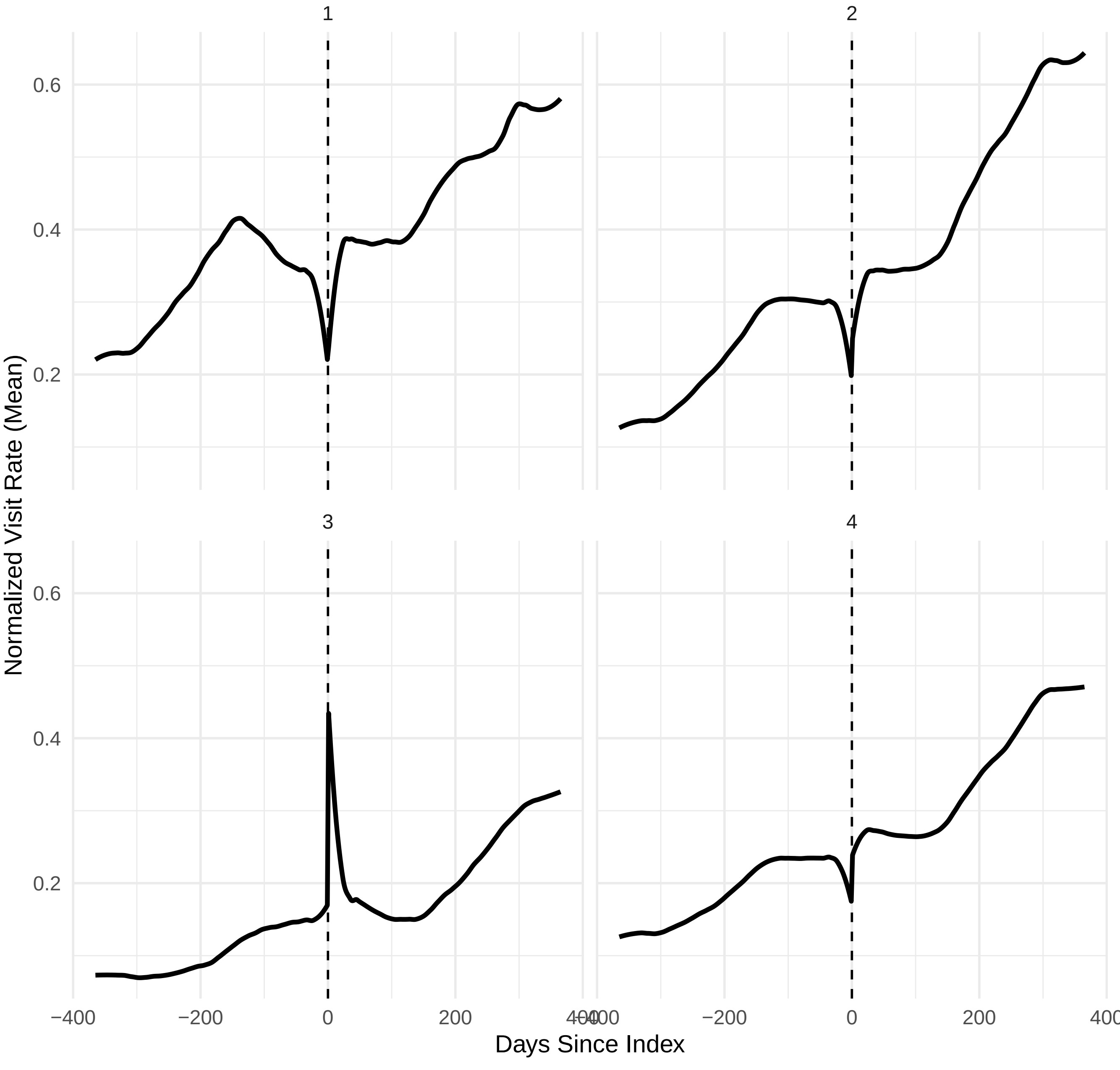
1
2
3
4
0.6
0.4
0.2
−400
−200
0
200
400
Normalized Visit Rate (Mean)
Days Since Index

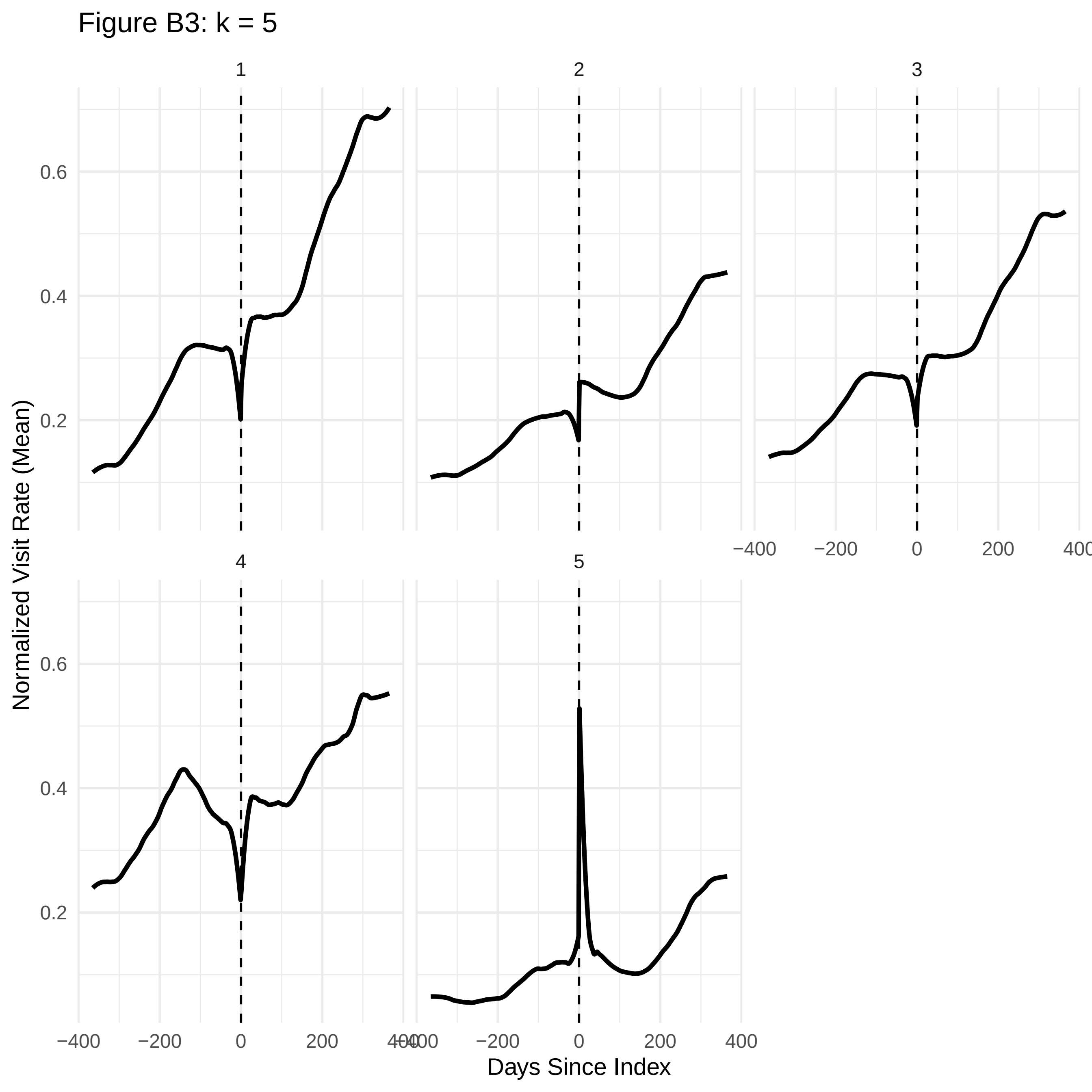

Figure B3: k = 5
1
2
3
4
5
0.6
0.4
0.2
−400
−200
0
200
400
Normalized Visit Rate (Mean)
Days Since Index

Figure B4: k = 6

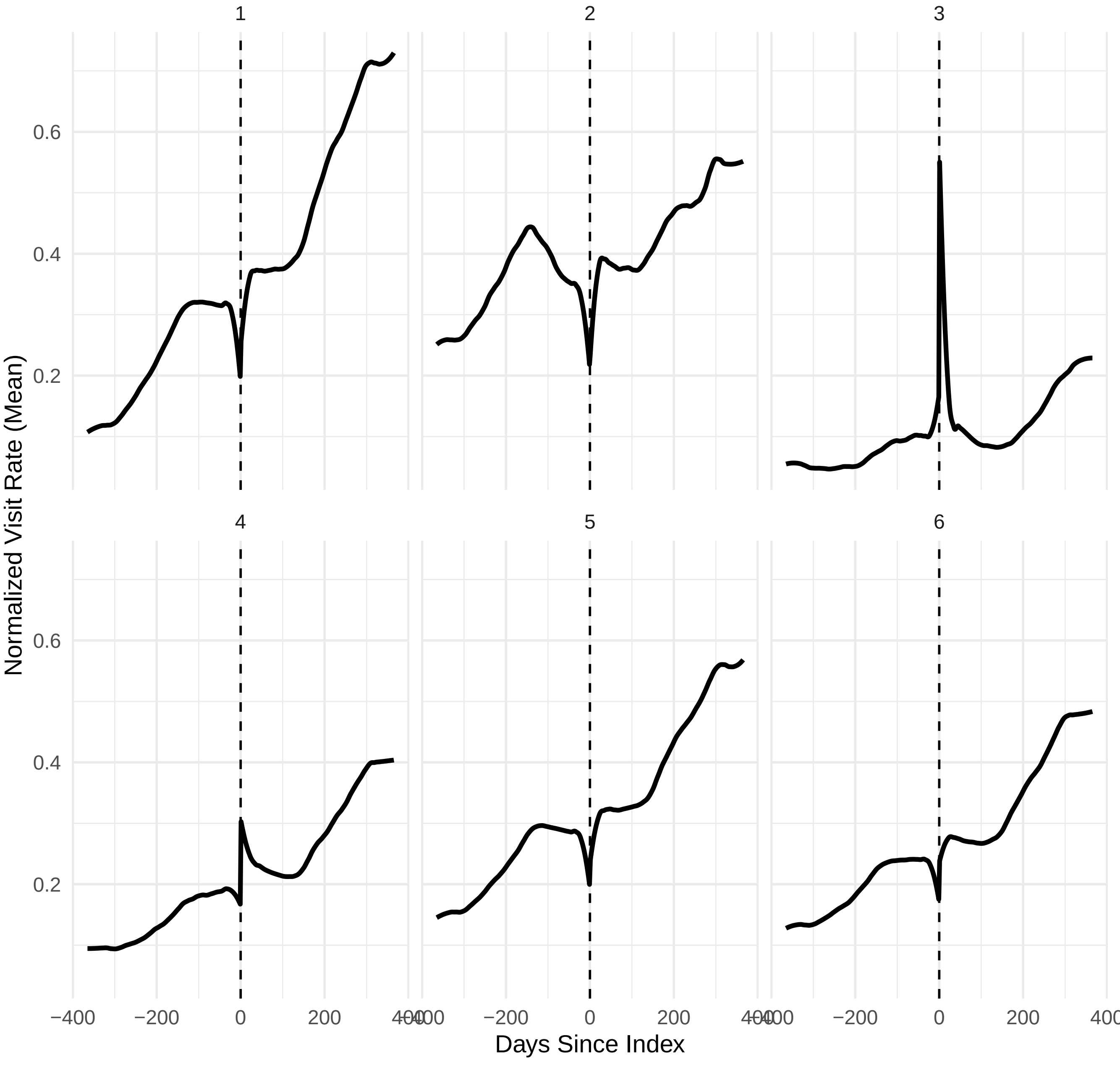
1
2
3
4
5
6
Normalized Visit Rate (Mean)
0.2
0.4
0.6
−400
−200
0
200
400
Days Since Index

Figure B5: k = 7

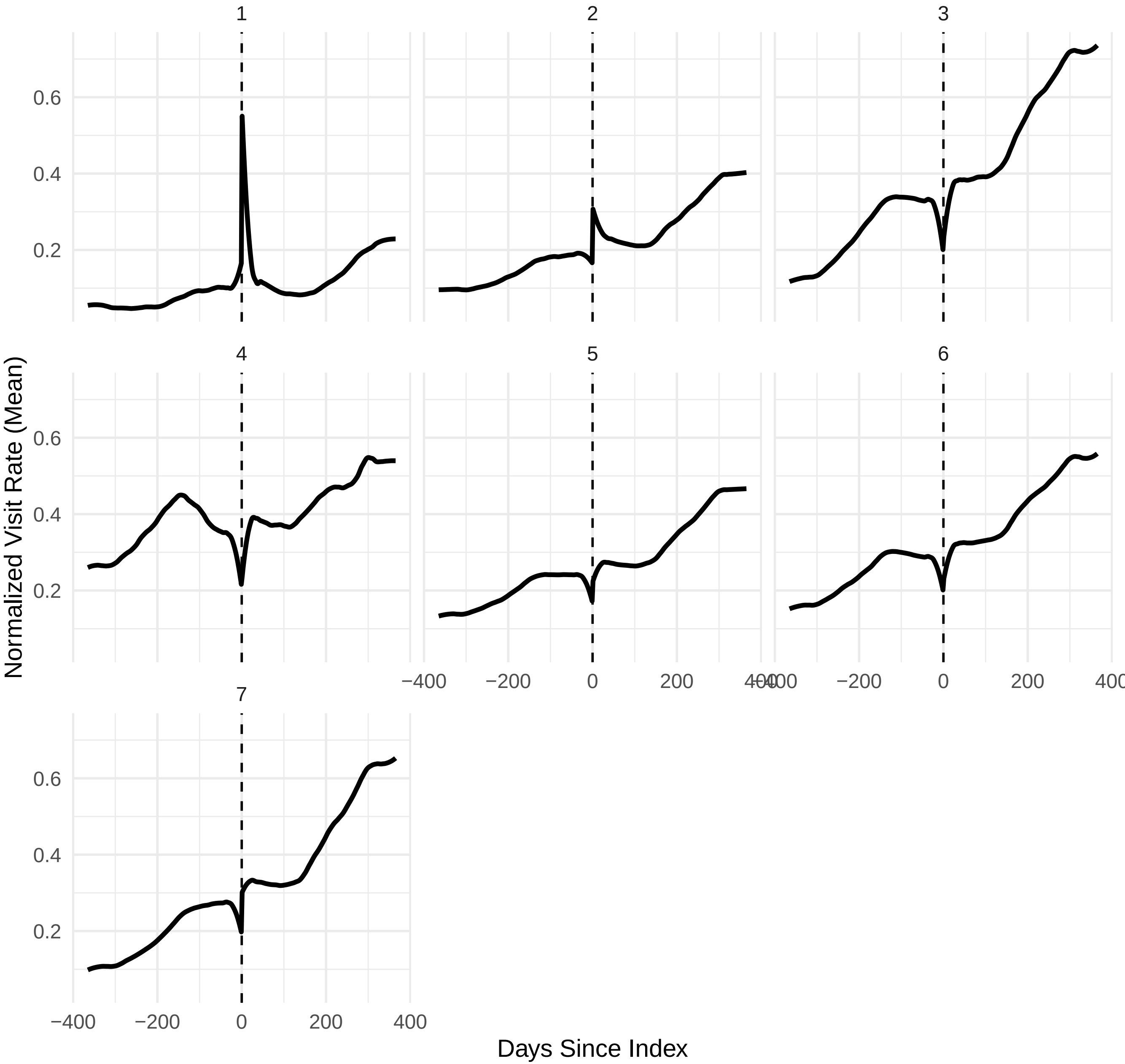

1
2
3
4
5
6
7
Normalized Visit Rate (Mean)
0.6
0.4
0.2
−400
−200
0
200
400
Days Since Index

Figure B6: k = 8

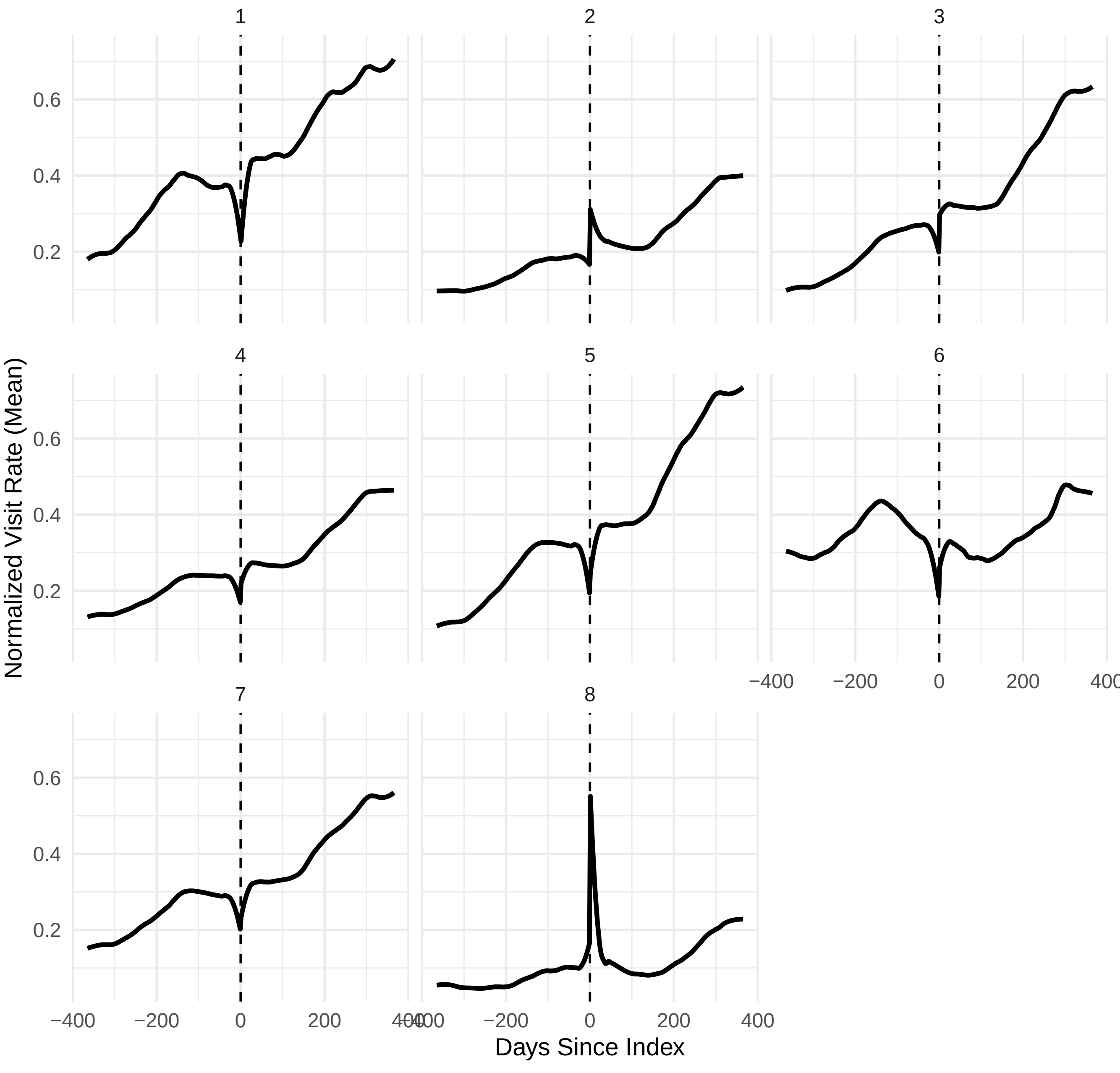
1
2
3
4
5
6
7
8
0.6
0.4
0.2
Normalized Visit Rate (Mean)
−400
−200
0
200
400
Days Since Index

Figure B7: k = 9

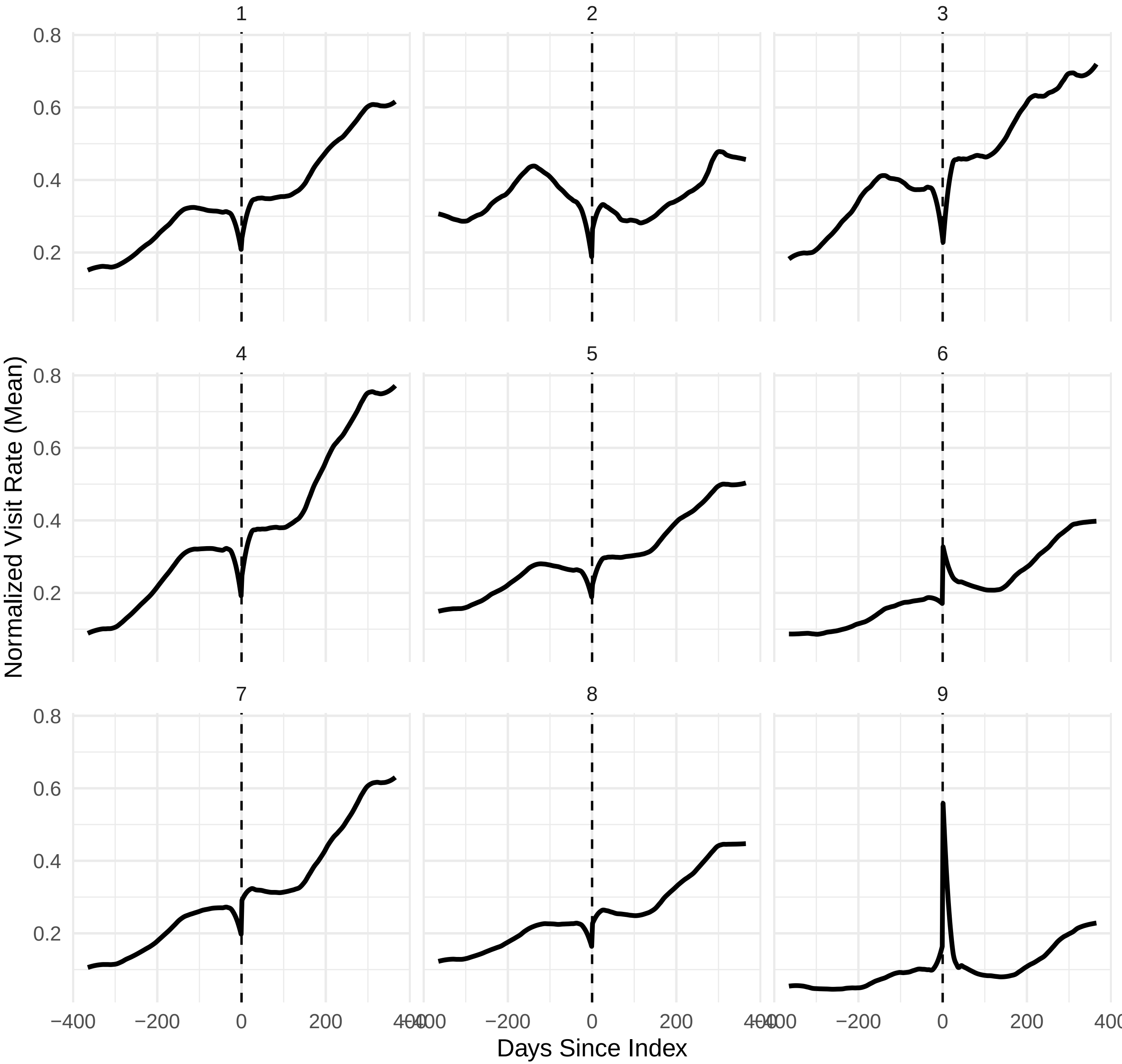

1
2
3
4
5
6
7
8
9
0.8
0.6
0.4
0.2
Normalized Visit Rate (Mean)
−400
−200
0
200
400
Days Since Index

Figure B8: k = 10

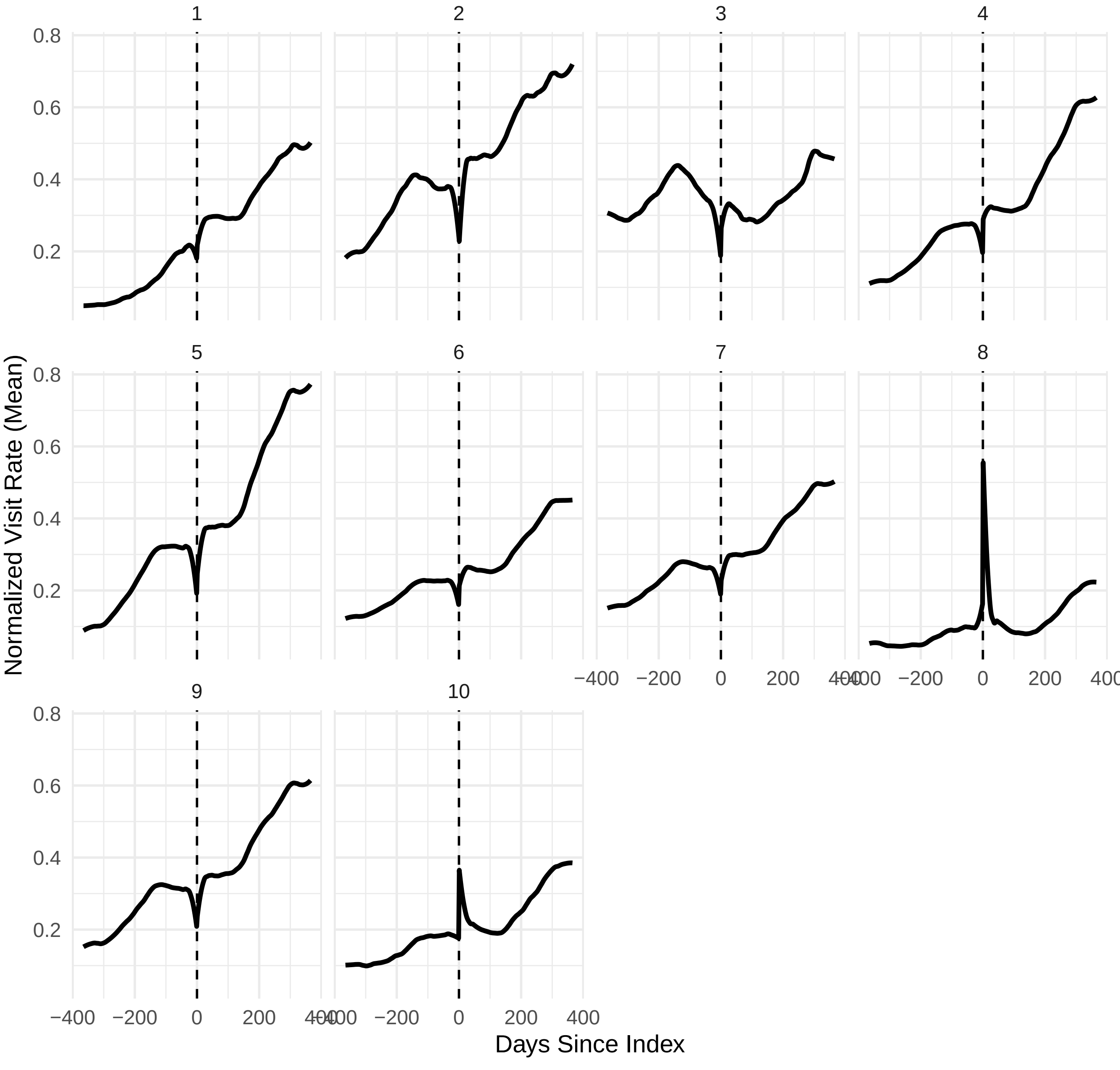

Figure B9: k = 11

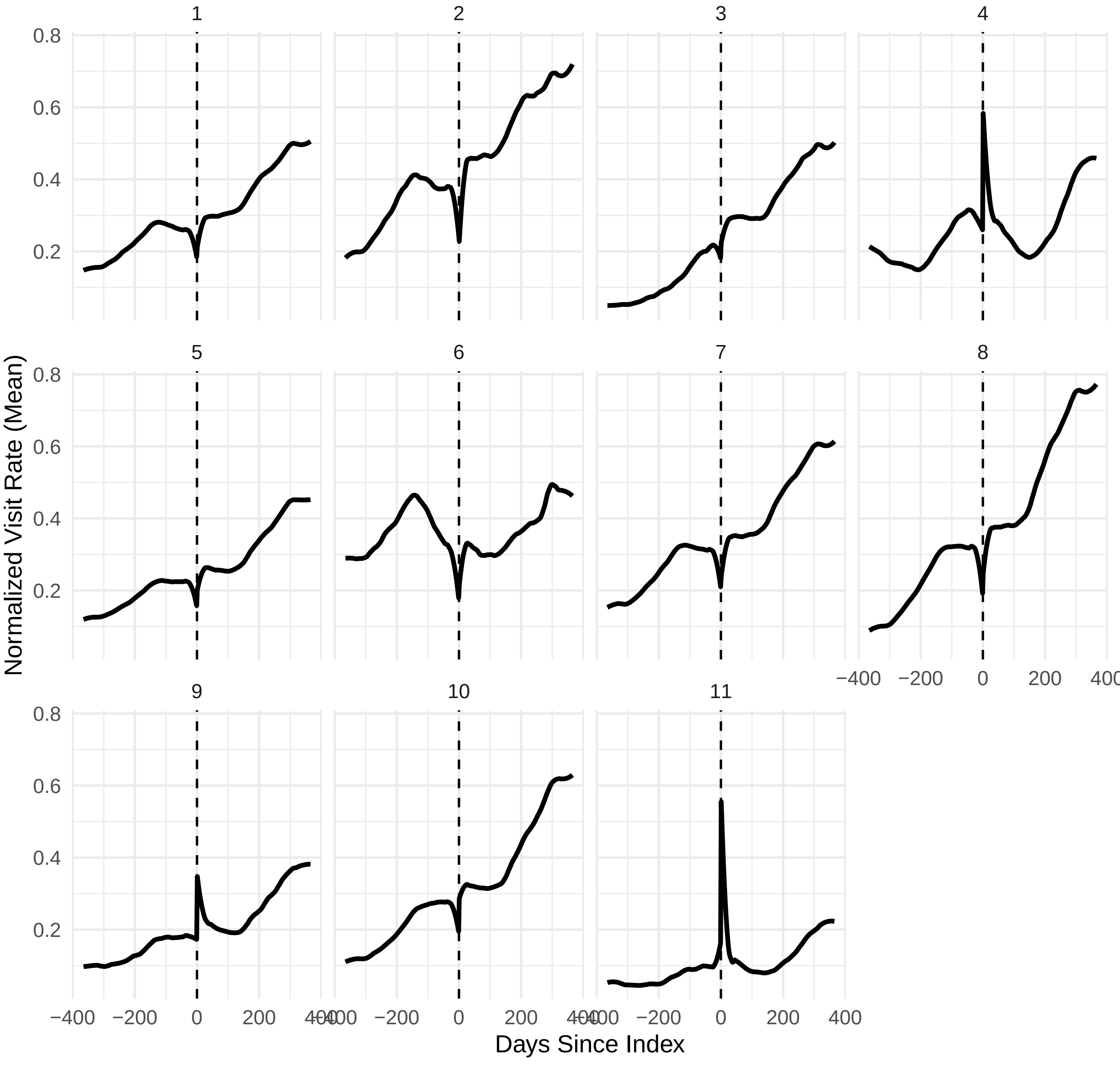

Figure B10: k = 12

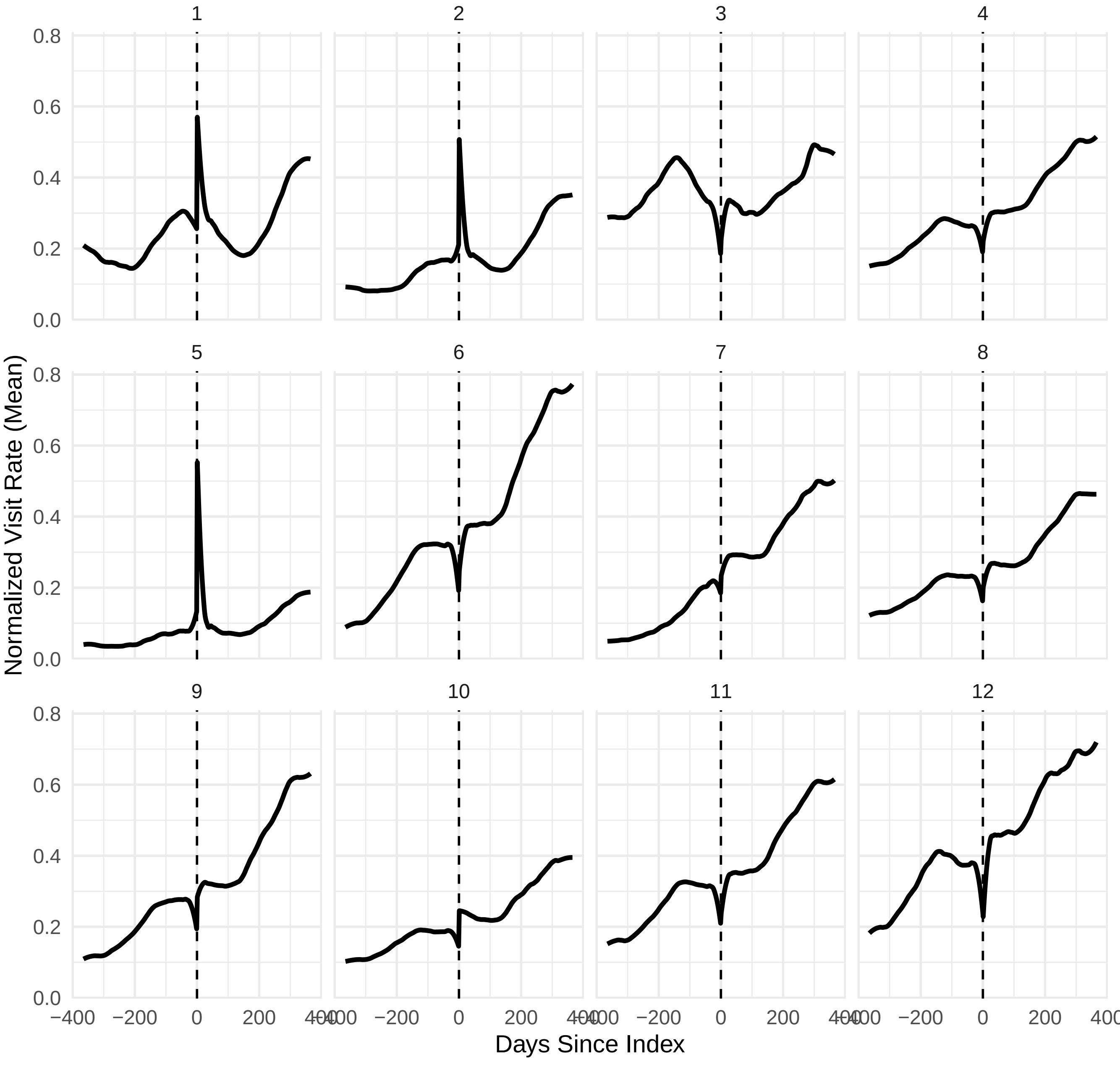

Figure B11: k = 13

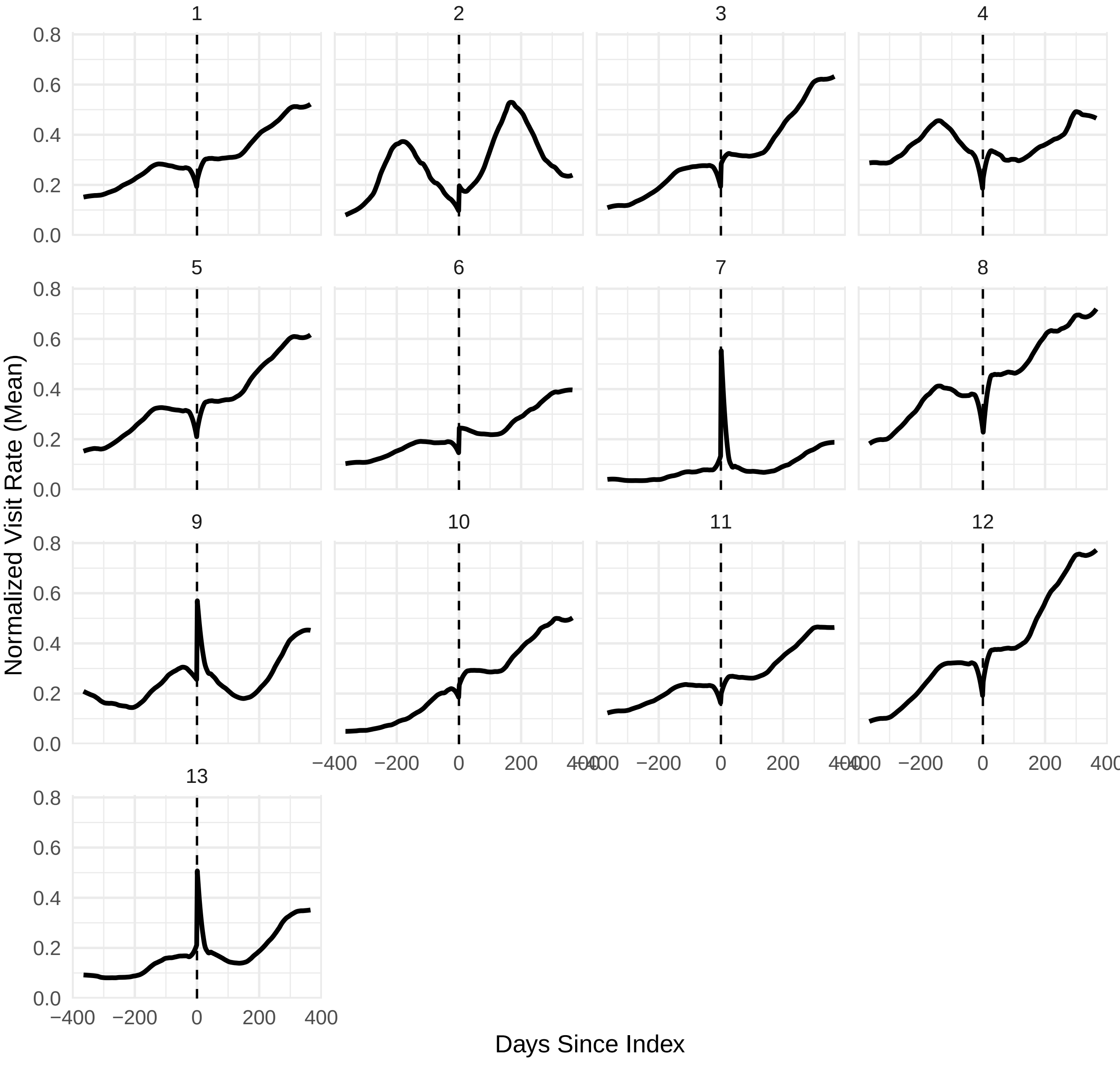

Figure B12: k = 14

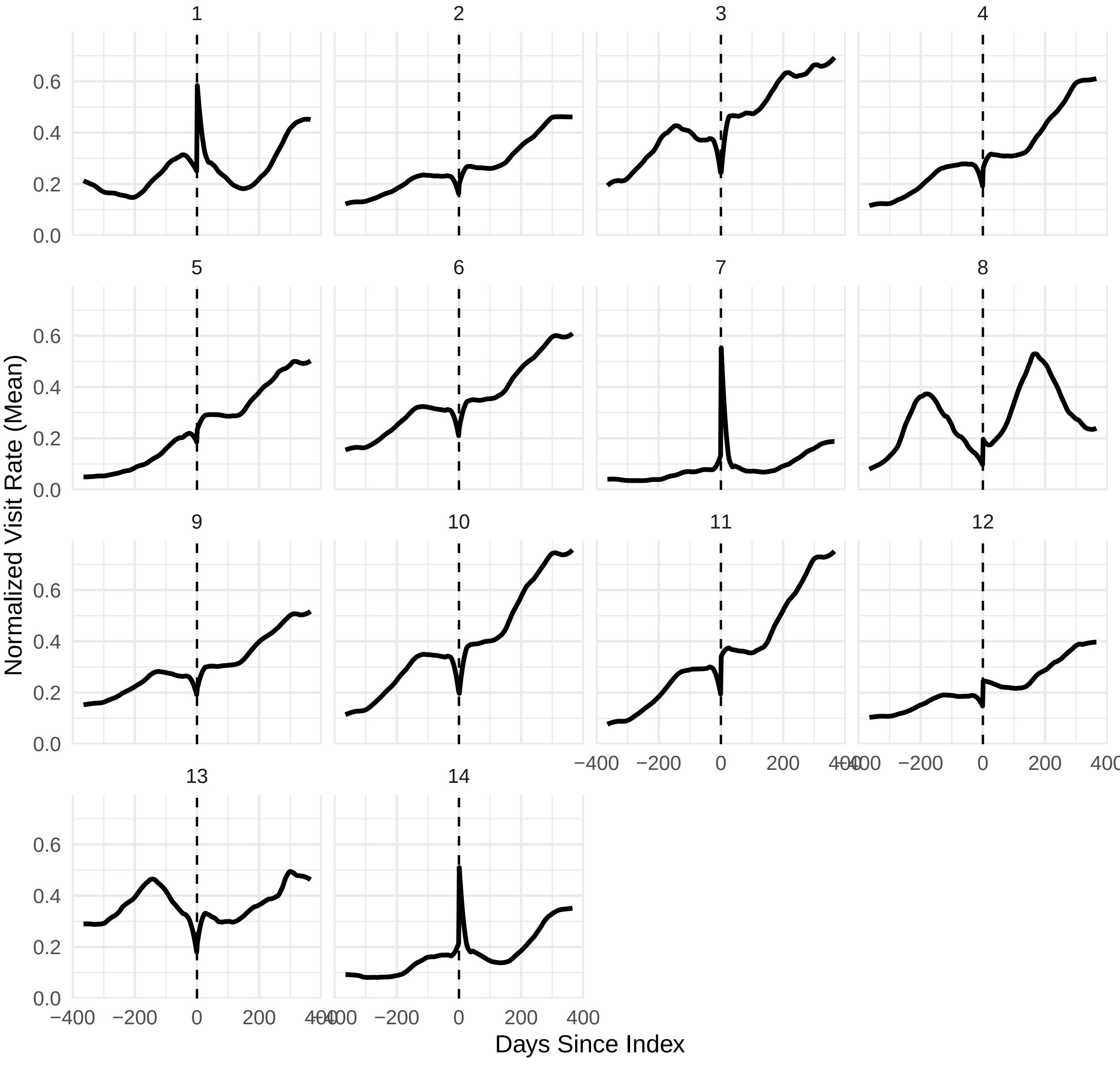

Figure B13: k = 15

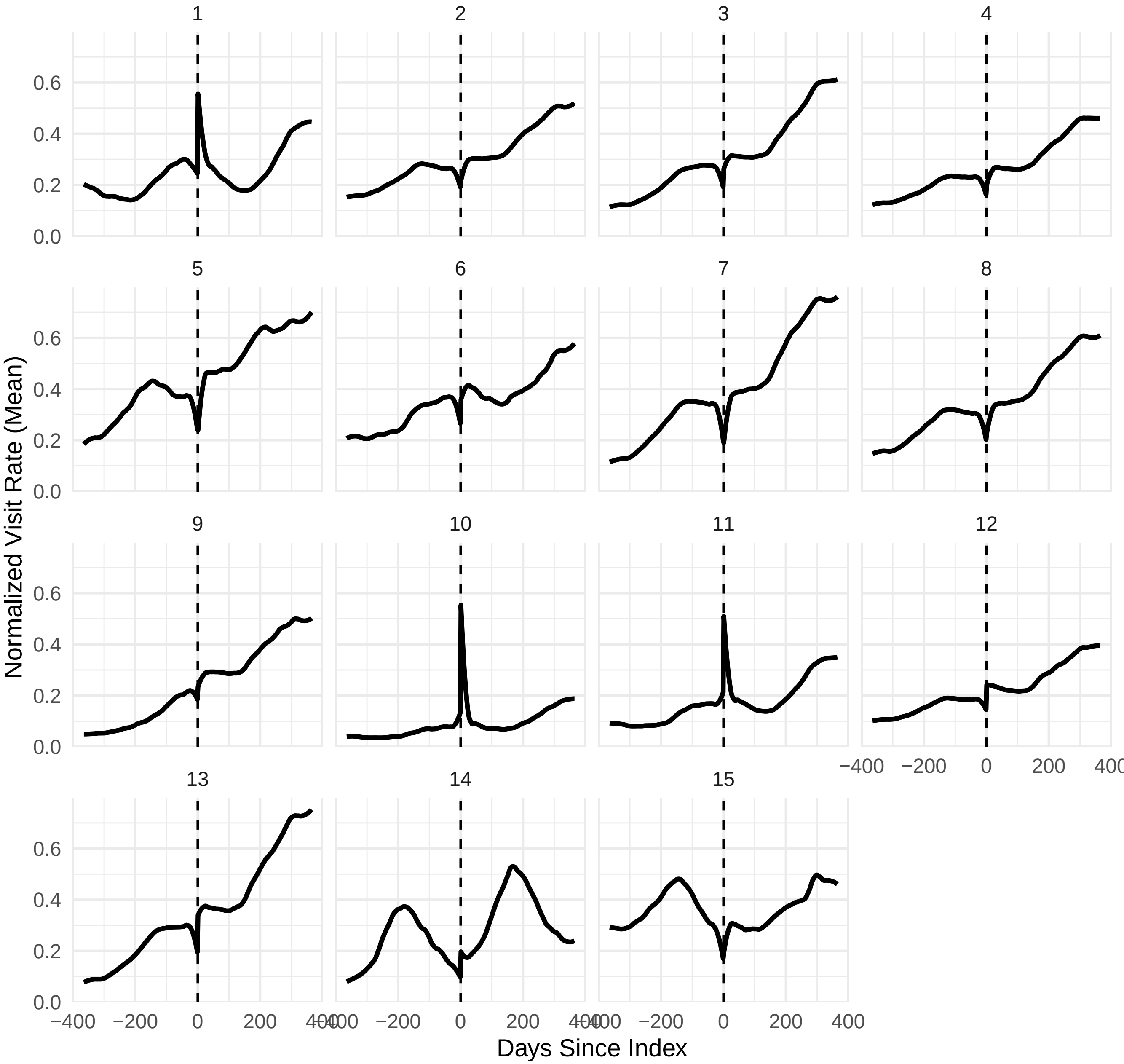

Figure B14: k = 16

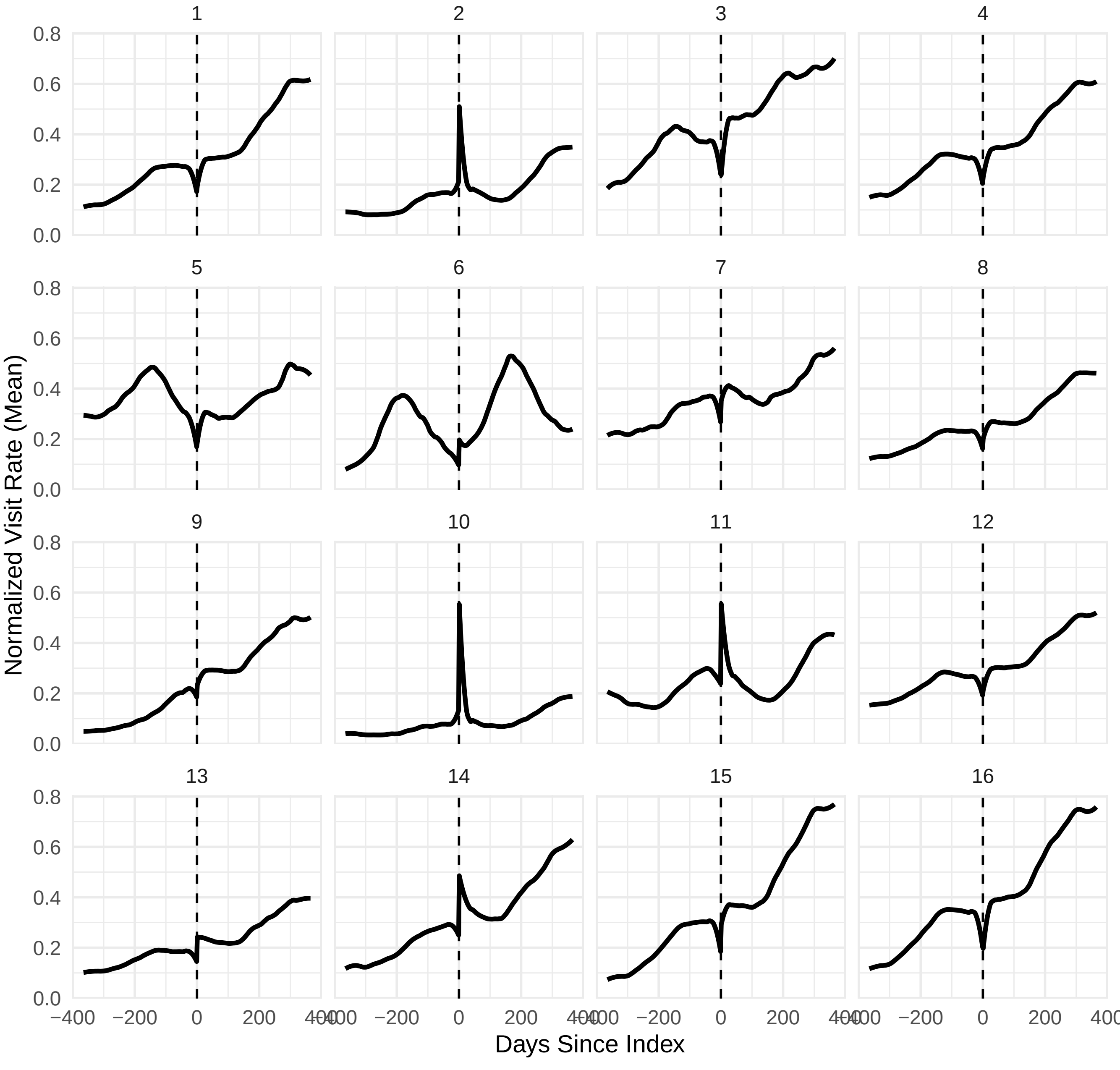

Figure B15: k = 17

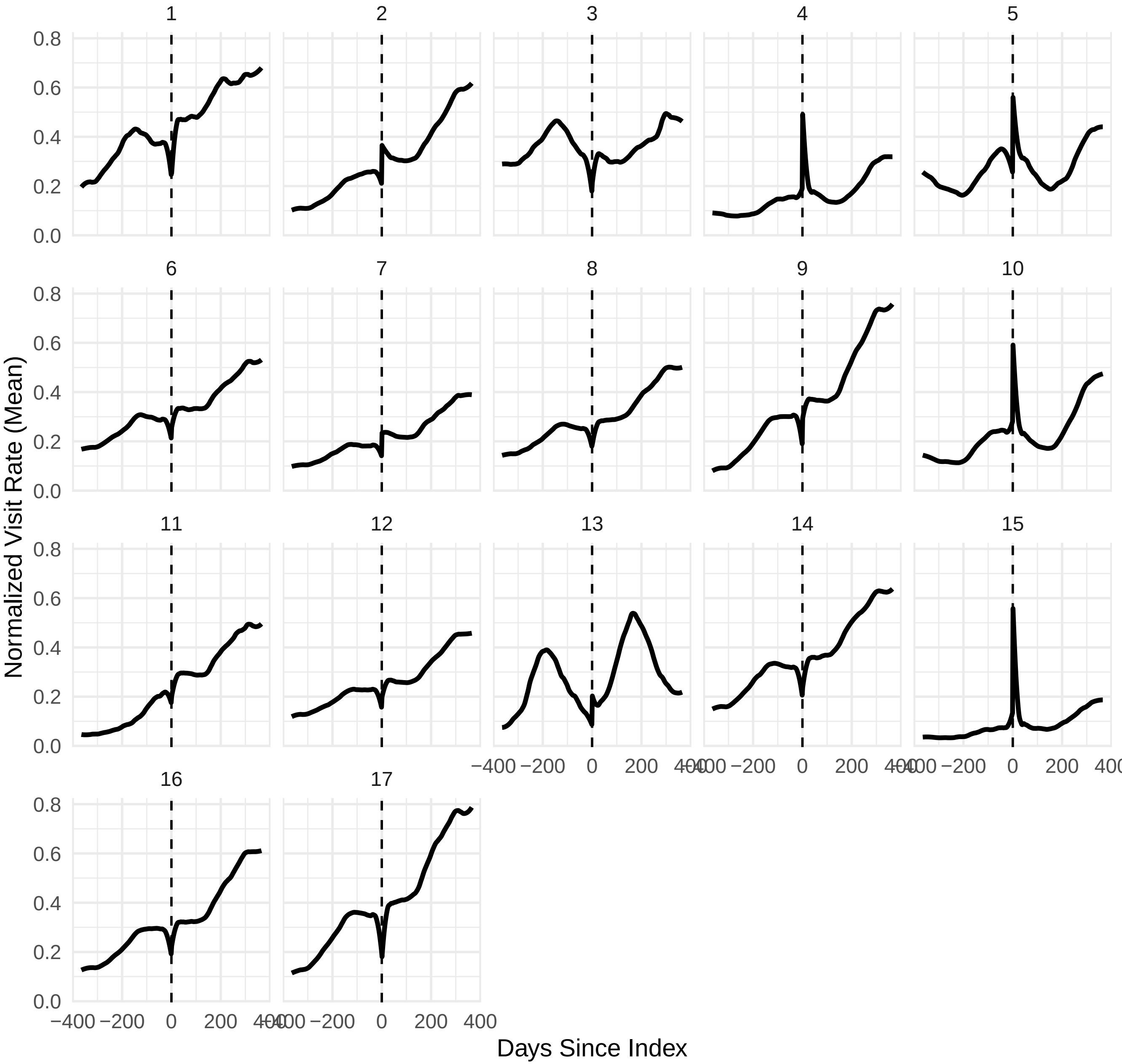

Figure B16: k = 18

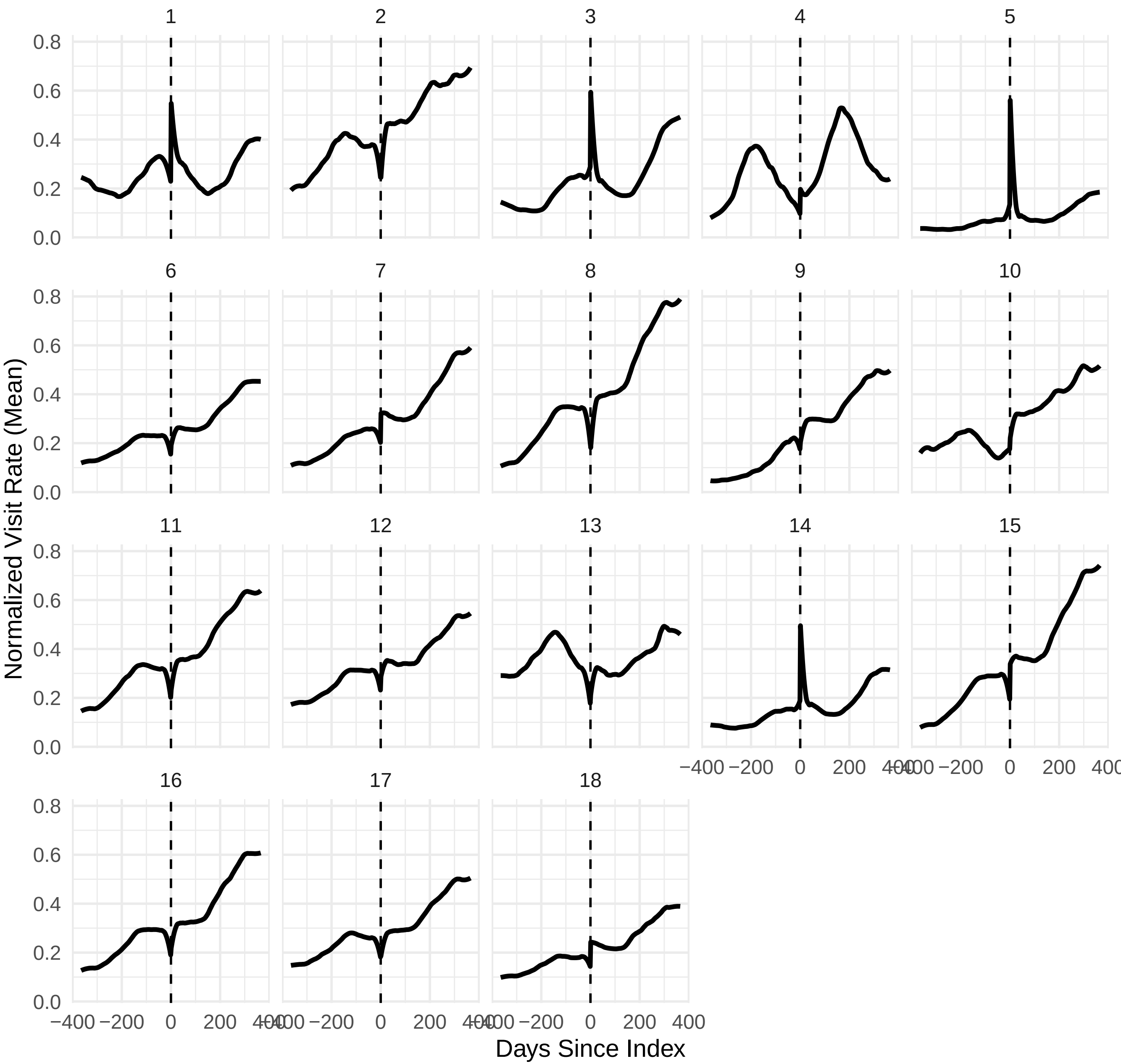

Figure B17: k = 19

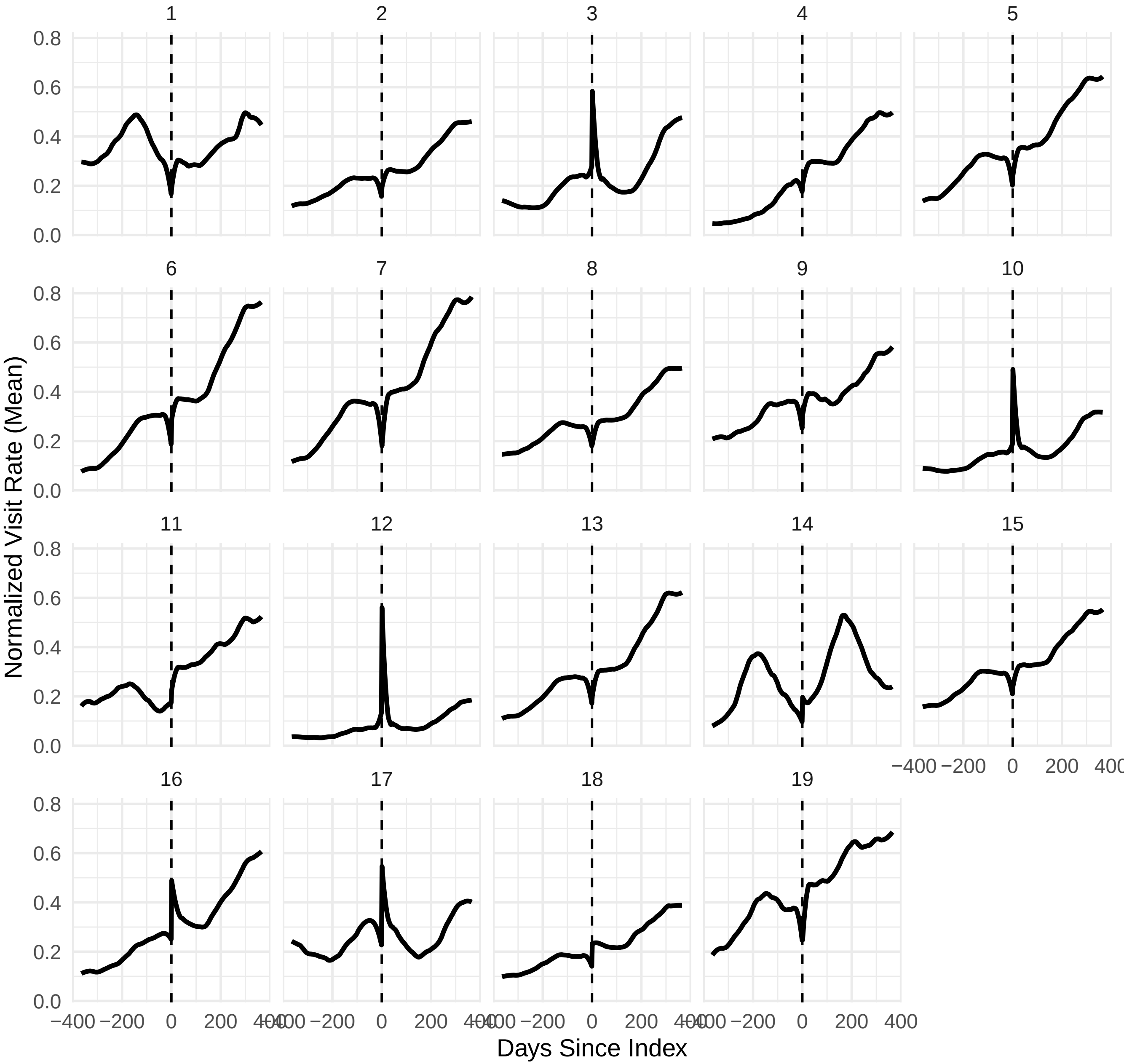

Figure B18: k = 20

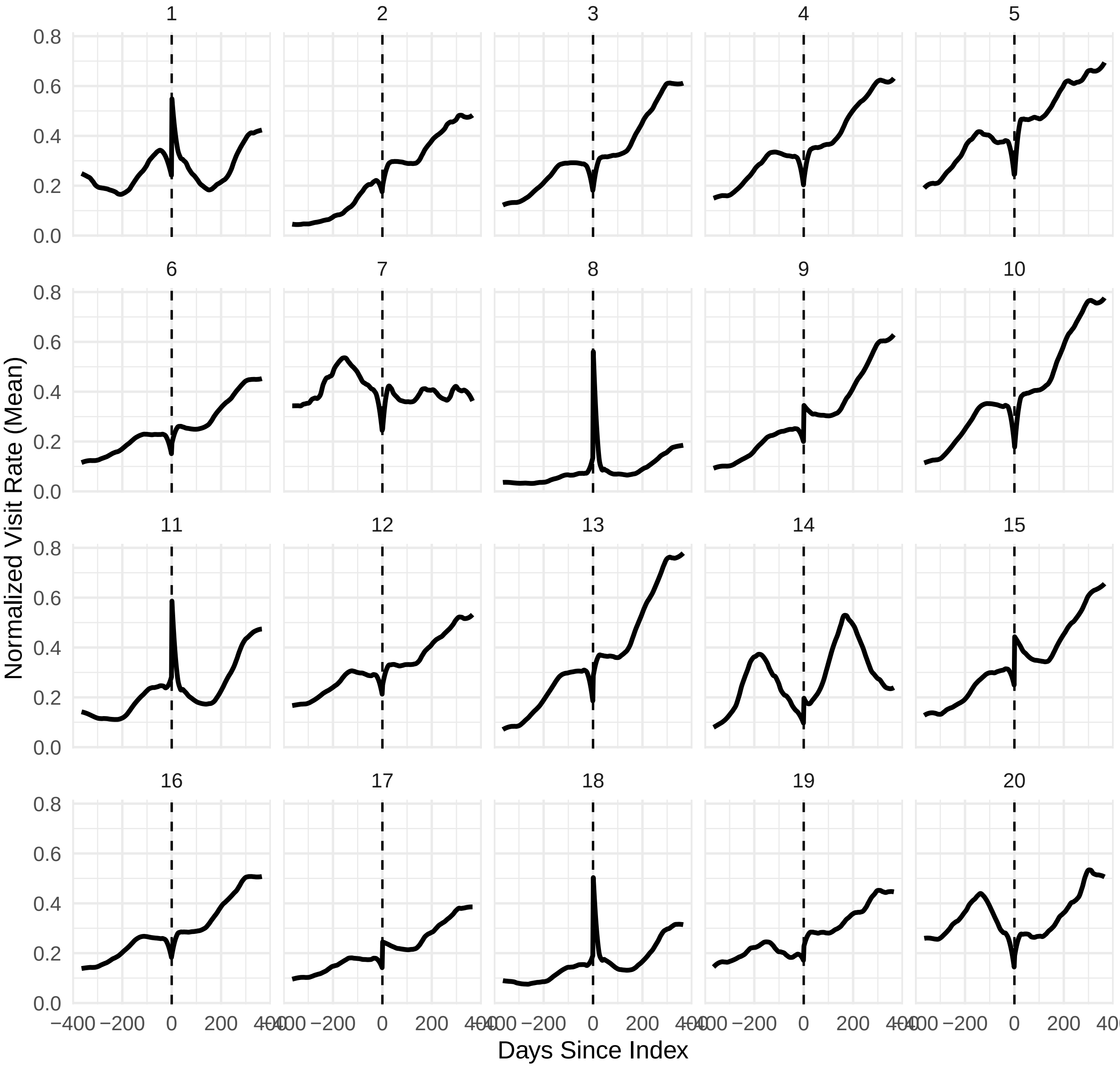

Figure B19: k = 21

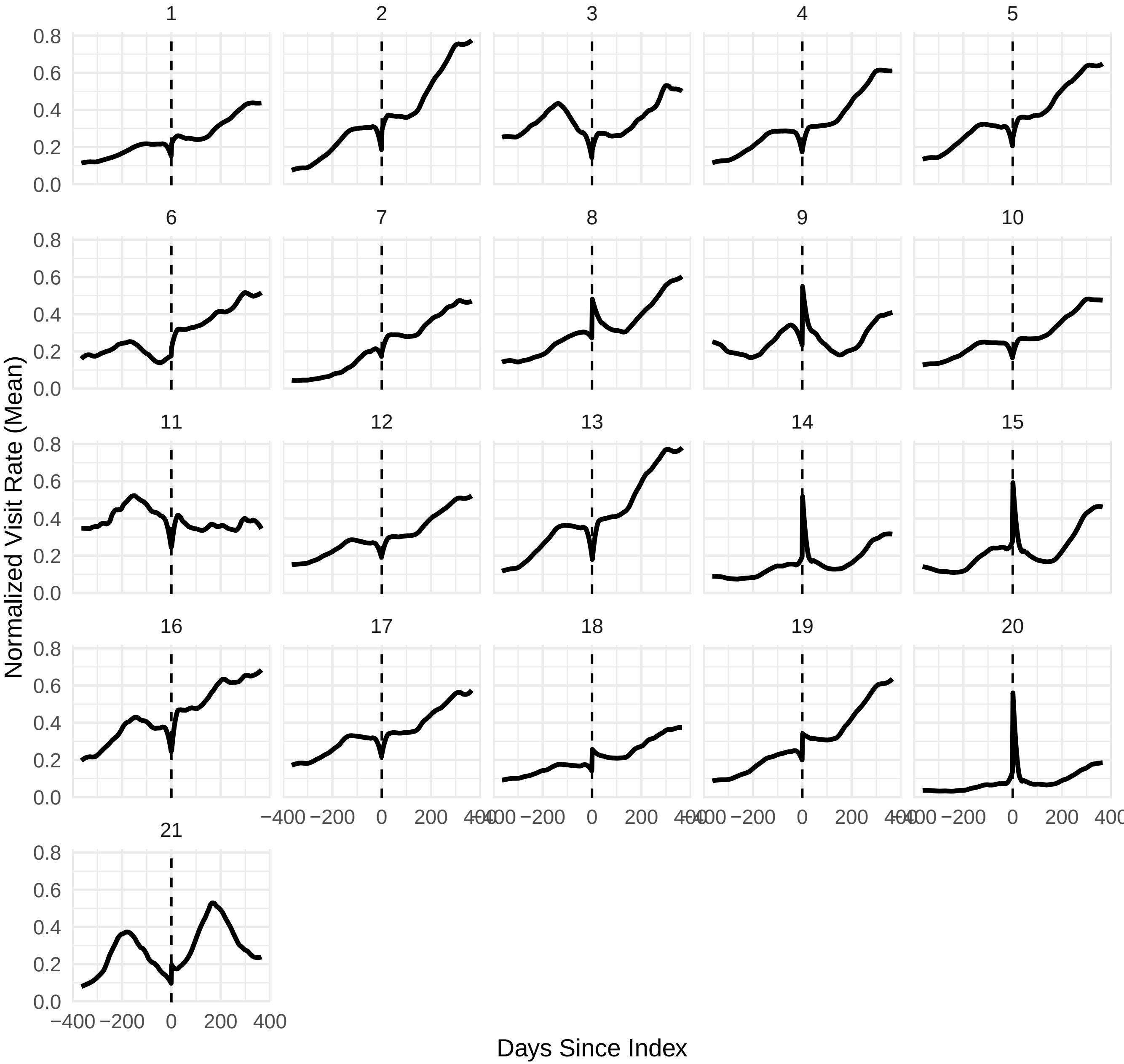

Figure B20: k = 22

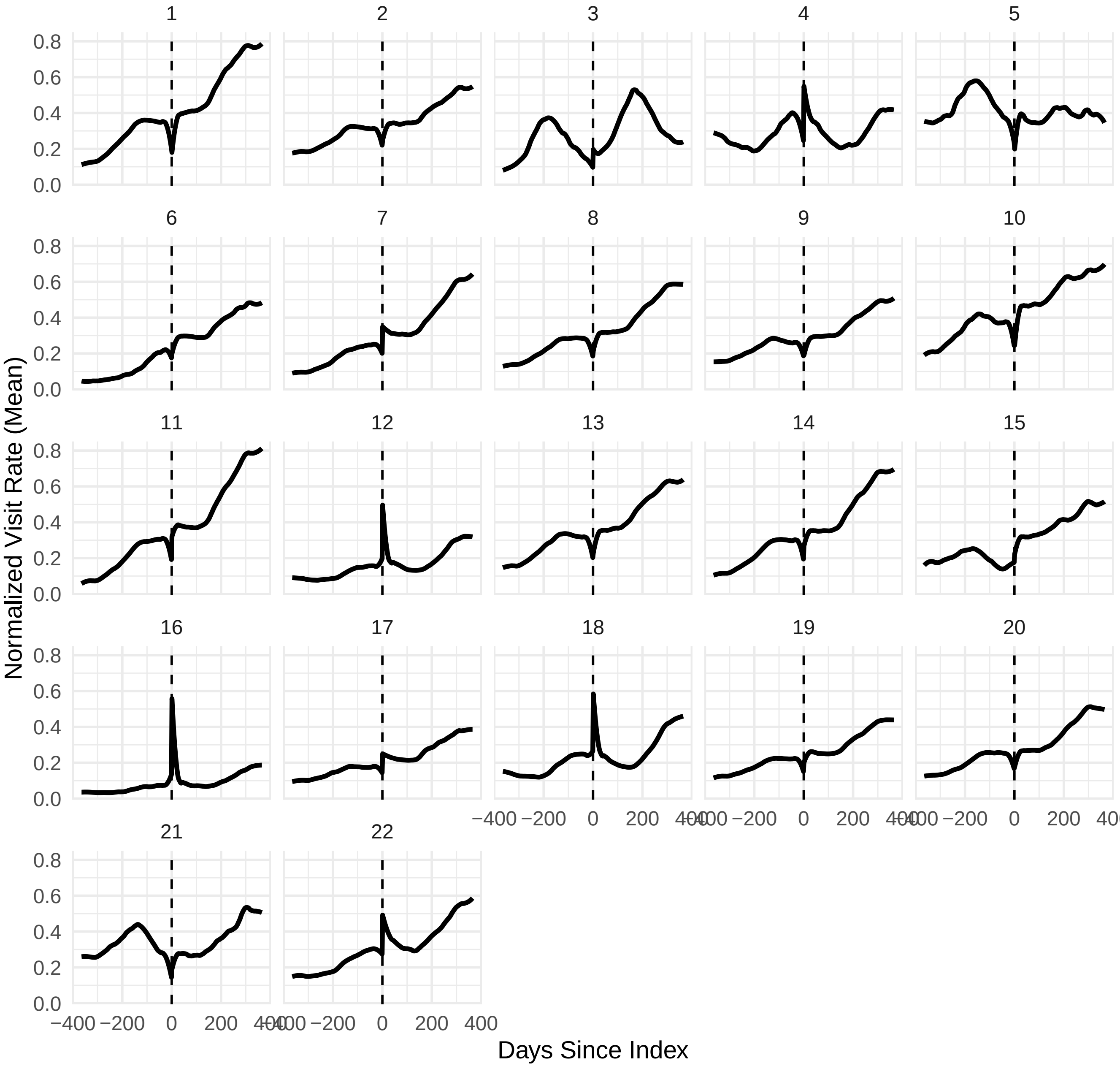

Figure B21: k = 23

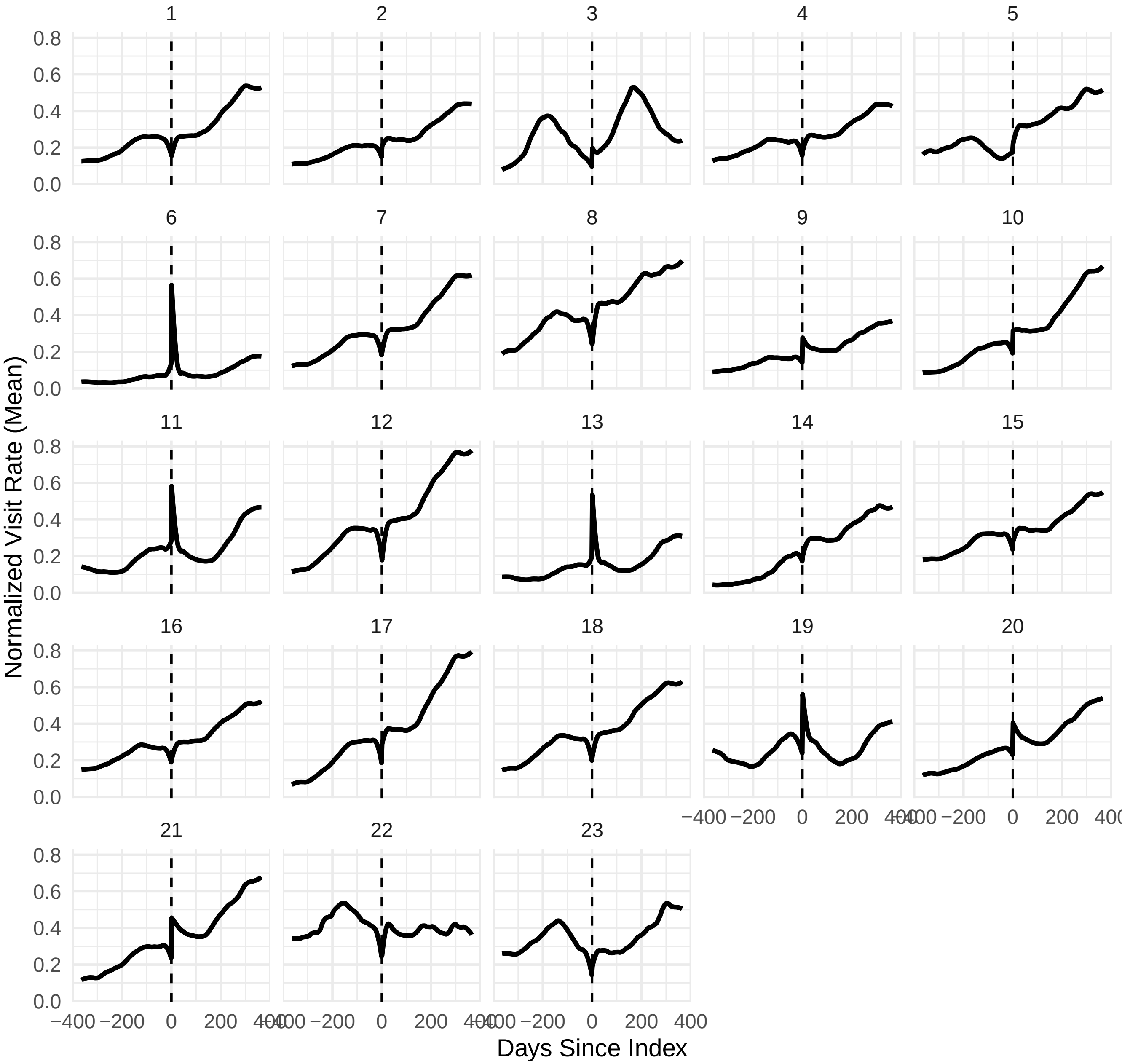

Figure B22: k = 24

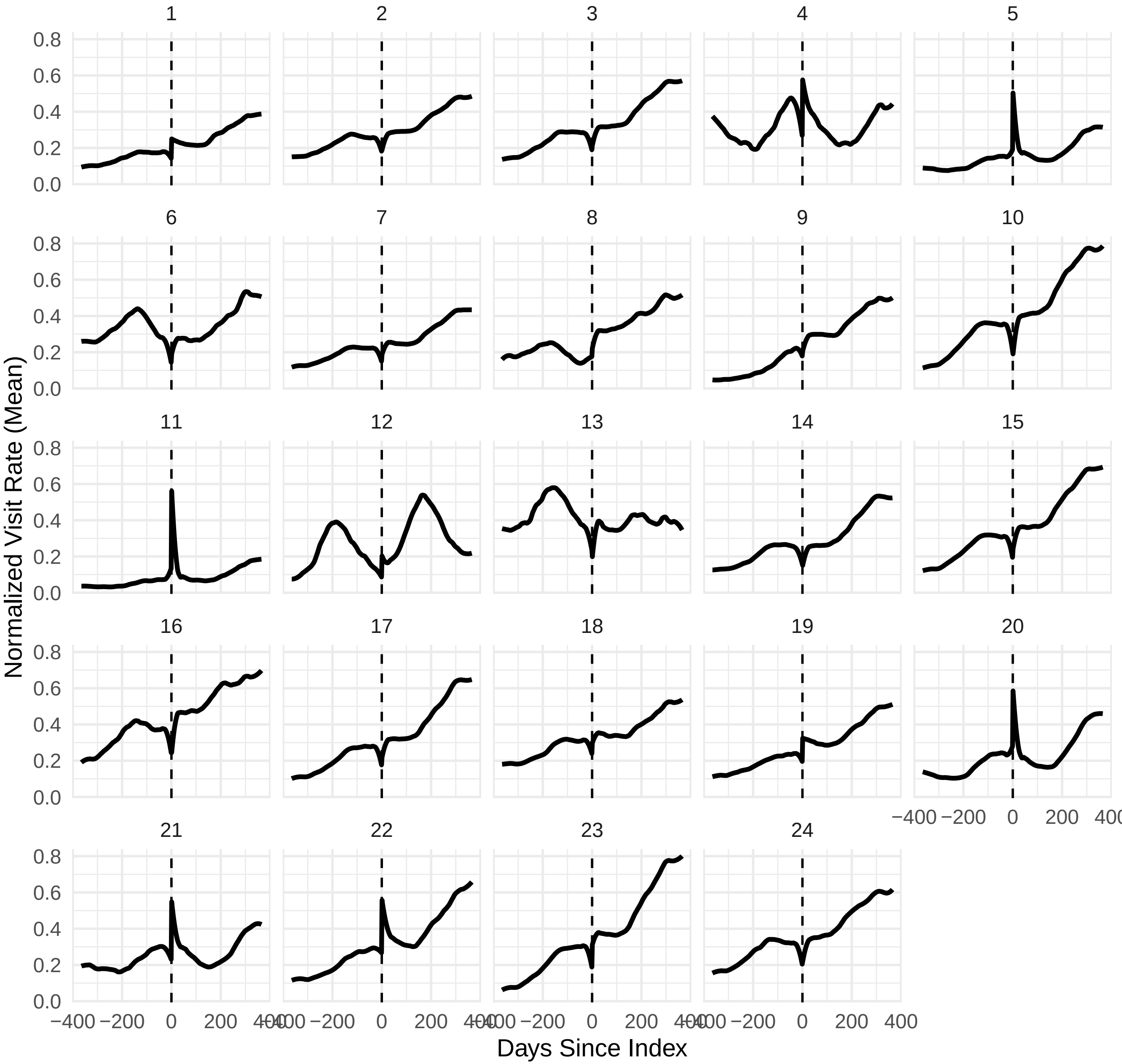

Figure B23: k = 25

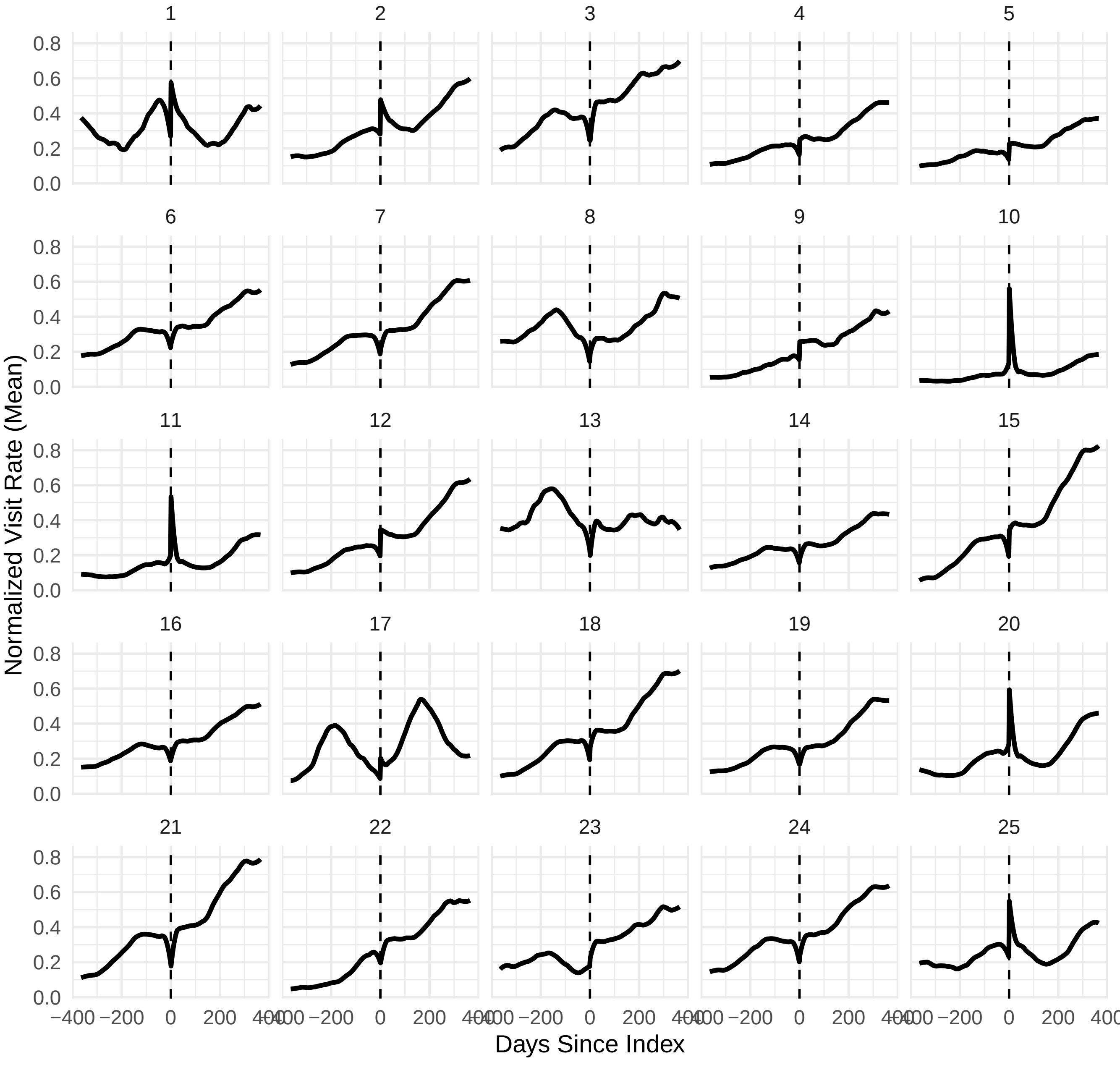

Figure B24: k = 26

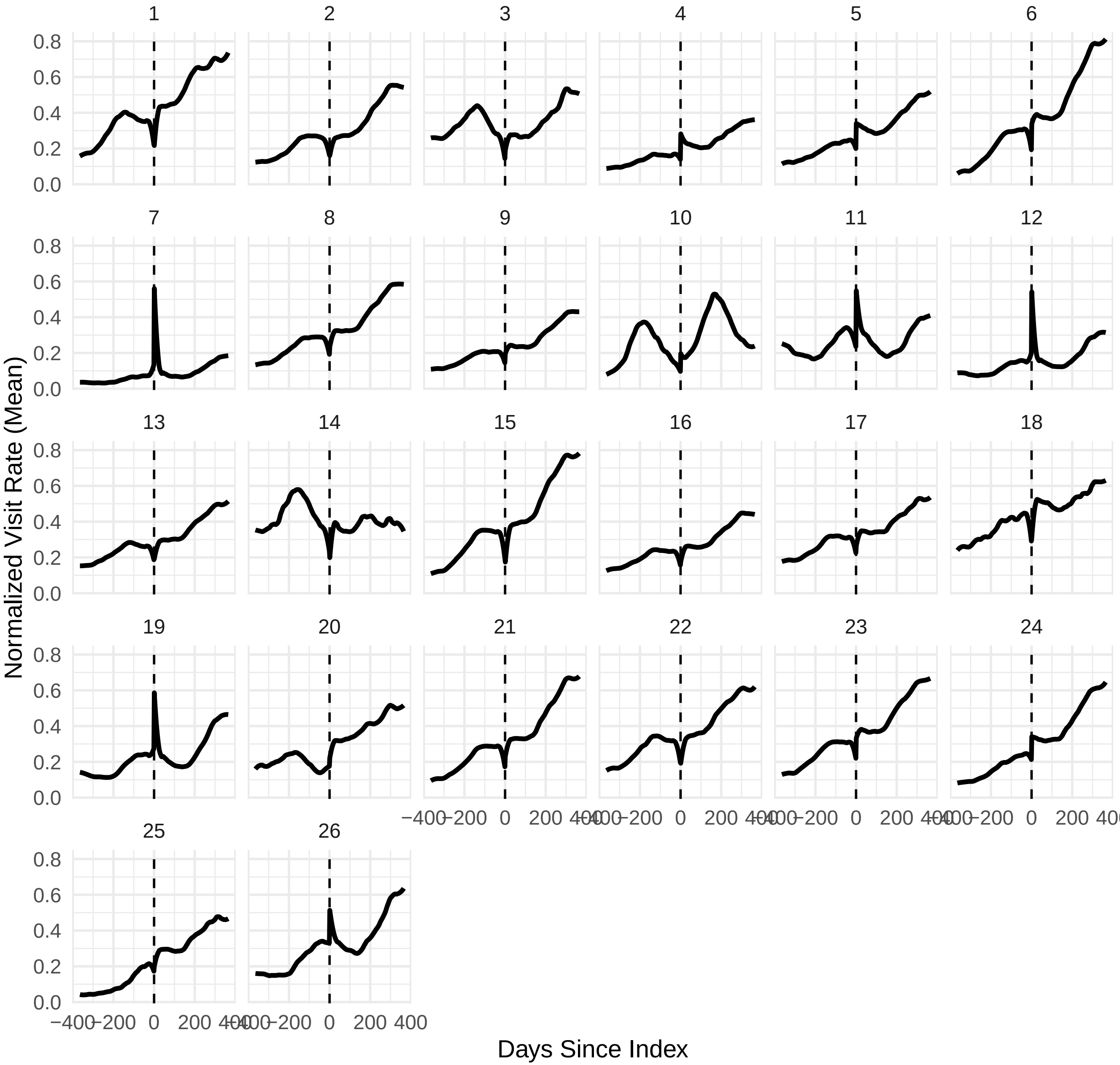

Figure B25: k = 27

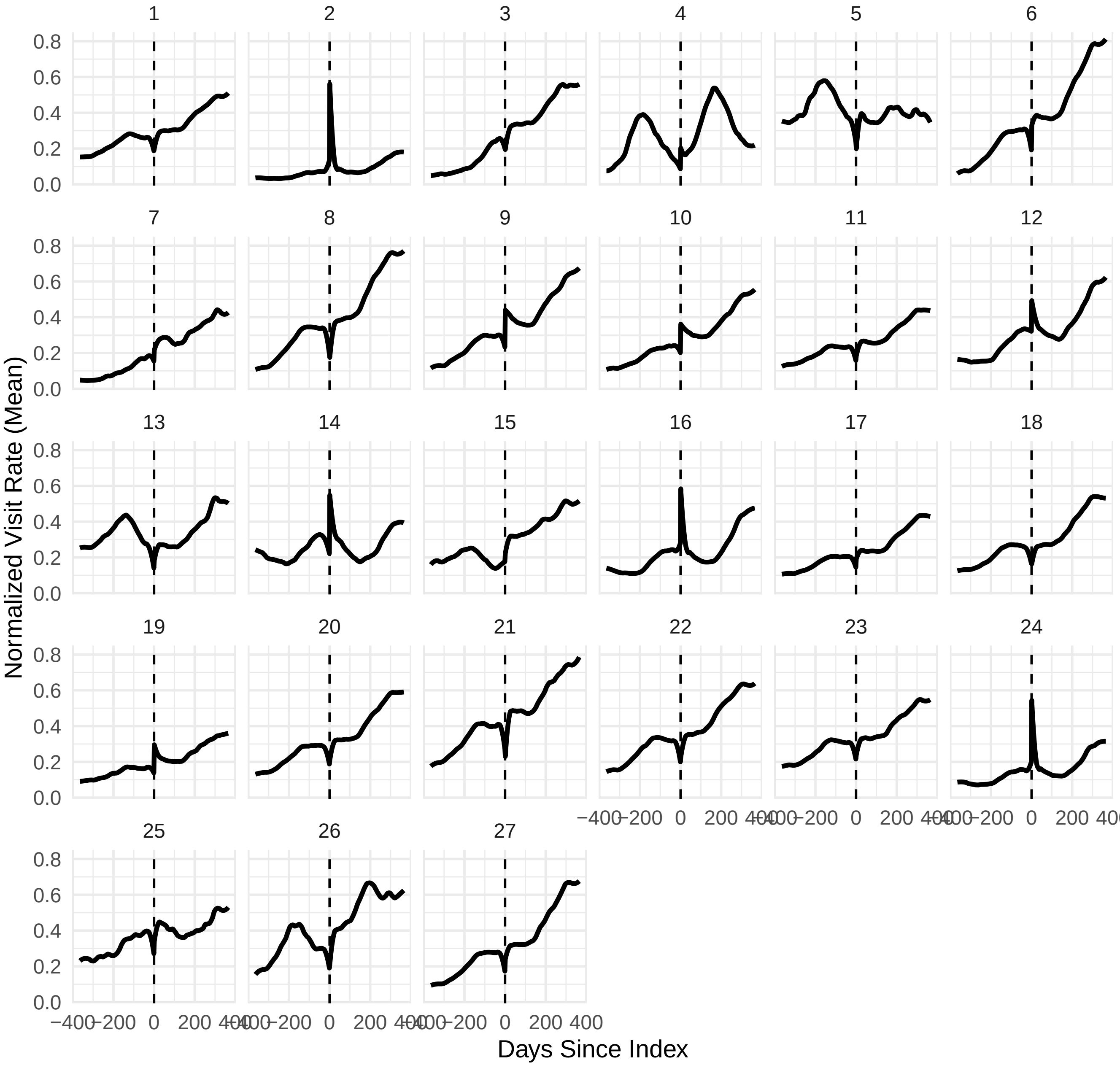

Figure B26: k = 28

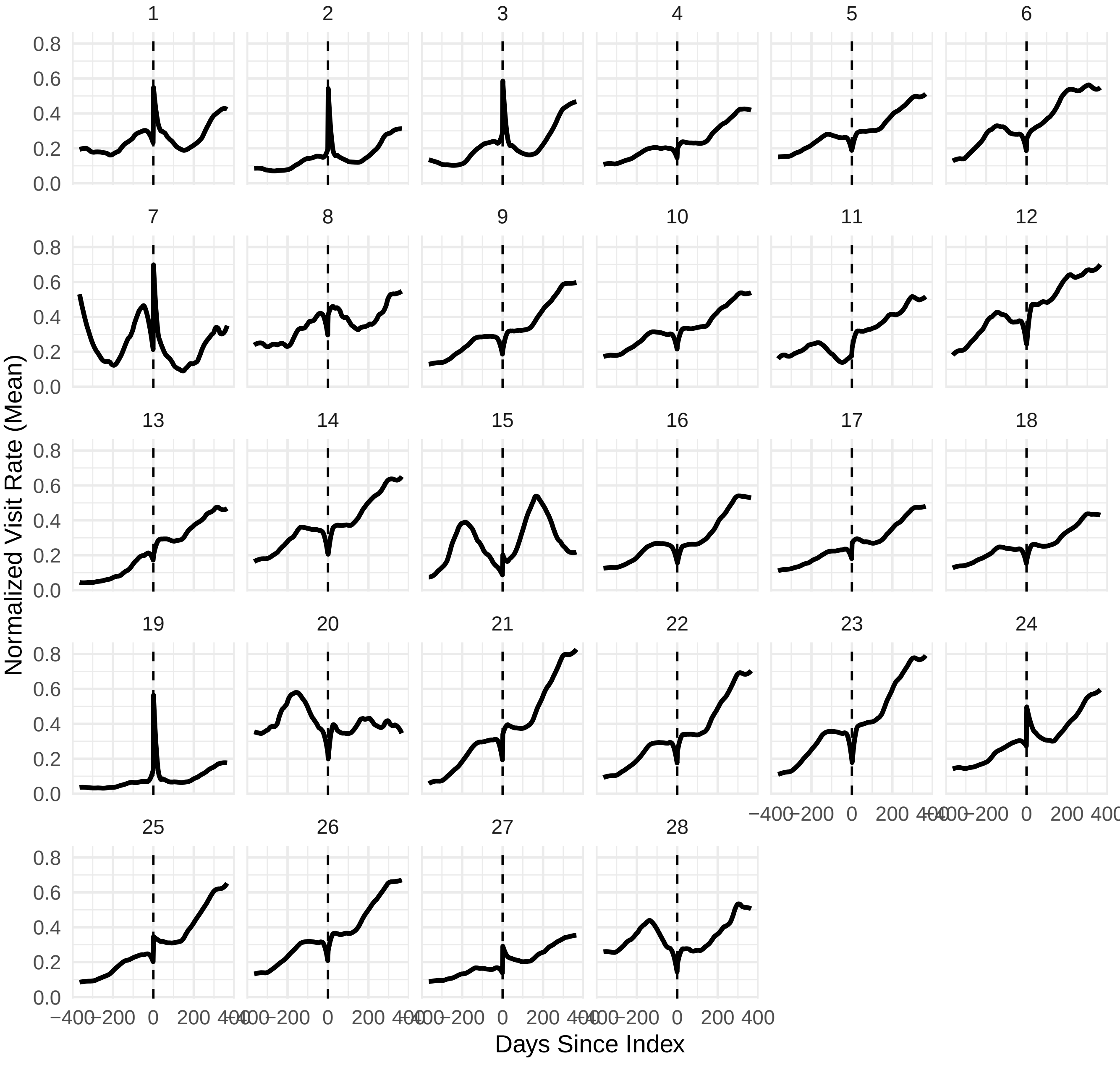

Figure B27: k = 29

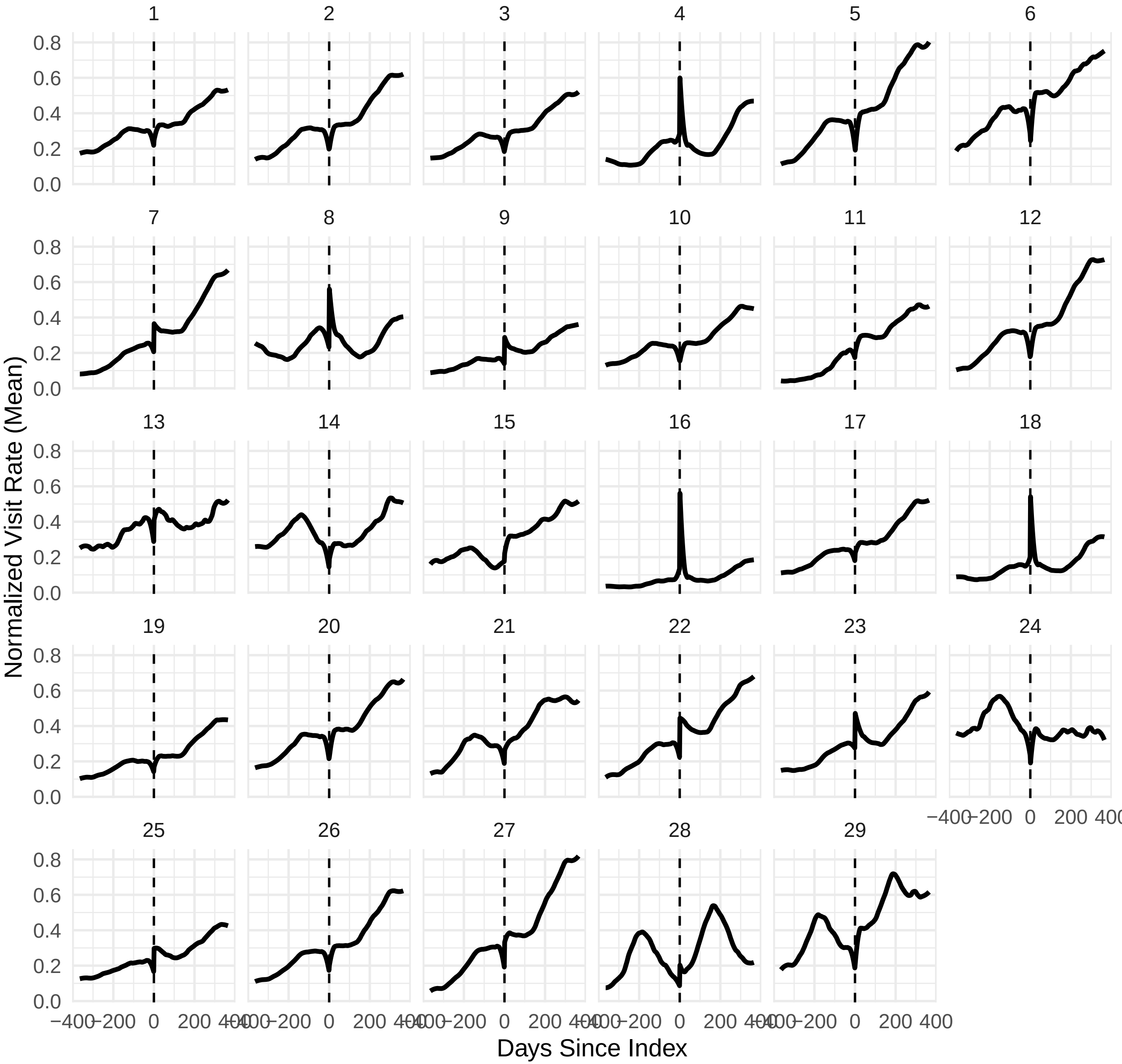

Figure B28: k = 30

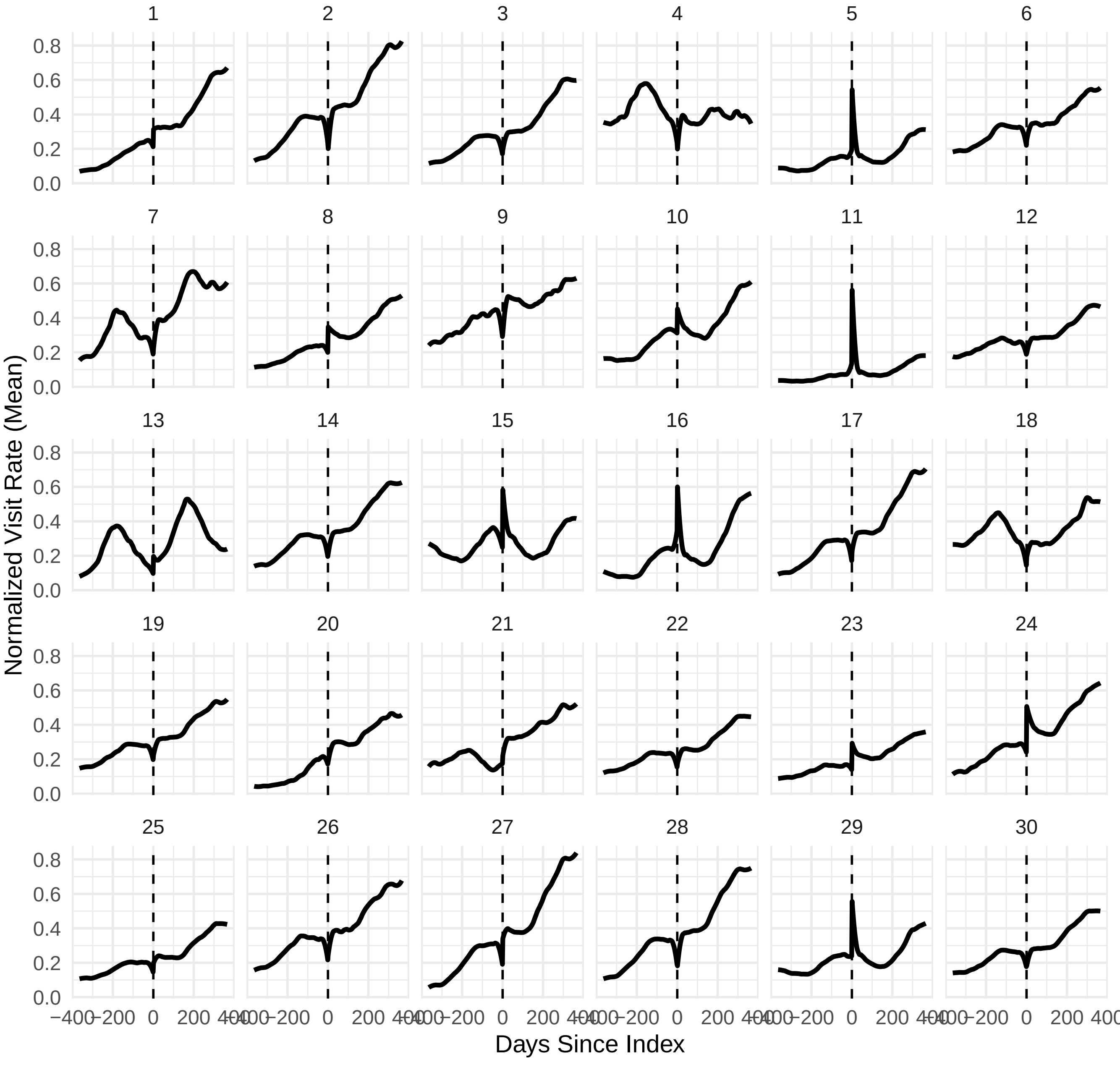

Supplement: Supplementary file 1 [file ofag629_supplementary_data.zip › FigB1_B28.pdf]
